# Myeloid C/EBPβ deficiency reshapes microglial gene expression and is protective in experimental autoimmune encephalomyelitis

**DOI:** 10.1186/s12974-017-0834-5

**Published:** 2017-03-16

**Authors:** Marta Pulido-Salgado, Jose M. Vidal-Taboada, Gerardo Garcia Diaz-Barriga, Joan Serratosa, Tony Valente, Paola Castillo, Jonathan Matalonga, Marco Straccia, Josep M. Canals, Annabel Valledor, Carme Solà, Josep Saura

**Affiliations:** 1 Department of Biomedicine, Biochemistry and Molecular Biology Unit, School of Medicine, University of Barcelona, IDIBAPS, Barcelona, Spain; 2 Department of Biomedicine, Histology Unit, School of Medicine, University of Barcelona, IDIBAPS, Barcelona, Spain; 3 Department of Cerebral Ischemia and Neurodegeneration, Institut d’Investigacions Biomèdiques de Barcelona, CSIC, IDIBAPS, Barcelona, Spain; 4 Department of Pathology, Hospital Clinic, ISGlobal, CRESIB, Barcelona, Spain; 5 Department of Physiology and Immunology, School of Biology, University of Barcelona, Barcelona, Catalonia Spain; 6 Institute of Neurosciences, University of Barcelona, Barcelona, Spain

**Keywords:** Transcription factor, Neuroinflammation, Lipopolysaccharide, Interferon γ, RNA sequencing

## Abstract

**Background:**

CCAAT/enhancer binding protein β (C/EBPβ) is a transcription factor that regulates the expression of important pro-inflammatory genes in microglia. Mice deficient for C/EBPβ show protection against excitotoxic and ischemic CNS damage, but the involvement in this neuroprotective effect of the various C/EBPβ-expressing cell types is not solved. Since C/EBPβ-deficient microglia show attenuated neurotoxicity in culture, we hypothesized that specific C/EBPβ deficiency in microglia could be neuroprotective in vivo. In this study, we have tested this hypothesis by generating mice with myeloid C/EBPβ deficiency.

**Methods:**

Mice with myeloid C/EBPβ deficiency were generated by crossing LysMCre and C/EBPβ^fl/fl^ mice. Primary microglial cultures from C/EBPβ^fl/fl^ and LysMCre-C/EBPβ^fl/fl^ mice were treated with lipopolysaccharide ± interferon γ (IFNγ) for 6 h, and gene expression was analyzed by RNA sequencing. Gene expression and C/EBPβ deletion were analyzed in vivo in microglia isolated from the brains of C/EBPβ^fl/fl^ and LysMCre-C/EBPβ^fl/fl^ mice treated systemically with lipolysaccharide or vehicle. Mice of LysMCre-C/EBPβ^fl/fl^ or control genotypes were subjected to experimental autoimmune encephalitis and analyzed for clinical signs for 52 days. One- or two-way ANOVA or Kruskal–Wallis with their appropriate post hoc tests were used.

**Results:**

LysMCre-C/EBPβ^fl/fl^ mice showed an efficiency of C/EBPβ deletion in microglia of 100 and 90% in vitro and in vivo, respectively. These mice were devoid of female infertility, perinatal mortality and reduced lifespan that are associated to full C/EBPβ deficiency. Transcriptomic analysis of C/EBPβ-deficient primary microglia revealed C/EBPβ-dependent expression of 1068 genes, significantly enriched in inflammatory and innate immune responses GO terms. In vivo, microglial expression of the pro-inflammatory genes Cybb, Ptges, Il23a, Tnf and Csf3 induced by systemic lipopolysaccharide injection was also blunted by C/EBPβ deletion. CNS expression of C/EBPβ was upregulated in experimental autoimmune encephalitis and in multiple sclerosis samples. Finally, LysMCre-C/EBPβ^fl/fl^ mice showed robust attenuation of clinical signs in experimental autoimmune encephalitis.

**Conclusion:**

This study provides new data that support a central role for C/EBPβ in the biology of activated microglia, and it offers proof of concept for the therapeutic potential of microglial C/EBPβ inhibition in multiple sclerosis.

**Electronic supplementary material:**

The online version of this article (doi:10.1186/s12974-017-0834-5) contains supplementary material, which is available to authorized users.

## Background

Neuroinflammation is a cellular and molecular response of the CNS to a variety of cues, including pathogens, abnormal protein deposits, toxic metabolites, trauma, autoimmunity or massive cell damage, which has an inflammatory character. Microglia, the innate immune cells of the brain, are the main cell type involved in this response, with astrocytes also playing a prominent role. Chronic and/or exacerbated neuroinflammation is associated with the production of potentially cytotoxic molecules, such as oxygen and nitrogen free radicals, pro-inflammatory cytokines or proteases [1], and this is thought to contribute to neurodegeneration in a growing list of neurological and psychiatric disorders [2].

In neuroinflammation, microglia undergoes massive changes in gene expression that are regulated by a reduced number of transcription factors. There is strong evidence showing that pro-inflammatory gene expression by activated microglia is regulated by the transcription factors NF-κB, AP-1, CREB, STATs, C/EBPβ and C/EBPδ, whereas PPARγ and Nrf2 are two of the most important transcription factors regulating anti-inflammatory programs in microglia [3]. Because of their bottleneck position, transcription factors are potential targets to pharmacologically modulate whole cellular programs such as the neuroinflammatory one in activated microglia.

The present study is focused on the role of C/EBPβ, a transcription factor of the b-zip class, in microglial activation. The C/EBPβ gene codes for a single mRNA which can be translated into three protein isoforms, named Full, LAP and LIP, by use of alternative in-frame translation initiation codons [4]. C/EBPβ levels increase in activated microglia [5] where it regulates the expression of key pro-inflammatory genes [3, 6–8]. In vitro and in vivo data suggest a neuroprotective potential for C/EBPβ inhibition in microglia. Thus, neurotoxicity elicited by activated microglia in neuronal–microglial co-cultures is abolished by the absence of C/EBPβ in microglia [3]. Also, C/EBPβ-deficient mice show reduced neuronal death and neurological deficits caused by ischemic [9] or excitotoxic [7] damage in vivo. Even though these in vivo findings are promising, ubiquitous C/EBPβ inhibition is probably undesirable as a therapeutic strategy because C/EBPβ plays important roles in other cell types, such as adipocytes, hepatocytes or neurons [4], that could be compromised. Our hypothesis is that microglia-targeted C/EBPβ inhibition could be a safe and effective approach to attenuate neuroinflammation-driven neurodegeneration. In order to test this hypothesis, we generated transgenic mice with myeloid-specific C/EBPβ deficiency using a Cre-LoxP system and transcriptomically profiled C/EBPβ-deficient microglia. To explore the effects of C/EBPβ myeloid deficiency in a pathological context, we analyzed the clinical response of these animals to experimental autoimmune encephalomyelitis (EAE), an animal model of multiple sclerosis. The data here presented show that microglial C/EBPβ absence results in remarkable effects on pro-inflammatory gene expression programs and in the amelioration of EAE symptomatic phenotype.

## Methods

### Human samples

Postmortem human temporal cortex samples were supplied by the Neurological Tissue Bank of the Biobanc-Hospital Clínic-IDIBAPS (Barcelona, Spain): healthy controls (*n* = 4; 2 ♀, 2 ♂; age, 66–81 years; postmortem delay, 3.5–23.5 h) and patients with a diagnosis of primary progressive MS (*n* = 5; 2 ♀, 3 ♂; age, 46–68 years; postmortem delay, 3–8 h). Protein was extracted from frozen tissue blocks [10], and it was processed for Western blot analysis as described below.

### Animals

Mice with myeloid C/EBPβ deficiency were generated by crossing transgenic mice expressing Cre-recombinase under the lysozyme M (LysM) promoter (B6.129P2-Lyz2^tm1(cre)Ifo^/J 004781, Jackson Laboratories) with mice carrying a C/EBPβ gene flanked by LoxP sites [11] kindly donated by Prof Esta Sterneck (National Cancer Institute, Frederick, MD). These mice are referred to as LysMCre-C/EBPβ^fl/fl^ in the manuscript. LysMCre, C/EBPβ^fl/fl^ and LysMCre-C/EBPβ^fl/fl^ colonies, all on a C57BL/6 background, were intercrossed every three generations to diminish inbreeding. Mouse tail samples were used for genotyping the presence of the Cre transgene and the floxed C/EBPβ alleles. DNA was extracted and purified using KAPA Mouse Genotyping Kit (Kapa Biosystems). One microliter of the supernatant was used for polymerase chain reaction (PCR) amplification with specific primers for the LysMCre (5′-CCCAGAAATGCCAGATTACG-3′ mutant, 5′-CTTGGGCTGCCAGAATTTCTC-3′ common, 5′-TTACAGTCGGCCAGGCTGAC-3′ wild type) as described in the JAX mouse database (The Jackson Laboratory) and the C/EBPβ floxed allele (forward: 5-GAGCCACCGCGTCCTCCAGC-′3′, reverse: 5′-GGTCGGTGCGCGTCATTGCC-3′). PCR products were loaded on 2% agarose gels to check the presence (700 bp) or absence (350 bp) of the LysMCre transgene and the wild-type (240 bp) or floxed (320 bp) C/EBPβ allele. The mice were breed and housed under specific pathogen-free conditions in the animal facilities at the School of Medicine, University of Barcelona.

### Primary microglial cultures

Microglial cells were isolated from primary mixed glial cultures prepared from P1-P3 C/EBPβ^fl/fl^ and LysMCre-C/EBPβ^fl/fl^ mice. The brains were dissected, the meninges removed and the cortices digested with 0.25% trypsin for 30 min at 37 °C. Trypsinization was stopped by adding an equal volume of culture medium (Dulbecco’s modified Eagle’s medium-F-12 nutrient mixture, fetal bovine serum 10%, penicillin 100 U/mL, streptomycin 100 μg/mL and amphotericin B 0.5 μg/mL) with 160 μg/mL deoxyribonuclease I (all from Invitrogen or Sigma) and brought to a single cell suspension by repeated pipetting followed by passage through a 100-μm pore mesh. The solution was pelleted (7 min, 200 g) and resuspended in culture medium. Glial cells were seeded at a density of 3.5 × 10^5^ cells/mL and cultured at 37 °C in humidified 5% CO_2_–95% air. Medium was replaced once a week. Microglial cultures were prepared from DIV19-21 mixed glial cultures by the mild trypsinization method [12] and used 24 h after isolation.

### Ex vivo isolation of adult microglia

Microglial cells were acutely isolated from male adult mouse brains as described [13]. Briefly, mice were deeply anesthetized with isofluorane, perfused transcardially with ice-cold PBS + 10 U/ml heparin and the brains dissected and dissociated by trypsin digestion as described in the “Primary microglial cultures” section. Dissociated cells were then resuspended in 30% Percoll (GE Healthcare) and centrifuged for 10 min at 700*g*. The myelin-containing supernatant was removed, and the pelleted cells were washed twice with ice-cold PBS. Cells were then incubated with 10 μl CD11b Microbeads (Miltenyi Biotec) and 90 μl buffer (PBS supplemented with 0.5% BSA and 2 mM EDTA) for 15 min at 4 °C. After washing, CD11b+ cells were separated in a magnetic field using MS columns (Miltenyi Biotec). The CD11b+ fraction was collected and used for further analyses. The yield was 379.415 ± 79.073 (SD, *n* = 48) microglial cells per brain.

### In vivo systemic lipopolysaccharide (LPS) injection

Systemic LPS injection was used as an in vivo model of acute neuroinflammatory response. Eight-week-old male mice were injected i.p. with 4 mg/kg LPS (055:B5, Sigma-Aldrich; 100 μl per animal) or vehicle (PBS), and microglia was isolated 16 h after LPS injection as described above.

### Experimental autoimmune encephalomyelitis

EAE, an animal model of multiple sclerosis, was used to analyze the effect of microglial C/EBPβ deficiency in an in vivo model of a neurological disorder. LysMCre, C/EBPβ^fl/fl^ and LysMCre-C/EBPβ^fl/fl^ 7–8-week-old female mice were used. Under isofluorane anesthesia, the animals were subcutaneously injected at two sites into the flanks with 200 μl of a freshly prepared immunization cocktail containing myelin oligodendrocyte glycoprotein peptide (MOG35-55; Sigma; 100 μg/mouse), complete Freund’s adjuvant (Sigma) and *Mycobacterium tuberculosis* (H37R; Difco; 1 mg/mouse). Control mice received immunization cocktail without MOG35-55. Immediately after immunization and 2 days later, mice were injected i.p. with *Bordetella pertussis* toxin (Sigma, 500 ng/mouse). Mice were weighed and scored daily from day 8 post-immunization in a blind manner according to the following scale: 0, no deficit; 1, tail paralysis; 2, hind limb paresis; 3, incomplete hind limb paralysis; 4, complete hind limb paralysis; and 5, moribund state or death. Wet food was supplied when score 2 was reached and 200 μL of saline was administrated subcutaneously when animals scored 4. For comparison of EAE progression among genotypes, mice were scored until 52 days post-immunization at which point they were sacrificed. For analysis of C/EBPβ messenger RNA (mRNA) and protein expression in EAE, CFA- and MOG35-55-treated wild-type mice were sacrificed at various time-points post-immunization (9, 14, 21 and 28 days). The spinal cords were dissected into cervical, thoracic and lumbar regions, whereas the brains were dissected into hindbrain, midbrain and forebrain. Tissue samples were quickly frozen and stored at −80 °C.

### Immunocytochemistry

Cultured cells were fixed with 4% paraformaldehyde in PBS for 20 min at RT. After permeation with chilled methanol for 7 min and three PBS rinses, cells were incubated overnight at 4 °C with the primary antibody diluted in 7% normal goat serum (Sigma) in PBS containing 0.01% sodium azide. After rinsing in PBS, cells were incubated for 1 h at RT with secondary antibody and DAPI (5 μg/mL). The primary antibodies were monoclonal mouse anti-C/EBPβ (1:500, Abcam, ab-18336), polyclonal rabbit anti-nitric oxide synthase 2 (NOS2) (1:400, BD Transduction Laboratories, 610333), polyclonal rabbit anti-GFAP (1:1000, DakoCytomation, Z0334), polyclonal rabbit anti-Iba1 (1:1000, Wako, 019-19741) and monoclonal rat anti-CD11b (1:300, Serotec, MCA711G, clone 5C6). The secondary antibodies were goat anti-mouse Alexa 546 (1:1000, Molecular Probes, A-11018), Alexa 488 (1:1000, Molecular Probes, A11017), goat anti-rabbit, Alexa 488 (1:1000, Molecular Probes, A-11070) and goat anti-rat Alexa 546 (1:1000, Molecular Probes, A11081). Microscopy images were obtained with an Olympus IX70 microscope and a digital camera (CC-12, Soft Imaging System GmbH).

### Cytospin

25.000 microglial cells from one adult mouse brain isolated as described in the “Ex vivo isolation of adult microglia” section were centrifuged for 5 min at 1000 rpm at RT using Shandon Cytospin 4 (Thermo Scientific) and collected in gelatinized slides. Cells were then fixed with 4% paraformaldehyde in PBS for 20 min at RT and washed three times in PBS. After blocking for 30 min at RT with PBS containing 1% BSA, 0.03% Triton and 10% normal donkey serum (Gibco), cells were incubated overnight at 4 °C with primary antibodies diluted in blocking solution. After washing in PBS, slides were incubated for 1 h at RT with secondary antibodies and DAPI (5 μg/mL) diluted in blocking solution. The primary antibodies were monoclonal mouse anti-C/EBPβ (1:1111, AbCam, ab-18336) and monoclonal rat anti-CD68 (1:1000, Serotec, MCA1957). The secondary antibodies were donkey anti-mouse Alexa 546 (1:1000, Molecular Probes, A21203) and goat anti-rat Alexa 488 (1:1000, Molecular Probes, A21208). Microscopy images were taken with a Nikon Eclipse E 1000 microscope and a digital camera Olympus DP72.

### Histology

C/EBPβ^fl/fl^ and LysMCre-C/EBPβ^fl/fl^ 10-week-old female mice were deeply anesthetized with isofluorane and perfused transcardially with ice-cold 4% paraformaldehyde in PBS. The brain, lung, heart, mammary gland, spleen, liver, lung, kidney, femur bone and lumbar vertebrae were carefully dissected. Initial examination of macroscopic appearance of the organs was performed by reporting any gross anormality. Tissue specimens were formalin-fixed, paraffin-embedded using routine procedures and then cut into 5-μm semi-serial sections. Every fifth slide was stained with haematoxylin and eosin and examined under light microscope.

### NO determination

Primary microglial cultures from C/EBPβ^fl/fl^ and LysMCre-C/EBPβ^fl/fl^ mice were treated with LPS (100 ng/mL) with or without IFNγ (0.1, 1, 10 or 30 ng/mL) for 48 h. NO production was assessed by detecting accumulation of nitrites in the conditioned medium by the Griess colorimetric assay, as described [12].

### Isolation of total proteins and Western blot

Total microglial proteins were isolated by lysing pelleted cells from one 75 cm^2^ flask of primary microglial cultures per treatment condition (LPS 100 ng/mL with or without IFNγ 1 ng/mL for 24 h) and CD11b+ cells isolated from one adult brain as described in the “Ex vivo isolation of adult microglia” section with 50 μL of RIPA buffer (containing Igepal CA-630 (10 μL/mL), sodium deoxycholate (5 mg/mL), SDS (1 mg/mL) and protease inhibitor cocktail Complete®, in PBS. Protein amount was determined by the Bradford assay, and protein samples (20 μg) were denatured (5 min 95 °C), resolved by SDS-PAGE on 12% gels and transferred to a PDVF membrane. Membranes were incubated overnight at 4 °C with primary anti-C/EBPβ antibodies of mouse origin (Abcam, ab18336; for primary cultured microglia proteins) or rabbit origin (Abcam, ab32358; for acutely isolated adult microglia proteins) diluted in both cases 1:500 in immunoblot buffer (Tris-buffered saline (TBS) containing 0.05% Tween-20 and 5% nonfat dry milk). Following three TBS-0.05% Tween-20 rinses, HRP-labelled anti-mouse (1:2000, Santa Cruz, sc-2055) and anti-rabbit (1:15000, GE Healthcare, NA934) secondary antibodies were incubated for 45 min at RT. To label the loading control protein, monoclonal mouse anti-β-actin-peroxidase (1/40000, Sigma, A3854) was incubated for 20 min at RT. Proteins were visualized with WesternBright™ Quantum-ECL (Advansta) and membranes exposed to Agfa Curix RP2 Plus films. Data are expressed as the ratio between the intensity of the C/EBPβ band and the loading control protein band (β-actin). For Western blot with EAE and human postmortem samples, the same protocol was used with the exception that the primary antibody was co-incubated with an enhancer solution (SignalBoost™Immunoreaction Enhancer Kit, Merck).

### Total RNA extraction and qRT-PCR

Isolation of total RNA from microglial cell cultures was performed by lysing pelleted cells from one 75 cm^2^ flask per treatment condition (LPS 100 ng/mL with or without IFNγ 1 ng/mL for 6 h) with 1 mL of TriReagent (Sigma) and 100 μl of 1-bromo-3-chloropropane (BCP, Sigma). The aqueous phase containing total RNA was recovered after centrifugation for 15 min at 12.000*g* at 4 °C, mixed with an equal volume of ice-cold 70% ethanol and loaded onto a PureLink™ Micro Kit column (Invitrogen). Total RNA was then purified following manufacturer’s instructions. Isolation of total RNA from adult microglial cells was carried on after pelleting CD11b+ cells isolated from one adult brain as described in the “Ex vivo isolation of adult microglia” section, using PureLink™ Micro Kit (Invitrogen) following manufacturer’s instructions. Isolation of total RNA from spinal cord (cervical, thoracic and lumbar) and brain (mesencephalon plus diencephalon, rhombencephalon and telencephalon) samples of EAE mice was performed using Trizol method (Tri®Reagent, Sigma-Aldrich). Total RNA was quantified spectrophotometrically with Nano Drop ND-1000 (Thermo Scientific). Reverse transcription reactions were carried out from 300 ng (cultured cells) or 1 μg (tissue) of total RNA with random primers using Transcriptor Reverse Transcriptase (Roche). Complementary DNA (cDNA) was diluted 1/30, and 3 μl were used to perform qRT-PCR with qPCRBIO Sygreen Mix Lo-ROX (PCB-P20.11-50, Vitro) in 15 μl of final volume reaction using CFX96 Thermal Cycler equipment (Bio-Rad). Measurements were performed in duplicates. Primers, shown in Table 1, were used at final concentration of 300 nM. Samples were run for 45 cycles (95 °C for 30 s, 60 °C or 62 °C for 1 min and 72 °C for 30 s). Amplification specificity was confirmed by the analysis of melting curves, and relative gene expression values were calculated using Bio-Rad CFX Managing software (Bio-Rad) with the comparative Ct or ΔΔCt method.

**Table 1:** Primers used in this study

Gene	Forward	Reverse
Cebpb	AAG CTG AGC GAC GAG TAC AAG A	TCA GCT CCA GCA CCT TGT G
Il23a	TGT GCC CCG TAT CCA GTG TG	AAA AGC CAG ACC TTG GCG GA
Cybb	ACT CCT TGG GTC AGC ACT GGC	GCA ACA CGC ACT GGA ACC CCT
Tnfα	TGA TCC GCG ACG TGG AA	ACC GCC TGG AGT TCT GGA A
Ptges	AGG CCA GAT GAG GCT GCG GA	AGC GAA GGC GTG GGT TCA GC
Csf3	AGA GCT GCA GCC CAG ATC ACC	AGC TGC AGG GCC ATT AGC TTC A
Rn18s	GTA ACC CGT TGA ACC CCA TT	CCA TCC AAT CGG TAG TAG CG
Hprt1	ATC ATT ATG CCG AGG ATT TGG	GCA AAG AAC TTA TAG CCC CC
Sdha	TGG GGA GTG CCG TGG TGT CA	CAT GGC TGT GCC GTC CCC TG

### Viability

Microglial cell cultures from C/EBPβ^fl/fl^ and LysMCre-C/EBPβ^fl/fl^ mice were treated with LPS 1 μg/mL with or without IFNγ 1 or 30 ng/mL. Cells were fixed (0, 3, 5 or 7 days post-treatment) and probed as described in the “Immunocytochemistry” section. DAPI and Iba1-positive cells were manually counted using ImageJ software. In every experiment (*n* = 3), three wells per condition and three fields per well were analyzed. Results are represented as percentage of live cells on day 0.

### Phagocytosis

The *Salmonella enterica serovar Typhimurium* (*S. typhimurium*) SV5015 strain, a His+ derivative of the SL1344 strain (mouse-virulent) [14], was transformed with the pBR.RFP.1 plasmid [15] to render red autofluorescent bacterial cells. To test microglia phagocytic capacity, first, microglial cell cultures from C/EBPβ^fl/fl^ and LysMCre-C/EBPβ^fl/fl^ mice were treated with LPS 100 ng/mL with or without IFNγ 1 ng/mL in antibiotic-free culture medium. Twenty-four hours after treatment, microglia were infected with *S. typhimurium* for 30 min at multiplicity of infection of 5, defined as the ratio of bacteria per cultured cell. Non-ingested bacilli were eliminated by washing three times with PBS. Cells to assess phagocytic activity were then fixed as described in the “Immunocytochemistry” section, whereas to study resolution of infection, microglial cells were further incubated for 1 h in medium containing 100 μM gentamicin (Sigma-Aldrich) to kill extracellular bacteria and then switched to medium with a lower dose of gentamicin (10 μM) for 3 h before fixation. Cells were immunostained for Iba1 as described in the “Immunocytochemistry” section. Intracellular autofluorescent bacteria were manually counted using the ImageJ software along with DAPI and Iba1-positive cells. In every experiment (*n* = 3), two wells for each condition and three fields per well were analyzed. Results are represented as percentage of infected microglial cells and number of bacteria phagocytosed at 0.5 and 4 h post *Salmonella* infection.

### RNA sequencing (RNAseq)

Total RNA from primary microglia was isolated as described in the “Total RNA extraction and qRT-PCR” section. Total RNA integrity and quality were assessed with the Bioanalyzer 2100 system (Agilent). Library preparation and ultrasequencing were performed following Illumina’s (San Diego, CA) protocols. First, transfer RNA (tRNA) and ribosomal RNA (rRNA) were removed from 1 μg of total RNA using TruSeq Stranded Total RNA Sample Prep Kits (Illumina). Then, the RNA pool (mRNA + miRNA + lncRNA + other RNAs) was fragmented into pieces of approximately 200 bp using divalent cations under elevated temperature. The cleaved RNA fragments were reverse-transcribed into first-strand cDNA using reverse transcriptase and random primers. Next, the second strand was synthesized using DNA polymerase I and RNAse H. These double-stranded cDNA fragments were end-repaired by T4 DNA polymerase and Klenow DNA polymerase, and phosphorylated by T4 polynucleotide kinase. The cDNA products were incubated with Klenow DNA polymerase to generate 39 adenine overhangs, therefore allowing ligation to Illumina indexing adapters to the double-stranded cDNA ends. The adapter-ligated products were purified with Ampure XP magnetic beads (Agencourt Bioscience Corporation, Beverly, MA, USA), and libraries were amplified by 15 cycles of PCR with Phusion DNA polymerase (Finnzymes Reagents, Vantaa, Finland). Constructed libraries were validated and quantified using Bio-Rad’s automated electrophoresis system Experion and qRT-PCR, respectively. Pools of six indexed libraries were mixed (multiplexed) at equimolar ratios to yield a total oligonucleotide mix concentration of 10 nM. Finally, the resulting libraries were sequenced on the Genome Analyzer IIx platform (Illumina) to generate 150 bp single reads. Six pooled indexed libraries were sequenced in each flow cell lane. Raw sequence (FASTQ format) were processed through a series of sequential steps: (1) aggressive adapters removal; (2) alignment/mapping of RNA sequences to the mouse genome reference (Mus_musculus.mm10) using *tophat* software; and (3) sorting and cataloguing of the results by using *Samtools* software [16] in Bam files.

### RNAseq statistic and bioinformatic analyses

Bam files of RNA readings were processed using the Rsubread package [17] in the R environment and aligned to the mm10 version of mouse genome with the function *featureCounts* [18]. Summarized readings by gene were then normalized using *voom* normalization [19] to fit the count matrix into linear modelling with the package *limma* [20]. Normalized whole read counts were used to cluster samples with package *hcluster* using standard hierarchical cluster with average linkage. A linear fit model algorithm was used to obtain differentially expressed genes (DEGs) that complied with *p* < 0.01 and fold change >2. DEGs by comparison were summarized using the *VennDiagram* package. Heatmap visualization and clustering of DEGs was done with Genesis software after expressing gene values in standard deviation and using hierarchical clustering with average linkage by genes and samples. Finally, Genecodis [21] tools were used to obtain Gene Ontology and Kyoto Encyclopedia of Genes and Genomes (KEGG) pathway terms enrichment using the adjusted *p* value <0.01 after a hypergeometric test. Reads per kilobase per million mapped reads (RPKM) filtered values (sum of RPKM > 2 for each gene) were introduced to the Weighted Correlation Gene Network Analysis (WGCNA) package in R, to perform WGCNA as previously described [22]. Combined samples were surveyed and measured with the pickSoftThreshold function to obtain a correct power β considering the smallest value to give a free-scale topology. Using 9 as the power value, the blockwise function was used with parameters mergeCutHeight = 0.001 and detectCutHeight = 0.995 to obtain 34 modules codenamed by color, containing genes with high co-expression similarity. Module relationship with traits: treatment (Vehicle = 0; LPS = 1; LPS + IFNγ = 2) and genotype (C/EBPβ^fl/fl^ = 0; LysMCre-C/EBPβ^fl/fl^ = 1) was also calculated for each module to obtain a correlation. Gene symbols and normalized values were appended, and heatmap visualization of individual modules was obtained within the WGCNA package in R. Finally the Metacore™ platform was used to obtain the described interactions of proteins among the different modules to produce “external knowledge networks”, while VisAnt software was used with the correlation values in our experiments to produce the specific network of correlation within our data. For the separate analysis of DEGs in each experimental condition, samples of each group (control, LPS, LPS + IFNγ) were used in a separate pipeline, then a linear fit algorithm with Bayesian correction was applied grouping biological replicates of samples and designing a contrast matrix that compared genotypes selecting genes which complied with criteria fold change > 2, adjusted *p* < 0.05 (Benjamini–Hochberg procedure) as sample number was lower and power diminished for these analyses. DEGs in these lists were mostly in agreement with the whole comparison matrix DEGs.

### Statistics

Statistical analyses of RNAseq data are described in the “RNA sequencing (RNAseq)” section. For other parts of the study, statistical analyses were performed using one-way ANOVA followed by Newman–Keuls post hoc test when three or more experimental groups were compared. When the effect of treatment on genotype or the opposite were studied, two-way ANOVA followed by Bonferroni post-test was used. Non-parametric measures (EAE scores) were analyzed with Kruskal–Wallis test and Dunn’s multiple comparison post-test. Values of *p* < 0.05 were considered statistically significant. Results are represented as mean ± standard error of the mean (SEM). Experimental data were analyzed using GraphPad Prism 5.01 software.

## Results

### Characterization of LysMCre-C/EBPβ^fl/fl^ mice and their microglia in primary culture

**Fig. 1:**
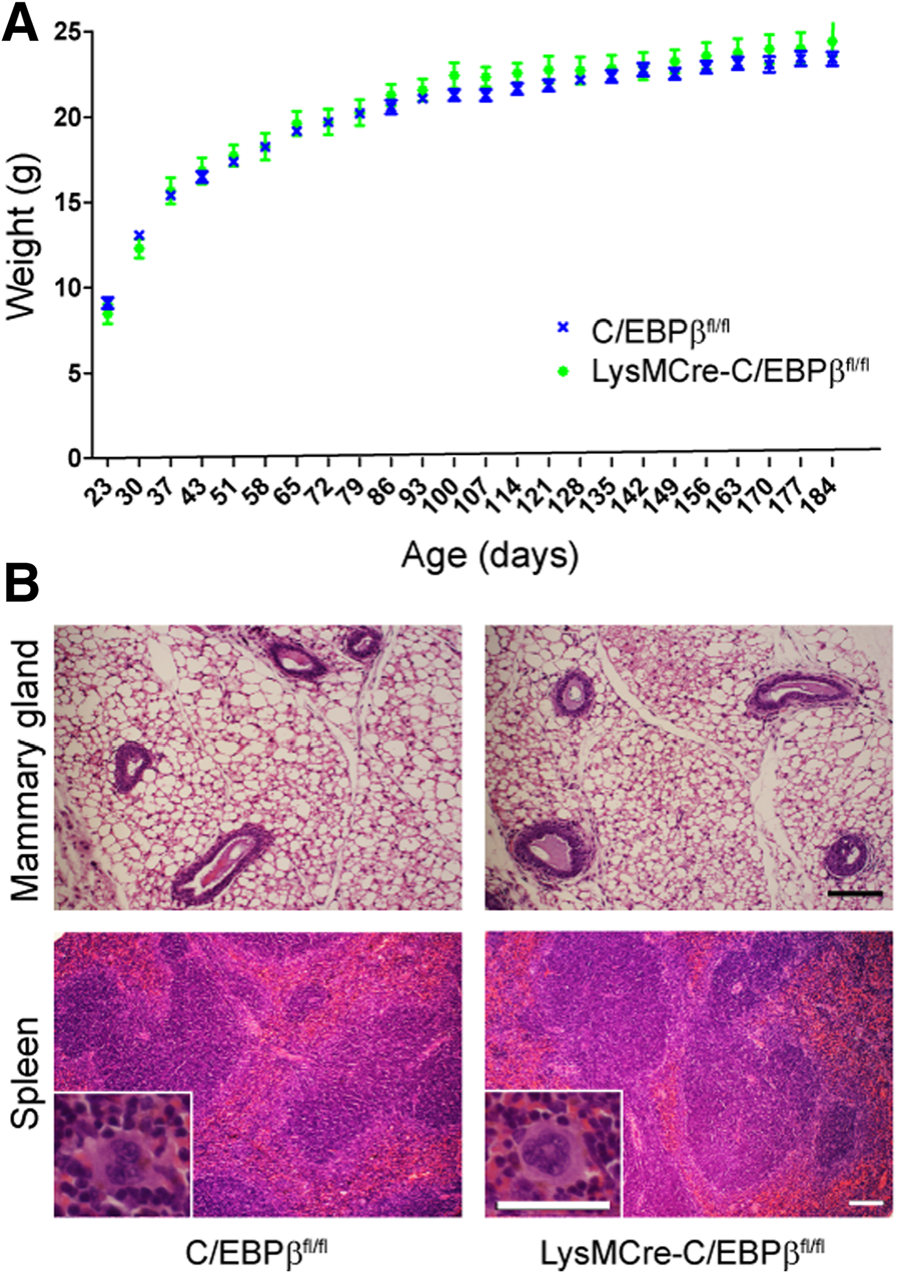
Phenotypic characterization of LysMCre-C/EBPβ^fl/fl^ mice. **a** Body weight curves of female C/EBPβ^fl/fl^ (*n* = 10) and LysMCre-C/EBPβ^fl/fl^ (*n* = 6) mice shown as mean ± SEM. No significant differences in body weight are observed between both genotypes. **b** Representative microscopic images of haematoxylin and eosin staining of the mammary gland and spleen from 10-week-old C/EBPβ^fl/fl^ and LysMCre-C/EBPβ^fl/fl^ mice. Normal ductal development and branching as well as unaltered adipose tissue in mammary gland from both genotypes are noted. Normal morphology of red and white pulps and megakaryocytes (*insets*) are also seen in both genotypes. No histological differences are observed also in the brain, lung, heart, liver, lung, kidney, femur bone and lumbar vertebrae (not shown). *Scale bars* 100 μm (*large images*) and 50 μm (*insets*)

LysMCre-C/EBPβ^fl/fl^ mice had normal appearance and body weight (Fig. 1a) and showed no obvious behavioral abnormalities when compared to wild type, LysMCre or C/EBPβ^fl/fl^ mice. Their survival rate and fertility were normal in contrast to what has been reported in C/EBPβ knockout mice [23]. Litter size and gender distribution were also normal. Histological evaluation of haematoxylin and eosin stained sections (Fig. 1b) showed normal mammary gland and red and white spleen pulp morphology, again in contrast to reports in mice with ubiquitous C/EBPβ deficiency [23]. No differences between genotypes were observed in all other tissues and organs examined, listed in the online methods section.

**Fig. 2:**
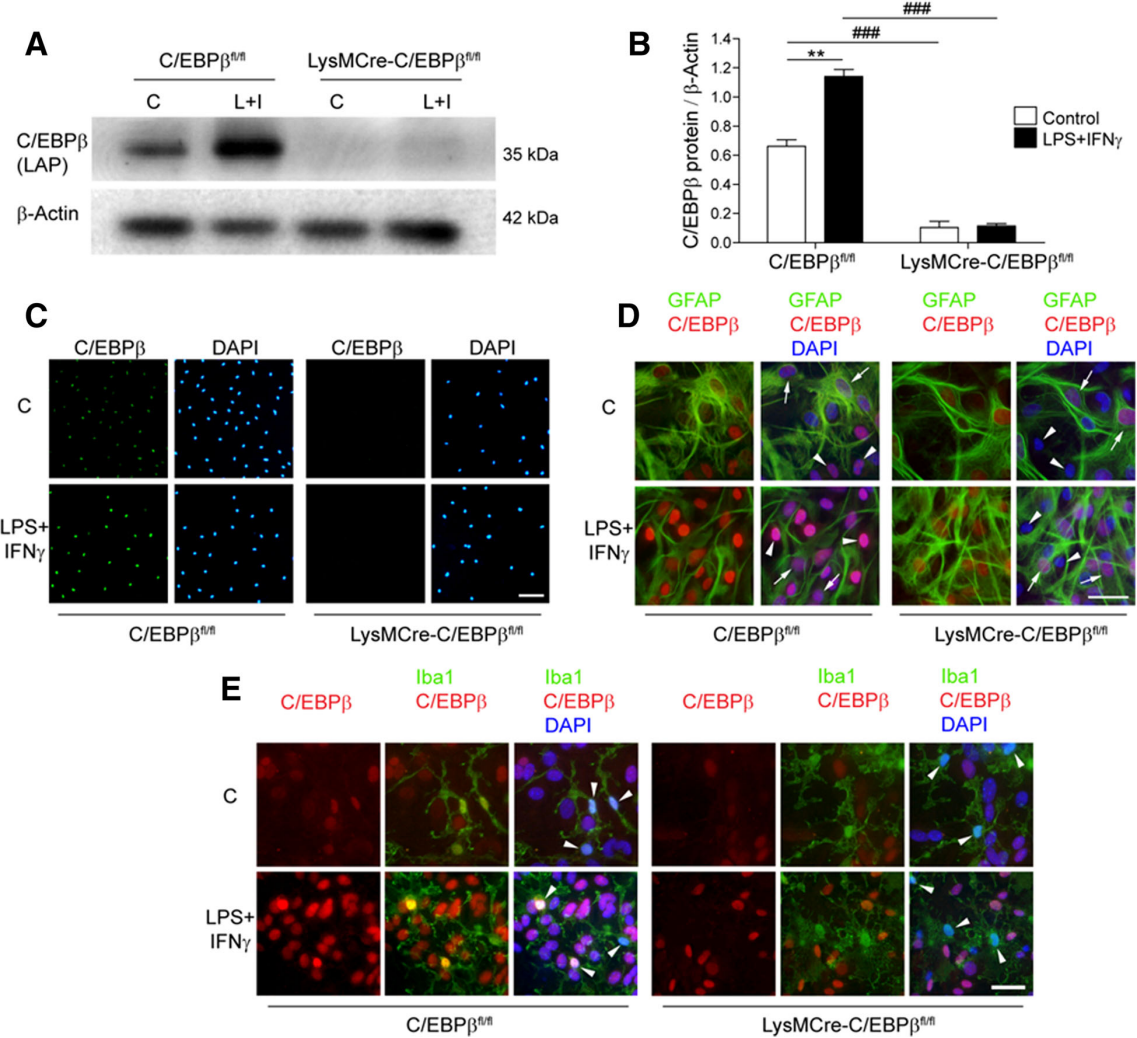
Reduced C/EBPβ protein in LysMCre-C/EBPβ^fl/fl^ microglia in culture. **a** Representative Western blot showing C/EBPβ LAP protein levels in primary microglial cultures from C/EBPβ^fl/fl^ and LysMCre-C/EBPβ^fl/fl^ mice treated with vehicle (C) or LPS (100 ng/mL) + IFNγ (1 ng/mL) (L + I) for 24 h. Detection of the C/EBPβ isoforms Full and LIP in these samples required longer exposure. **b** Quantification of Western blot signals from four independent experiments as that shown in **a**. C/EBPβ protein levels are normalized using β-actin levels. Data are shown as mean + SEM. Significant effect of treatment is observed in C/EBPβ^fl/fl^ microglia (**p < 0.01), and significant effect of genotype is observed both in vehicle- and LPS + IFNγ-treated microglia (###*p* < 0.001). **c** Primary microglial cultures were treated as in **a**, fixed and C/EBPβ protein was analyzed by immunocytochemistry and nuclei were counterstained with DAPI. Note the nuclear C/EBPβ expression in virtually all microglial cells in C/EBPβ^fl/fl^ cultures which is enhanced by LPS + IFNγ and the complete absence of C/EBPβ immunoreactivity in LysMCre-C/EBPβ^fl/fl^ microglial cells in culture. *Scale bar* 100 μm. **d**, **e** Primary mixed glial cultures containing mainly astrocytes and microglia were treated as in **a**, fixed, immunostained for C/EBPβ (*red*) and the astroglial marker GFAP (*green* in **d**) or microglial marker Iba1 (*green* in **e**) and counterstained with DAPI. In C/EBPβ^fl/fl^ cultures, C/EBPβ immunostaining is observed in both GFAP-positive cells (astrocytes; *arrows*) and Iba1-positive cells (microglia; *arrowheads*), whereas in LysMCre-C/EBPβ^fl/fl^ cultures, C/EBPβ is only observed in astrocytes. *Scale bar* 50 μm

Western blot analysis of proteins from primary microglial cultures showed robust C/EBPβ expression in C/EBPβ^fl/fl^ microglia, and this expression was enhanced by LPS + IFNγ treatment (Fig. 2a). Interestingly, C/EBPβ proteins were not detected in primary microglia from LysMCre-C/EBPβ^fl/fl^ mice, neither in control nor after LPS + IFNγ treatment (Fig. 2a, b). Immunocytochemical C/EBPβ staining confirmed this finding. In control conditions, C/EBPβ was present in most microglial cells in primary cultures from C/EBPβ^fl/fl^ mice, and the number of positive cells and the intensity of the staining was enhanced by LPS + IFNγ treatment (24 h). In contrast, microglia from LysMCre-C/EBPβ^fl/fl^ mice were devoid of C/EBPβ immunostaining, both in control and LPS + IFNγ conditions (Fig. 2c). These findings show that the efficiency of LysMCre-induced recombination of C/EBPβ LoxP sites in primary microglia from LysMCre-C/EBPβ^fl/fl^ mice is near 100%. C/EBPβ immunocytochemistry of mixed glial cultures confirmed the absence of C/EBPβ in LysMCre-C/EBPβ^fl/fl^ microglia, identified by Iba1 immunostaining (Fig. 2e), and showed the presence of normal levels of C/EBPβ immunostaining in astrocytes, identified by GFAP (Fig. 2d), thus supporting the specificity of LysMCre-induced recombination in microglia.

**Fig. 3:**
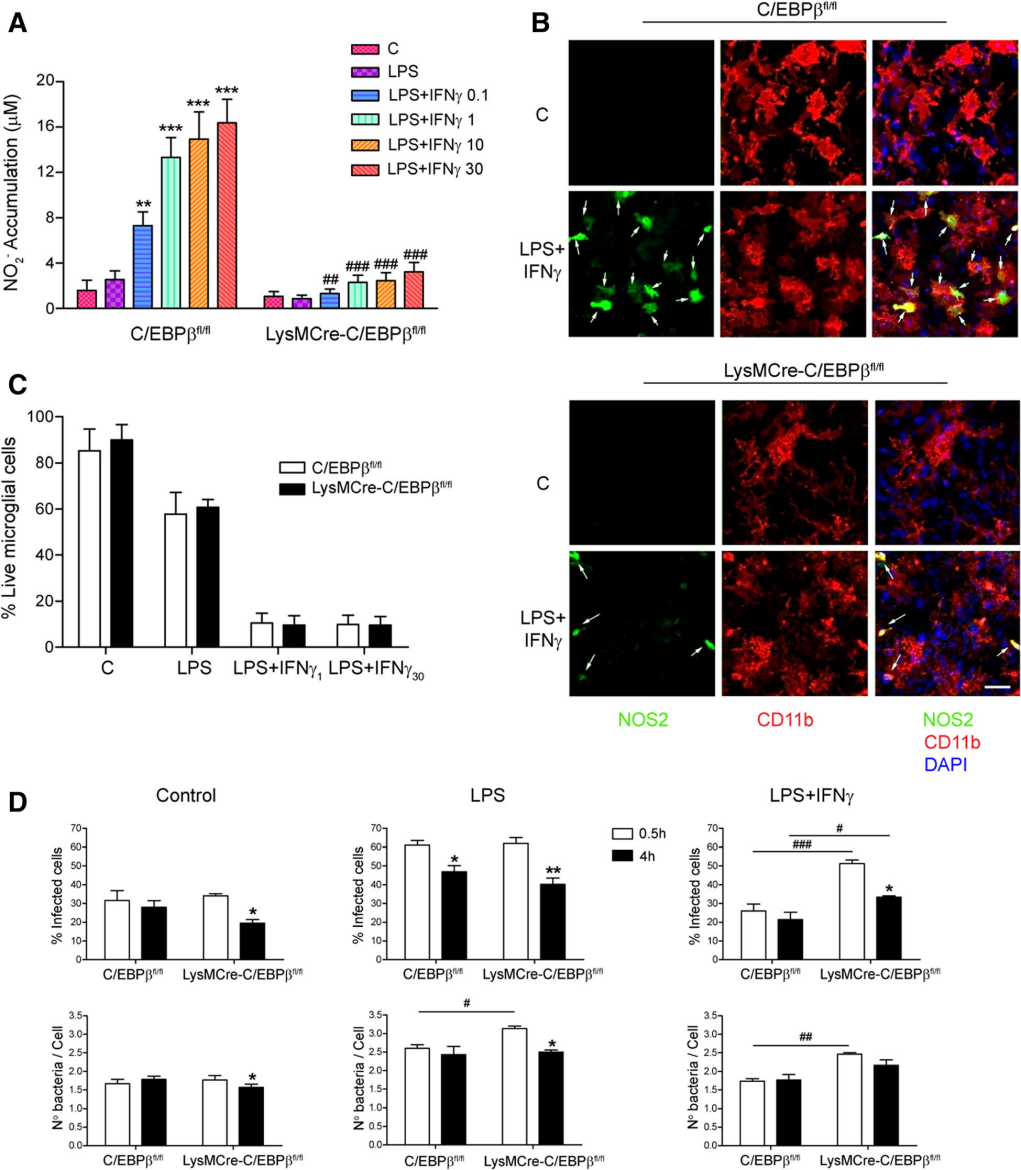
LysMCre-C/EBPβ^fl/fl^ microglial function in culture. **a** Primary microglial cultures from C/EBPβ^fl/fl^ and LysMCre-C/EBPβ^fl/fl^ mice were treated with vehicle (C), LPS (100 ng/mL) ± IFNγ (0.1, 1, 10, 30 ng/mL) for 48 h. NO production was estimated by measuring nitrites in the conditioned medium by the Griess reaction. Data show mean + SEM of six independent experiments. *Asterisks* show the significance of the treatment effect (**p < 0.01; ***p < 0.001 compared with the respective control conditions) and *number sign* shows the significance of the genotype effect (##*p* < 0.01; ###*p* < 0.001 compared with the respective C/EBPβ^fl/fl^ condition). **b** Mixed glial cultures from C/EBPβ^fl/fl^ and LysMCre-C/EBPβ^fl/fl^ mice were treated for 24 h with vehicle (C) or LPS (100 ng/mL) + IFNγ (1 ng/mL) and processed for immunocytochemistry for NOS2 (*green*), the microglial marker CD11b (*red*) and the nuclear counterstain DAPI (*blue*). LPS-induced NOS2 expression in C/EBPβ^fl/fl^ cultures is markedly attenuated in LysMCre-C/EBPβ^fl/fl^ cultures. NOS2 expression is always microglial as revealed by CD11b colocalization. *Arrows* show NOS2-positive cells. *Scale bar*, 100 μm. **c** Primary glial cultures from C/EBPβ^fl/fl^ and LysMCre-C/EBPβ^fl/fl^ mice were treated with vehicle (C), LPS (100 ng/mL) ± IFNγ (1 and 30 ng/mL). Five days after treatment, Iba1-positive microglial cells were counted as indicated in the “Methods” section. Data represent the percentage of microglial cells on day 0 shown as mean + SEM of three independent experiments. Note the marked AICD effect induced by LPS and particularly by LPS + IFNγ and the absence of differences between the two genotypes. **d** Primary microglial cell cultures from C/EBPβ^fl/fl^ and LysMCre C/EBPβ^fl/fl^ mice were treated with vehicle (C), LPS (100 ng/mL) or LPS + IFNγ (1 ng/mL) for 24 h prior to infection with fluorescent *Salmonella* for 30 min. Microglial cultures were fixed immediately (*white bars*) or 4 h later (*black bars*), and the percentage of infected microglial cells and the number of bacteria phagocytosed were analyzed. Data show mean + SEM of three independent experiments. The *asterisk* shows the significance of the differences between 30 min and 4 h (*p < 0.05; **p < 0.01 compared with the respective 30-min value) and the *number sign* shows the significance of the genotype effect (#*p* < 0.05; ##*p* < 0.01; ###*p* < 0.001 compared with the respective C/EBPβ^fl/fl^ condition)

We were next interested in determining whether the absence of C/EBPβ affects microglial function. To this end, we assessed NO production and NOS2 expression, activation-induced cell death (AICD) and phagocytosis of bacterial cells by primary microglial cultures from C/EBPβ^fl/fl^ and LysMCre-C/EBPβ^fl/fl^ mice. NO production is a key element in pro-inflammatory microglial activation and others, and we showed its regulation by C/EBPβ in microglia [3, 24]. In primary microglial cultures from C/EBPβ^fl/fl^ mice, co-treatment of LPS with IFNγ induced a dose-dependent increase in NO production which was markedly attenuated in LysMCre-C/EBPβ^fl/fl^ microglia (Fig. 3a). We then assessed by immunocytochemistry the expression of NOS2, the NO producing enzyme in activated microglia, in mixed glial cultures from both genotypes in control and LPS + IFNγ conditions. NOS2 was undetectable in control conditions, but it was induced by LPS + IFNγ in C/EBPβ^fl/fl^ glial cultures, and this response was markedly attenuated in LysMCre-C/EBPβ^fl/fl^ cultures (Fig. 3b). CD11b immunostaining revealed the microglial nature of NOS2-expressing cells in activated mixed glial cultures. In this experiment, we could not use Iba1 as microglial marker because both anti-Iba1 and anti-NOS2 antibodies were from a rabbit. These findings provide a first indication of an attenuated pro-inflammatory response in microglia from LysMCre-C/EBPβ^fl/fl^ mice.

Overactivation of microglia leads to apoptotic cell death in a process named AICD thought to contribute to maintaining low numbers of activated microglia after brain damage [25]. The role of C/EBPβ in microglial AICD has not been studied, but since C/EBPβ regulates microglial NO production and NO is an important AICD effector in microglia, we hypothesized that AICD could be attenuated in C/EBPβ-deficient microglia. In C/EBPβ^fl/fl^ microglia, LPS induced a clear AICD effect (40% cell death, 5 days) that was markedly potentiated by IFNγ (90% cell death, 5 days) (Fig. 3c). The same effect was observed in LysMCre-C/EBPβ^fl/fl^ microglia thus ruling out a key role for C/EBPβ in microglial AICD. Similar results were obtained 3 and 7 days after treatments (data not shown).

We were finally interested to study the role of C/EBPβ in phagocytosis, one of the major physiological roles of microglia. To this end, control and activated microglia from both genotypes were incubated with autofluorescent *S. typhimurium*, a Gram-negative bacterium. The percentage of infected microglia and the number of bacteria per microglial cell were assessed 30 min and 4 h after addition of bacteria. Data obtained in 30 min indicate phagocytic capacity whereas data in 4 h show the ability of microglia to eliminate phagocytosed bacterial cells. As shown in Fig. 3d, 30 min after bacteria addition, no differences in phagocytosis between both genotypes were observed in untreated microglia. However, after LPS + IFNγ treatment, LysMCre-C/EBPβ^fl/fl^ microglia showed higher number of infected cells and higher intake of bacteria per cell than C/EBPβ^fl/fl^ microglia, whereas after LPS treatment, LysMCre-C/EBPβ^fl/fl^ microglia showed higher number of phagocytosed bacteria per cell. Four hours after bacteria addition, differences between both genotypes were attenuated. Only in LysMCre-C/EBPβ^fl/fl^ microglia treated with LPS + IFNγ there was an increase in the percentage of infected cells, but of lower magnitude than that observed in 30 min. In C/EBPβ^fl/fl^ microglia, the percentage of infected cells and the number of bacteria/cell in 4 h were not decreased when compared with the 30 min values, with only one exception (% infected cells after LPS treatment). In contrast, in LysMCre-C/EBPβ^fl/fl^ microglia, the percentage of infected cells and the number of bacteria/cell in 4 h were decreased vs 30 min (with one exception: bacteria/cell after LPS + IFNγ treatment). These data demonstrate increased phagocytic activity and higher capacity to eliminate phagocytosed bacteria of C/EBPβ-deficient microglia.

### C/EBPβ-deficient cultured microglia show marked changes of gene expression

After demonstrating the widespread deficiency of C/EBPβ in microglia from LysMCre-C/EBPβ^fl/fl^ mice and its functional effects, we assessed the global effects of C/EBPβ deficiency in microglial gene expression by comparing by RNAseq the transcriptomic profiles of C/EBPβ^fl/fl^ and LysMCre-C/EBPβ^fl/fl^ primary microglial cultures treated with vehicle, LPS or LPS + IFNγ for 6 h.

**Fig. 4:**
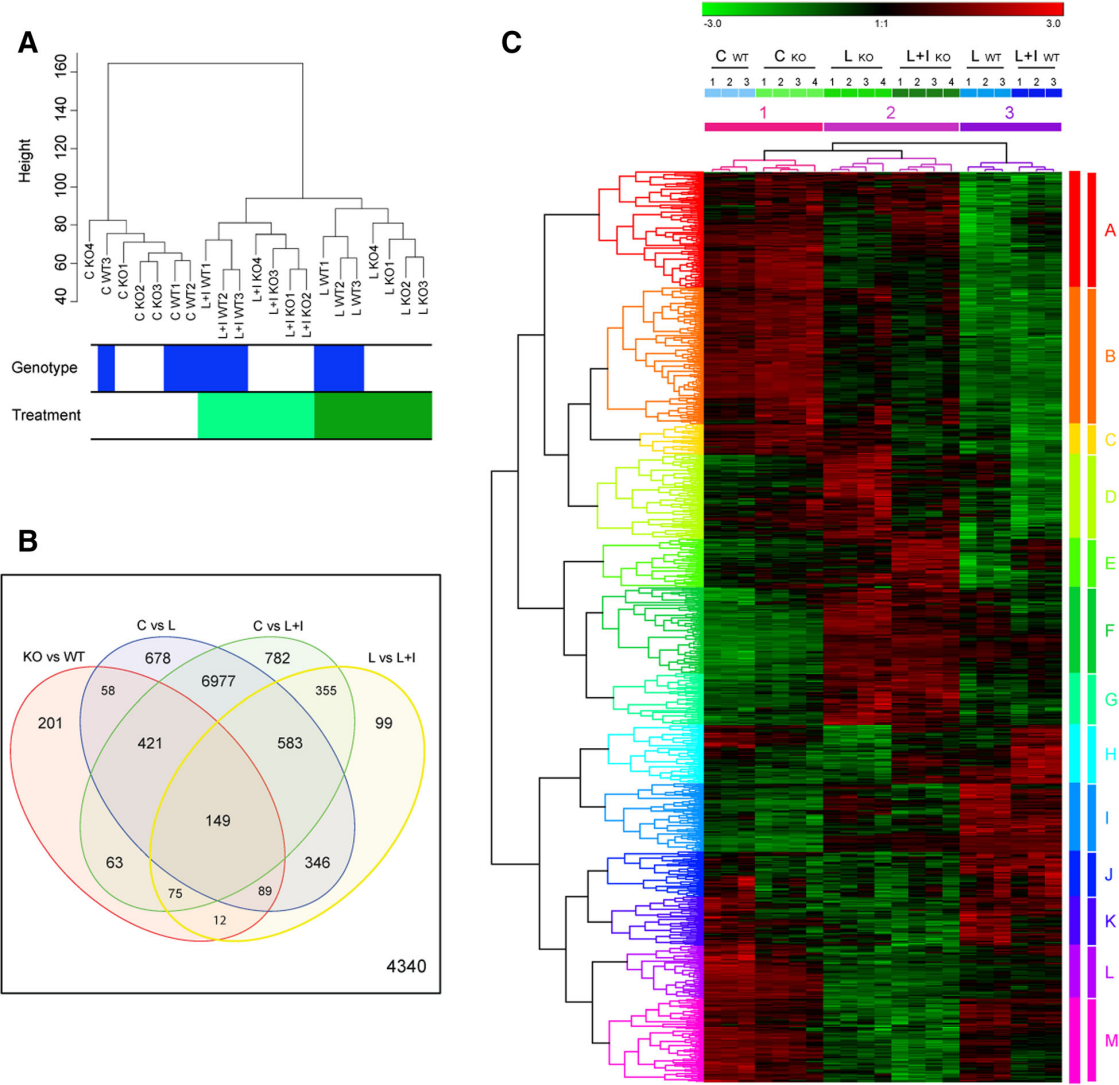
Treatment with LPS ± IFNγ (6 h) evidences differential transcriptomic changes between C/EBPβ^fl/fl^ and LysMCre-C/EBPβ^fl/fl^ microglia. **a** Hierarchical clustering of total RNASeq RPKM normalized values separate samples neatly by treatment. Remarkably, C/EBPβ^fl/fl^ and LysMCre C/EBPβ^fl/fl^ groups are mixed in the control condition, but separate into subgroups once any treatment is applied to microglia (Euclidean distance). **b** Linear model fitting of filtered genes (RPKM sum of genes through samples >2) detects 10,888 genes as differentially expressed (DEGs) (lfc > 1, adjusted *p* value <0.01 Benjamini–Hochberg correction). Contrasts between groups are shown on the Venn diagram. More specifically, 1068 genes are detected as DEGs for the C/EBPβ^fl/fl^ vs LysMCre-C/EBPβ^fl/fl^ contrast. **c** Heatmap of C/EBPβ^fl/fl^ and LysMCre-C/EBPβ^fl/fl^ DEGs (*n* = 1068) with applied hierarchical clustering (average linkage, Euclidean distance). Values are represented as SD from the normalized average for each gene. Sample clustering evidences three main groups; with cluster 1 (*pink*) corresponding to the control condition which contain subclusters separating C/EBPβ^fl/fl^ and LysMCre-C/EBPβ^fl/fl^. Remarkably, clusters 2 (*magenta*) and 3 (*purple*) correspond to C/EBPβ^fl/fl^ and LysMCre-C/EBPβ^fl/fl^ conditions and contain subclusters organized by treatment. On the other hand, gene cluster classification (A–M) evidences the combination of patterns of expression among samples. In this figure, WT and KO refer to C/EBPβ^fl/fl^ and LysMCre-C/EBPβ^fl/fl^, respectively

RNA libraries and sequencing where prepared as described in the “Methods” section. Unsupervised cluster analysis using normalized counts (RPKM) showed a strong effect of treatments on microglial RNA expression. The 21 samples were grouped in three clusters corresponding without exception to microglial cultures treated with vehicle (*n* = 7), LPS (*n* = 7) and LPS + IFNγ (*n* = 7) (Fig. 4a). The clustering analysis also revealed a marked effect of genotype on RNA expression, particularly in activated microglia. Thus, LPS- and LPS + IFNγ-treated samples were subclassified into two clusters corresponding to genotypes C/EBPβ^fl/fl^ and LysMCre-C/EBPβ^fl/fl^ microglia (Fig. 4a). This analysis strongly suggests that C/EBPβ plays an important role in the regulation of gene expression in activated microglia. Afterwards, the validated pipeline for differentially expressed genes (DEGs) detection Rsubread/voom normalization/limma [20] was used selecting genes that complied with fold change >2 and *p* < 0.01 (Benjamini–Hochberg method for multiple comparisons) in pairs of conditions compared. Out of 15228 genes with significant RNA expression in microglia, 1068 were affected by C/EBPβ deficiency. As the Venn diagram in Fig. 4b shows, most of these were genes also significantly affected by LPS (717 genes) or LPS + IFNγ (708 genes) and only 201 of C/EBPβ-dependent genes were unaffected by LPS or LPS + IFNγ again indicating a major role for C/EBPβ on gene expression in activated microglia. In addition, we separately analyzed the samples to show the DEG between C/EBPβ^fl/fl^ and LysMCre-C/EBPβ^fl/fl^ microglia in each treatment condition (Control, LPS, LPS + IFNγ). In control conditions C/EBPβ absence resulted in more genes being downregulated (44 genes) than upregulated (13 genes); in the LPS condition the opposite was observed (459 upregulated and 339 downregulated); and in the LPS + IFNγ condition the number of upregulated and downregulated genes was similar (197 and 217 genes, respectively) (Additional file 1: Tables S1–S6).

DEGs log2 values of C/EBPβ^fl/fl^ and LysMCre-C/EBPβ^fl/fl^ comparison (*n* = 1068) were normalized to be expressed in standard deviation values (±3 SD) and represented on a heatmap (Fig. 4c). A hierarchical cluster algorithm for samples and genes revealed two sample clusters: cluster 1 which included untreated samples of both genotypes and treated LysMCre-C/EBPβ^fl/fl^ samples and cluster 2 which included treated C/EBPβ^fl/fl^ samples. This shows that LPS ± IFNγ-treated LysMCre-C/EBPβ^fl/fl^ microglia have an attenuated phenotype with a gene expression profile closer to untreated microglia than to treated C/EBPβ^fl/fl^ microglia. This analysis also shows a neat separation of untreated samples into two clusters corresponding to the two genotypes demonstrating in an unbiased way the altered biology of C/EBPβ-deficient microglia in basal conditions. Additionally, a Weighted Correlation Gene Network Analysis (WGCNA) was performed, separating all 15,228 genes into 35 modules. The dendrogram of WGCNA revealed a large grouping of genes corresponding to module “turquoise” which represent genes with decreased expression upon LPS or LPS + IFNγ treatments (Additional file 2: Figure S1A). Complementarily, dendrogram clustering of modules highlighted module “violet” as the closest group of genes correlating with genotype differences (Additional file 2: Figure S1B). Module violet contained C/EBPβ as expected (Additional file 2: Figure S1C) and additionally a set of genes linked by outer literature databases (Metacore™; Additional file 2: Figure S1D) and within the distance measures of experiment data (Additional file 2: Figure S1E).

**Fig. 5:**
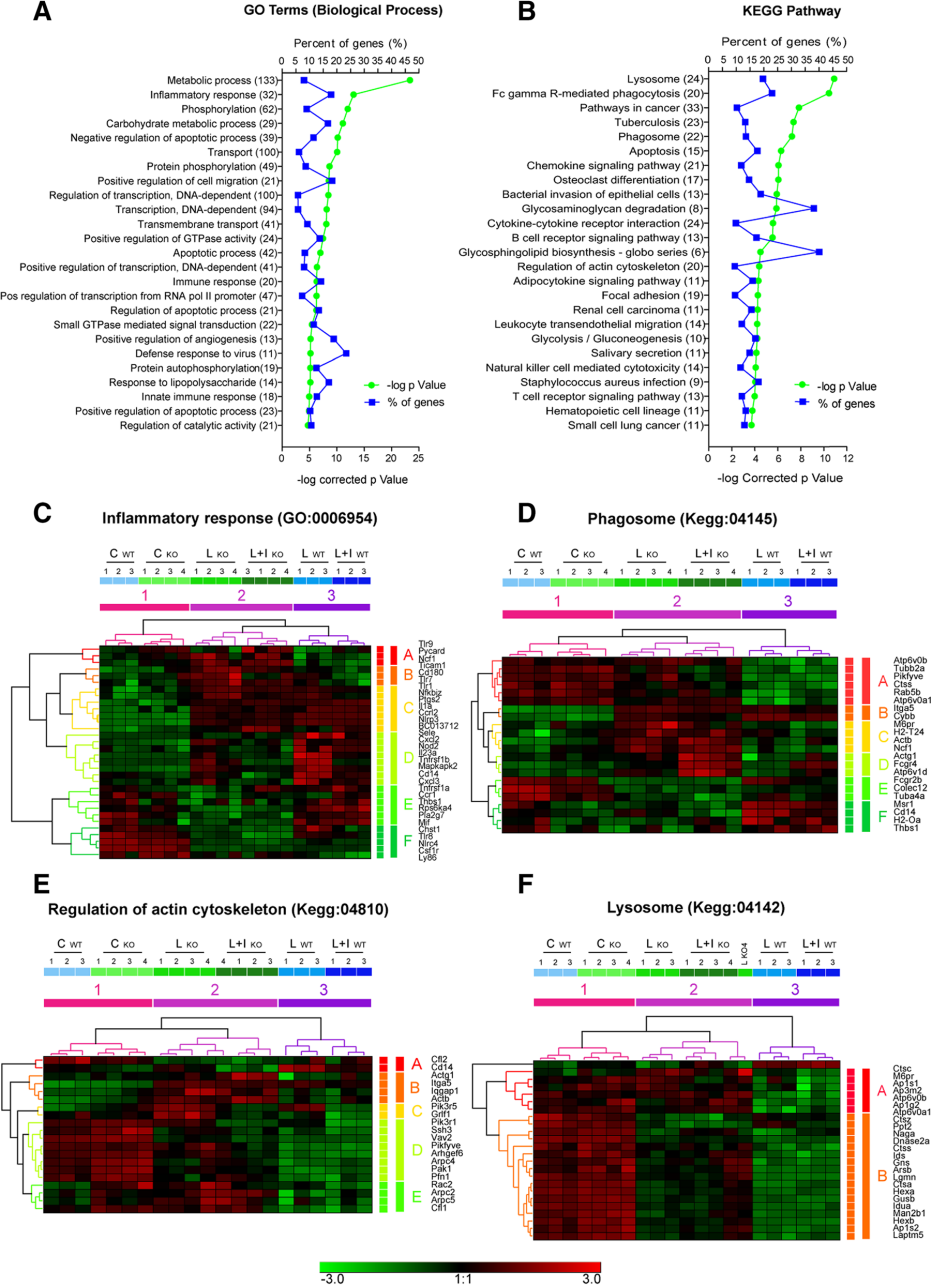
Ontology enrichment analyses reveal biological implication of obtained DEGs with microglial processes. C/EBPβ^fl/fl^ and LysMCre-C/EBPβ^fl/fl^ DEGs where fed into the Genecodis platform for ontology enrichment. **a** *p* value and gene percentage annotation graph of the biological process (BP) category from Gene Ontology database (hypergeometric distribution). **b** *p* value and gene percentage annotation graph from Kyoto Encyclopedia of Genes and Genomes (KEGG) pathway enrichment analyses (hypergeometric distribution). **c**–**e** Heatmap of genes annotated with the enriched categories shown above (hierarchical clustering, average linkage, Euclidean distance, scale in SD over normalized gene expression in log2 values). Note the existence of gene clusters with distinct patterns of gene expression changes, e.g. cluster B in **f** corresponds to lysosome-related genes with reduced expression upon LPS ± IFNγ treatments, in C/EBPβ^fl/fl^ microglia that is attenuated in LysMCre-C/EBPβ^fl/fl^ microglia. In this figure, WT and KO refer to C/EBPβ^fl/fl^ and LysMCre-C/EBPβ^fl/fl^, respectively

Finally, we used the Genecodis platform [21] to obtain DEG enrichment in different annotation databases, specifically for Gene Ontology (GO) terms for Biological Process (Fig. 5a), and for the Kyoto Encyclopedia of Genes and Genomes (KEGG) pathways (Fig. 5b). Notably, various GO categories and KEGG pathways related to inflammatory and innate immune responses were among the most significantly represented (e.g. inflammatory response, GO:0006954; Fig. 5c). Interestingly, lysosome and phagosome KEGG pathways were also particularly represented (Fig. 5d–f).

In summary, this analysis shows the importance of microglial C/EBPβ, since its deletion affects the expression of 1068 genes. It confirms the involvement of C/EBPβ in the regulation of pro-inflammatory programs in activated microglia, but it also reveals a role of C/EBPβ in other programs such as phagocytosis and apoptosis. Interestingly, it reveals for the first time a role for C/EBPβ in non-stimulated microglia.

### Robust C/EBPβ deletion in LysMCre-C/EBPβ^fl/fl^ microglia in vivo and subsequent attenuated pro-inflammatory gene expression

**Fig. 6:**
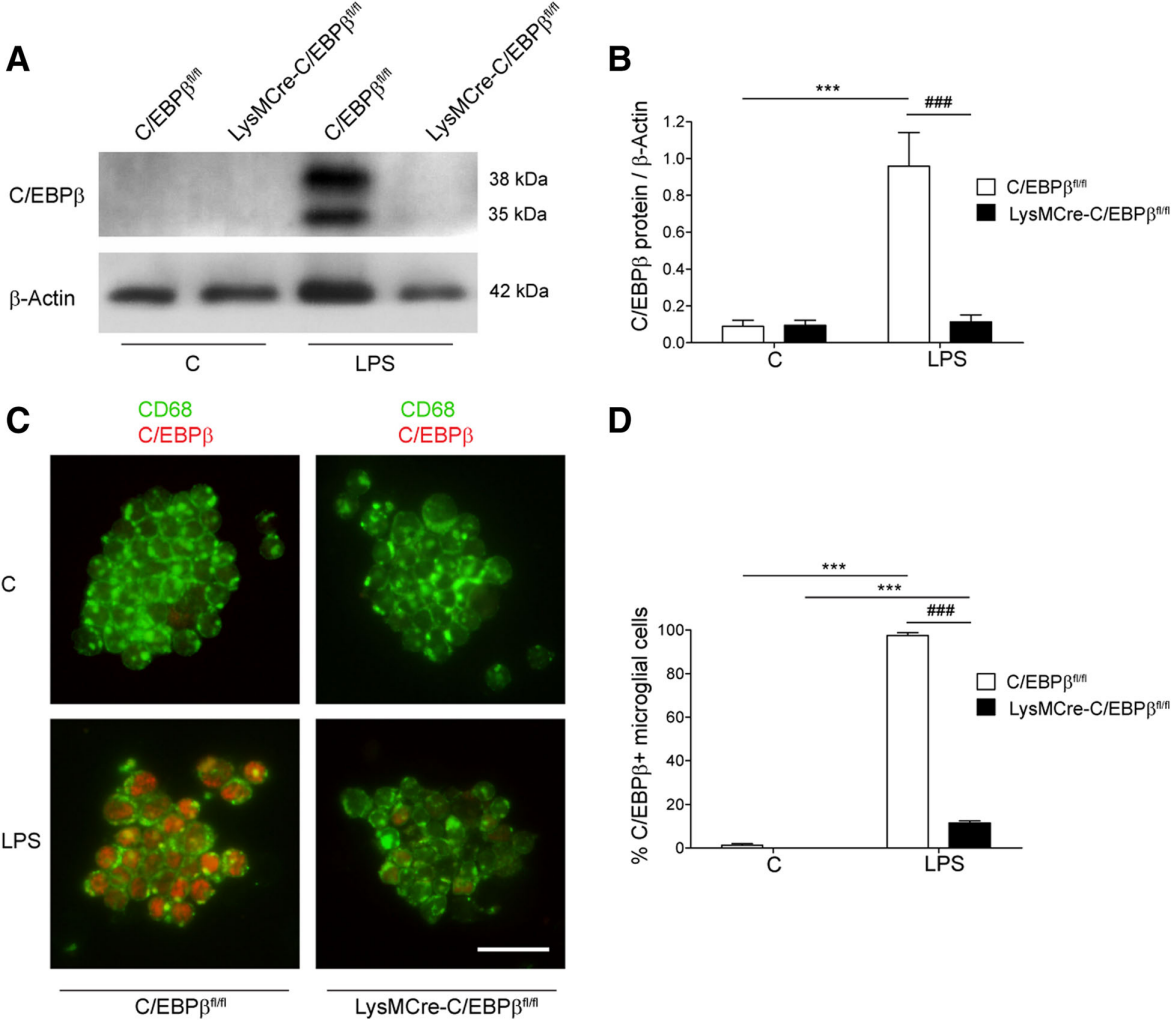
Reduced C/EBPβ expression in LysMCre-C/EBPβ^fl/fl^ microglia in vivo. **a** Representative Western blot showing C/EBPβ protein levels in microglia acutely isolated in vivo from the brains of C/EBPβ^fl/fl^ and LysMCre-C/EBPβ^fl/fl^ mice treated i.p. with vehicle (C) or LPS (4 mg/kg) for 16 h. Two C/EBPβ isoforms are detected, Full (~38 kDa) and LAP (~35 kDa). **b** Quantification of Western blot signals from experiments as that shown in **a** (*n* = 4 mice/condition. C/EBPβ protein levels are normalized using β-actin levels. Data are shown as mean + SEM. LPS induces a significant increase in C/EBPβ protein levels in C/EBPβ^fl/fl^ microglia (***p < 0.001, compared with respective vehicle) and not in LysMCre-C/EBPβ^fl/fl^ microglia (###*p* < .001, compared with C/EBPβ^fl/fl^). **c** Immunocytochemistry for C/EBPβ (*red*) and the microglial marker CD68 (*green*) in cytospin preparations of microglia isolated from the brains of C/EBPβ^fl/fl^ and LysMCre-C/EBPβ^fl/fl^ mice treated as in **a**. Note the absence of C/EBPβ immunoreactivity in microglia from vehicle-treated mice, the nuclear C/EBPβ staining in virtually all microglial cells from LPS-treated C/EBPβ^fl/fl^ mice and the presence of C/EBPβ in a small fraction of microglial cells from LPS-treated LysMCre-C/EBPβ^fl/fl^ mice. *Scale bar* 25 μm. **d** Quantification of the proportion of C/EBPβ immunoreactive microglial cells in cytospin preparations as that shown in **c**. Data are expressed as percentage of C/EBPβ immunoreactive microglial cells and are shown as mean + SEM (*n* = 3 mice/condition). ***p < 0.001, compared with respective vehicle; ###*p* < .001, compared with respective C/EBPβ^fl/fl^

We were next interested to study the consequences of microglial C/EBPβ deletion in vivo. A first step was to estimate the efficiency of LysMCre-induced recombination of C/EBPβ LoxP sites in microglial cells in vivo. To this end, microglia was acutely isolated by immunomagnetic separation from the whole brain of C/EBPβ^fl/fl^ and LysMCre-C/EBPβ^fl/fl^ adult mice treated systemically with LPS or vehicle for 16 h. At the dose and time frame used, systemic LPS induces a neuroinflammatory response with increased C/EBPβ expression in the CNS [26]. C/EBPβ protein was not detected by Western blot in microglia protein extracts from vehicle-treated mice of both genotypes. Systemic LPS induced the strong expression of C/EBPβ Full and LAP protein isoforms in C/EBPβ^fl/fl^ microglia, and this induction was completely ablated in microglia isolated from LPS-treated LysMCre-C/EBPβ^fl/fl^ mice brains (Fig. 6a, b). These findings were confirmed and extended by immunocytochemistry for C/EBPβ in cytospin microglial preparations. Immunostaining for the microglial markers CD68 (Fig. 6c) and Iba1 (not shown) confirmed the microglial nature of the ex vivo isolated cells. As shown in Fig. 6c, d, C/EBPβ immunoreactivity was not observed in microglia from vehicle-treated mice of both genotypes, whereas it was present in virtually 100% of microglial cells from LPS-treated C/EBPβ^fl/fl^ mice. In these cells, C/EBPβ showed nuclear localization. In contrast, in LPS-treated LysMCre-C/EBPβ^fl/fl^ microglial cytospin preparations, C/EBPβ immunoreactivity was only observed in 11 ± 1% of microglial cells (Fig. 6c, d). These findings demonstrate a robust C/EBPβ deletion in LysMCre-C/EBPβ^fl/fl^ microglial cells not only in vitro, where the efficiency of recombination approaches 100%, but also in vivo where this efficiency approaches 90%. These results support the use of LysMCre-C/EBPβ^fl/fl^ mice as a model to study the functional effects of microglial C/EBPβ in vitro and in vivo.

**Fig. 7:**
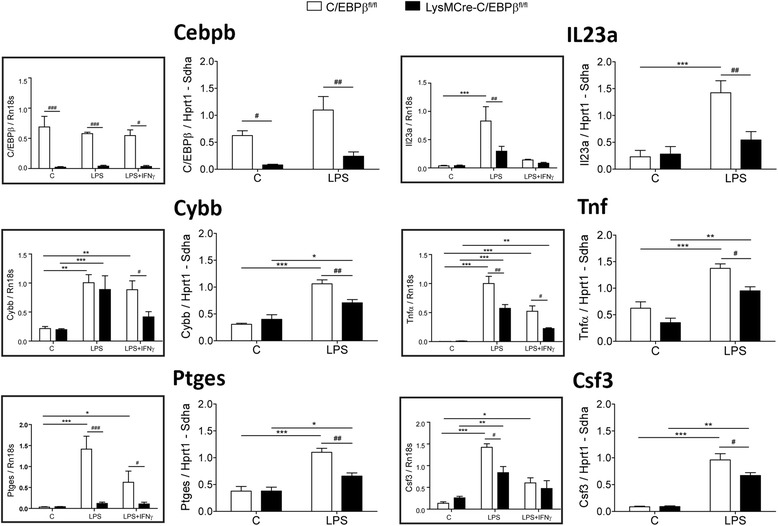
Reduced pro-inflammatory gene expression in C/EBPβ-deficient microglia in vivo and in vitro. Expression of C/EBPβ and five pro-inflammatory genes (Tnfa, Il23a, Csf3, Ptges and Cybb) was analyzed by qRT-PCR in microglia isolated from the brains of C/EBPβ^fl/fl^ and LysMCre-C/EBPβ^fl/fl^ mice treated i.p. with vehicle (C) or LPS (4 mg/kg) for 16 h (*n* = 4 mice/condition). *Insets* show the expression of the same genes in primary microglial cultures from both genotypes treated with vehicle (C), LPS (100 ng/mL) or LPS + IFNγ (1 ng/mL) for 6 h (*n* = 4 independent experiments). In all graphs, data are shown as mean + SEM. The *asterisk* shows the significance of the treatment effect, *p < 0.05; **p < 0.01; ***p < 0.001 compared with the respective control conditions. The *number sign* shows the significance of the genotype effect; #*p* < 0.05; ##*p* < 0.01; ###*p* < 0.001 compared with the respective C/EBPβ^fl/fl^ condition

To investigate the role of C/EBPβ on gene expression in activated microglia in vivo, we isolated microglia from the brains of vehicle or LPS-treated (16 h) C/EBPβ^fl/fl^ and LysMCre-C/EBPβ^fl/fl^ mice, and the expression of selected genes was assessed by quantitative real-time PCR (qRT-PCR). For this analysis, we included the pro-inflammatory cytokines Tnf, Il23a and Csf3 and the key enzymes in the production of the inflammatory mediator prostaglandin E2 (Ptges) and the free radical superoxide anion (Cybb). These are genes with a prominent role in microglial activation whose expression in cultured microglia was significantly dependent upon C/EBPβ according to RNAseq data. We first confirmed that the expression of these genes was affected by C/EBPβ by analyzing their expression by qRT-PCR in a new series of microglial cultures independent from those used to generate RNAseq data. As shown in Fig. 7 (insets), C/EBPβ deficiency resulted in decreased LPS- and/or LPS + IFNγ-induced expression of these genes in cultured microglia. Interestingly, in vivo expression of these five genes was upregulated by systemic LPS in microglia, and in all cases, these increases were significantly blunted in microglia from LysMCre-C/EBPβ^fl/fl^ mice (Fig. 7). These results demonstrate for the first time that the specific absence of C/EBPβ in microglia in vivo results in attenuated expression of pro-inflammatory genes.

### Deletion of C/EBPβ in myeloid cells is protective in an animal model of multiple sclerosis

**Fig. 8:**
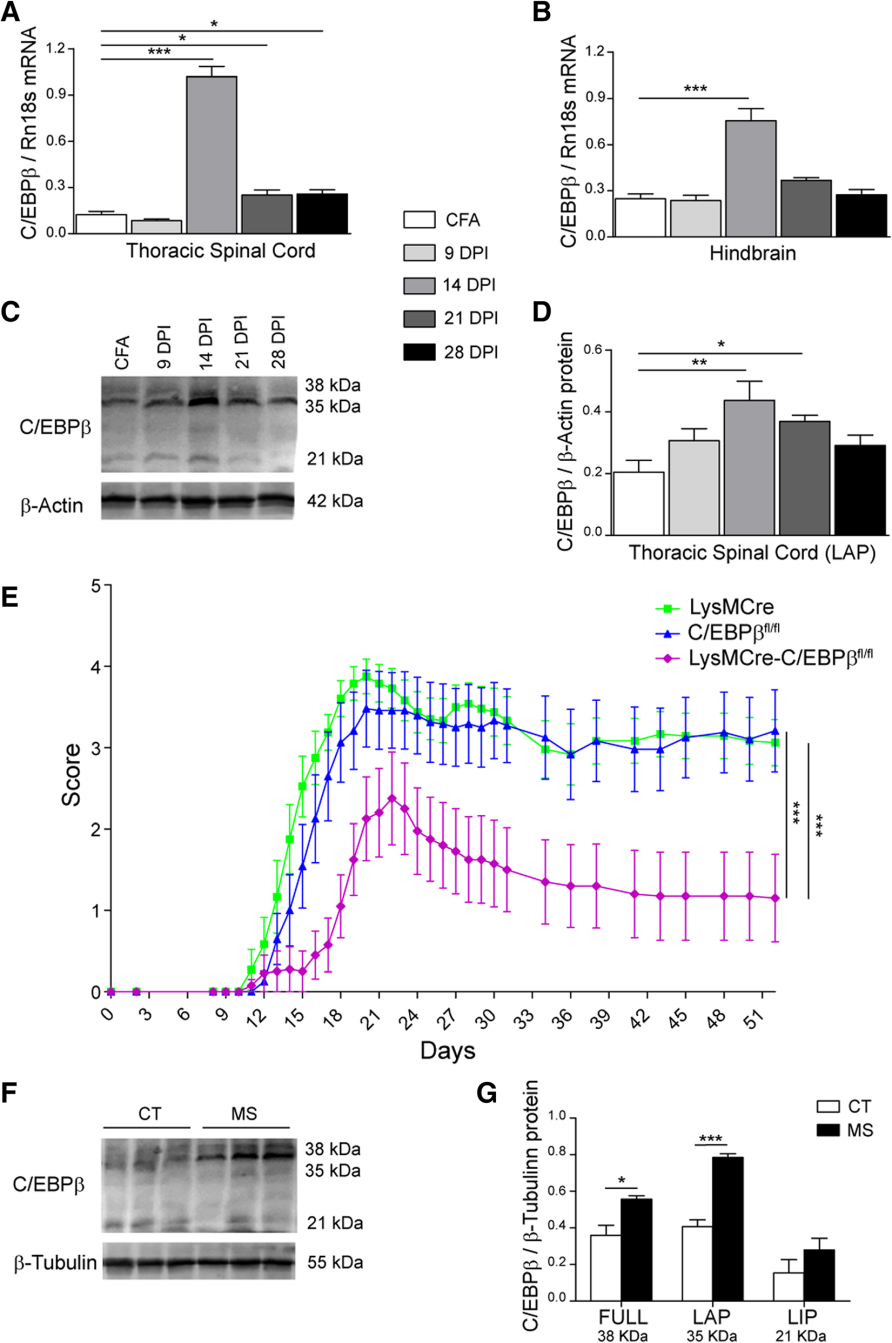
Neuroprotective effect of microglial C/EBPβ deficiency in EAE. **a**–**d** Time course analysis of C/EBPβ expression in the CNS in EAE. Expression was analyzed in vehicle-treated (CFA) mice and in EAE at 9, 14, 21 and 28 days post-injection (DPI). **a**, **b** C/EBPβ expression at mRNA level by qRT-PCR in the thoracic spinal cord (**a**) and hindbrain (**b**). **c** Representative Western blot image of C/EBPβ protein expression in the thoracic spinal cord in EAE. **d** Quantification of the C/EBPβ protein isoform LAP (*n* = 4–6 mice/condition). *p < 0.05; ** *p* < 0.01 compared with CFA. **e** EAE clinical scores of mice with myeloid C/EBPβ deficiency (LysMCre-C/EBPβ^fl/fl^ mice, *n* = 10) and the two parental control mouse lines (LysMCre, *n* = 12; C/EBPβ^fl/fl^ *n* = 12). Data show mean ± SEM. ***p < 0.001. **f** Western blot showing C/EBPβ protein levels in temporal cortex postmortem samples from three non-neurological controls (CT) and three multiple sclerosis (MS) patients. The three C/EBPβ isoforms, Full (~38 kDa), LAP (~35 kDa) and LIP (~21 kDa), are detected in these samples. **g** Quantification of C/EBPβ Western blots as that in **f**. Data show mean + SEM, *n* = 4 controls, *n* = 5 MS, *p < 0.05; ***p < 0.001

In order to test whether the myeloid-specific C/EBPβ absence and the resulting attenuation of microglial activation have neuroprotective effects in vivo, we induced EAE, an animal model of multiple sclerosis, in LysMCre-C/EBPβ^fl/fl^ mice. We first analyzed the time course of C/EBPβ expression changes in EAE in three spinal cord and three brain regions of wild-type mice. Figure 8a, b shows the results obtained in the thoracic spinal cord and hindbrain. Similar findings were obtained in the cervical and lumbar spinal cord and in the midbrain and forebrain (data not shown). EAE was associated with increased C/EBPβ mRNA levels in all regions analyzed. The most marked increase was observed on day 14. A moderate increase on days 21 and 28 was observed in some of the CNS regions analyzed, such as the thoracic spinal cord (Fig. 8a, b). To study whether these mRNA changes were also observed at the protein level, a time course analysis of C/EBPβ protein expression was performed in thoracic spinal cord by Western blot. As shown in Fig. 8c, d, a marked increase in C/EBPβ protein levels was observed on day 14, in correspondence with the mRNA data. Out of the three C/EBPβ isoforms, LAP showed the highest expression in these samples.

We next analyzed the clinical course of EAE in LysMCre-C/EBPβ^fl/fl^ mice and compared it with that of the two parent lines, LysMCre and C/EBPβ^fl/fl^. Both parent lines showed a very similar course of the disease. Mice started to develop multiple sclerosis-like symptoms 10 days after immunization. These symptoms progressed steadily, peaked on day 20 and then plateaued until the end of the experiment on day 52. In contrast, LysMCre-C/EBPβ^fl/fl^ mice showed attenuated EAE symptoms with a milder initial phase, a lower score at the peak of the disease and a partial recovery phase (Fig. 8e). Since no differences were observed between both parent lines, this experiment was repeated twice using only C/EBPβ^fl/fl^ mice as the control. In both experiments, LysMCre-C/EBPβ^fl/fl^ mice showed a highly significant reduction in EAE score with a similar profile as that of experiment 1 (data not shown). The incidence of EAE in the three experiments was similar for both genotypes (73% (30/41) for C/EBPβ^fl/fl^ mice and 68% (26/38) for LysMCre-C/EBPβ^fl/fl^ mice), and mortality was higher in C/EBPβ^fl/fl^ mice (12%; 5/41) than in LysMCre-C/EBPβ^fl/fl^ mice (5%; 2/38).

Finally, C/EBPβ expression was studied in human multiple sclerosis. To this end, C/EBPβ protein levels were analyzed by Western blot in temporal cortex postmortem samples from multiple sclerosis and non-neurological controls. As shown in Fig. 8f, g, all three C/EBPβ isoforms were detected, LAP being the most expressed. Interestingly, protein levels of Full and LAP, the two C/EBPβ activator isoforms, were increased in multiple sclerosis samples by 55% (*p* < 0.05) and 93% (*p* < 0.001), respectively.

## Discussion

C/EBPβ-deficient mice show reduced neuronal damage induced by excitotoxic or ischemic insults in vivo [7, 9]. Since C/EBPβ is expressed by many cell types, the question of which is the cell type(s) responsible for the neuroprotective effects of C/EBPβ absence in these models is of interest. In neuron–microglia co-cultures, the absence of C/EBPβ only in microglial cells completely abrogates their neurotoxic effects elicited by LPS + IFNγ activation [3]. This led us to hypothesize that C/EBPβ inhibition in the microglia could have therapeutic potential as a target to attenuate deleterious effects of neuroinflammation. As a proof of concept, we have generated mice with C/EBPβ deficiency in myeloid cells. The results here presented show a marked attenuation of clinical symptoms of EAE in these mice. Besides, RNAseq analysis of cultured microglia shows that C/EBPβ plays a key role in the regulation of gene transcription in microglial activation and provides possible explanations for the neuroprotective effects of C/EBPβ absence in myeloid cells.

In order to produce C/EBPβ deletion in microglia, we have used the Cre-LoxP approach with Cre expression under the control of the LysM promoter [27]. Unlike astrocytes, for which GFAP promoter is considered the gold standard, there is not an ideal promoter to drive Cre expression in microglia. Promoters of LysM, CD11b, F4/80, CSF1 receptor or Iba1 genes have been used successfully, and in the last few years, CX3CR1 is becoming the most widely used promoter in this respect (reviewed by [28]), but in all cases, Cre expression is induced not only exclusively in microglia but also in other subsets of myeloid cells. The LysMCre mouse line has been used extensively to induce Cre expression in myeloid cells, particularly in macrophages and microglia. Data from crossing LysMCre mice with reporter mice have shown functional Cre in 30–45% of microglial cells in control CNS [29, 30] and strong recombination in primary microglial cultures [30–32]. Various studies have obtained positive results by using LysMCre in in vivo models that have been attributed primarily to microglia [33–35] or to microglia and macrophages [31, 36]. Our data clearly show an efficiency of recombination of LysMCre close to 100 and 90% in primary microglial cultures and in microglia in vivo in LPS-treated mice, respectively, supporting the use of this mouse line to drive Cre expression in microglia in these experimental models. In our opinion, the most likely interpretation of the neuroprotective effects of LysM-driven C/EBPβ deletion in EAE is that the absence of C/EBPβ in microglia results in an attenuated neuroinflammatory response, milder neurodegeneration and less severe EAE symptoms. However, macrophages and granulocytes show also LysM-driven Cre expression in this mouse line [27], and in both cell types, C/EBPβ expression has been reported [37]. We cannot therefore discard that C/EBPβ deletion in macrophages and granulocytes accounts, at least partly, for the neuroprotective effects observed in LysMCre-C/EBPβ^fl/fl^ mice in EAE. Unfortunately, specific inhibition of C/EBPβ in microglia is not feasible at present. Strategies aimed at targeting microglia, be it nanoparticles, vectors or other approaches, cannot avoid targeting also macrophages and often other myeloid cells [38]. Although we would favor a microglial-specific strategy, these results suggest that, if unavoidable, C/EBPβ inhibition not only in microglia but also in microglia-related cells would not be necessarily undesirable.

In contrast to mice with full C/EBPβ deficiency, LysMCre-C/EBPβ^fl/fl^ mice show normal fertility and survival. This is important in this study because it allowed us to obtain genetically homogenous litters of LysMCre-C/EBPβ^fl/fl^ pups from which to prepare primary microglial cultures in sufficient amounts to thoroughly characterize the role of C/EBPβ in microglial activation in vitro. In agreement with data from microglial cultures of C/EBPβ-deficient mice [3], LysMCre-C/EBPβ^fl/fl^ microglia in culture showed normal growth and proliferation and reduced NOS2 expression and NO production upon LPS + IFNγ challenge. Since NO production is an important contributor to AICD in microglia [25], we hypothesized that AICD, a mechanism of elimination of overactivated cells best described in T cells [39], could be attenuated in LysMCre-C/EBPβ^fl/fl^ microglia. Strong AICD was observed in LPS + IFNγ-treated C/EBPβ^fl/fl^ microglial cells, but this was unaffected by C/EBPβ absence indicating that C/EBPβ-independent factors play important roles in microglial AICD. Since AICD is a protective mechanism that prevents overactivation [39], its maintenance in the absence of C/EBPβ could be a positive outcome of an eventual pharmacological strategy based on the inhibition of microglial C/EBPβ.

The RNAseq analysis is the greatest leap forward this study provides on the characterization of the role of C/EBPβ in microglia. Various studies have analyzed the role of C/EBPβ on gene expression by microarrays after genetic deletion, inhibition by RNAi or overexpression in a variety of cell types. Particularly relevant are the studies that have shown important effects of C/EBPβ on LPS + IFNγ- [40] or IFNγ- [41] induced gene expression in macrophages. Studies in other cell types such as anaplastic large cell lymphoma have demonstrated a role or C/EBPβ in the expression of immune response genes [42]. The present study is the first to analyze the role of C/EBPβ in microglia with a transcriptomic approach and also the first one to analyze the role of C/EBPβ by RNAseq in any cell type. It shows massive changes in gene expression in microglia caused by the absence of C/EBPβ. One thousand sixty-eight genes show significant differences in expression in C/EBPβ-deficient microglia. Most of these genes, 867, were also affected by LPS ± IFNγ confirming the role of C/EBPβ in microglial activation, but interestingly, C/EBPβ absence affected the expression of 201 genes that were not affected by treatment, indicating a role for C/EBPβ also in the biology of non-activated microglia. Gene Ontology enrichment analysis of the 1068 genes affected by microglial C/EBPβ deficiency identified five GO terms related to immune/inflammatory response among the 25 most significant GO terms. This is a strong confirmation of a key role for C/EBPβ in the regulation of the inflammatory gene program in microglia that others and we have proposed on the basis of the analysis of a limited number of genes [3, 7, 8, 24]. Relevant C/EBPβ-dependent genes among these classes include toll-like receptors and related proteins (Tlr1, Tlr7, Tlr8, Tlr9, Cd14, Cd180, Ly86, Nod2), cytokines (Il1a, Il12a, Il23a, Mif, Cxcl2, Cxcl3), cytokine receptors (Tnfrsf1a, Tnfrsf1b, Ccr1, Ccrl2, Csf1r) and enzymes such as inflammation-related kinases (Rps6ka4, Mapkapk2), prostaglandin synthetic enzymes (Ptges, Ptgs2 (=Cox2)) or NADPH oxidase subunits (Cybb, Ncf1). A remarkable effect of C/EBPβ deficiency was observed in the expression of Nlrp3, a key component of the inflammasome [43]. Altogether, these findings show that the phenotype of LPS ± IFNγ-treated microglia is markedly altered by the absence of C/EBPβ, showing reduced responsiveness and attenuated responses in most arms of the pro-inflammatory program. KEGG pathway enrichment analysis also identified pathways related to inflammatory responses, such as chemokine signaling or cytokine–cytokine receptor interaction, but somewhat unexpectedly, three pathways related to phagocytosis among the five most significant KEGG pathways. Particularly interesting is the group of lysosome-related genes. The significant genes in this group had a remarkably homogenous pattern of LPS ± IFNγ-induced downregulation in C/EBPβ^fl/fl^ microglia that was blunted in LysMCre-C/EBPβ^fl/fl^ microglia. These genes included degradative enzymes such as proteases (Ctss, Lgmn), nucleases (DNAse2a), sulfatases (Ids, Gns, Arsb) and glycosylases (Naga) and genes needed for lysosome acidification and assembly (Atp6v0a1, Atp6v0b, Ap1g2, Ap1s1, Ap3m2). The higher expression of lysosome genes may explain the improved ability of C/EBPβ-deficient microglia to eliminate phagocytosed bacteria that we have observed. This pattern of attenuated pro-inflammatory gene expression and improved phagocytic and digesting capacity could be of interest in a neurodegeneration context where phagocytosis of cell debris and abnormal protein deposits is necessary, whereas chronic production of pro-inflammatory mediators can be detrimental.

The possibility to acutely isolate microglial cells from the adult CNS has allowed us to estimate the efficiency of C/EBPβ deletion in LysMCre-C/EBPβ^fl/fl^ microglia in vivo, and the data obtained shows it to be very high, close to 90%. This is a critical point because it opens the possibility to use these mice to study for the first time the functional role of C/EBPβ in microglia in vivo which was the initial goal when generating this colony. In order to analyze the role of C/EBPβ on transcription of microglial genes in vivo, we selected a group of five pro-inflammatory genes (Tnfa, Il23a, Csf3, Ptges and Cybb) that (1) are regulated by C/EBPβ in microglia in culture as shown by RNAseq data; (2) have a well-established role in microglial activation; and (3) play a pathogenic role in EAE. Thus, genetic deletion of Ptges [44] and pharmacological inhibition of Tnfa [45] or Cybb [46] result in a significant attenuation of clinical symptoms of EAE, whereas genetic deletion of Il23a [47] or Csf3r [48] completely prevent the appearance of clinical EAE. The expression of these five genes in acutely isolated microglia was induced by systemic LPS injection in accordance with their pro-inflammatory character. Intriguingly, this expression was markedly attenuated in microglia isolated from LysMCre-C/EBPβ^fl/fl^ mice. This demonstrates that also in vivo C/EBPβ plays a major role in the regulation of pro-inflammatory gene expression in microglia.

Finally, robust neuroprotection from EAE was observed in LysMCre-C/EBPβ^fl/f^ mice in three independent experiments. This neuroprotective effect is probably caused by the absence of C/EBPβ not only in microglia but also in monocyte-derived macrophages since both cell types are important in EAE pathogenesis [49, 50] and LysMCre will recombine in both [28]. The abovementioned pro-inflammatory genes Tnfa, Il23a, Csf3, Ptges and Cybb are candidates to mediate this effect, but given the role of C/EBPβ as transcription factor, we favor the idea that this effect is mediated by a higher number of genes directly or indirectly regulated by C/EBPβ and that C/EBPβ absence results in a global alteration of the phenotype of activated microglia/macrophages, as demonstrated by the RNAseq analysis in cultured microglia, rather than by an effect of one or a few genes. These data strongly support the search for strategies to selectively inhibit C/EBPβ in microglia or microglia/macrophages as potential therapies in neurological disorders with a strong neuroinflammatory component. The neuroprotection observed in LysMCre-C/EBPβ^fl/fl^ mice in EAE together with the increased C/EBPβ expression in human multiple sclerosis samples point to this disease as a particularly suitable indication for such therapies.

## Conclusion

LysMCre-C/EBPβfl/fl mice show robust C/EBPβ deletion in myeloid cells and are a good tool for the study of the role of C/EBPβ in these cells. In vivo and in vitro data support an important role for C/EBPβ in the regulation of pro-inflammatory responses in microglia. Moreover, RNAseq results demonstrate the implication of C/EBPβ also in other cellular programs in activated microglia and also in non-activated microglia. Finally, C/EBPβ absence results in attenuated EAE supporting a therapeutic potential for C/EBPβ inhibition.

## Additional files

Additional file 1: Tables S1-S6. List the genes significantly up-regulated (tables 1, 3 and 5) or down-regulated (tables 2, 4 and 6) by the absence of C/EBPβ in control (tables 1, 2), LPS-treated (tables 3, 4) and LPS+IFNγ-treated (tables 5, 6) primary microglial cultures. These data were obtained by RNAseq as described in Methods. (ZIP 253 kb)
Additional file 2: Figure S1. Weighted Gene Correlation Network Analyses of microglial activation, treatment and LysMCre-C/EBPβ^fl/fl^ phenotype. A) Correlation dendrogram of genes. WGCNA algorithm was applied to filtered expression of all samples, a soft-threshold for the similarity matrix of *β* = 9 was used, and module detection was obtained with a dynamic tree cut; color bar down of the dendrogram shows module pertainance of genes with a large turquoise module corresponding to genes downregulated upon treatment with either LPS or LPS + IFNγ. B) Hierarchical clustering of detected modules by WGCNA and genotype and treatment traits; module MEyellow is the closest group of genes related to the treatment trait, whereas MEviolet is for genotype. C) Module violet heatmap (*top*) and eigengene expression graph (*bottom*): module violet is the closest group of genes related to genotype effect, contains C/EBPβ and Lyz2. D) Metacore™ network obtained by literature described interactions among the genes in module violet evidences a network of genes described to interact with C/EBPβ as a central hub. E) Correlation network obtained with a threshold for distance of 0.3, with C/EBPβ as a central hub (node size relative to degree of node). (TIF 1462 kb)

## Abbreviations

AICD: Activation-induced cell death
C/EBPβ: CCAAT/enhancer-binding protein β
DEG: Differentially expressed genes
EAE: Experimental autoimmune encephalomyelitis
GO: Gene Ontology
IFNγ: Interferon γ
KEGG: Kyoto Encyclopedia of Genes and Genomes
LPS: Lipopolysaccharide
LysM: Lysozyme M
MOG: Myelin oligodendrocyte glycoprotein peptide
PCR: Polymerase chain reaction
qRT-PCR: Quantitative real-time PCR
RNAseq: RNA sequencing
SEM: Standard error of the mean
WGCNA: Weighted Correlation Gene Network Analysis
