# Supplementary material for: Myeloid C/EBPβ deficiency reshapes microglial gene expression and is protective in experimental autoimmune encephalomyelitis
Source: J Neuroinflammation. 2017 Mar 16;14:54. doi: 10.1186/s12974-017-0834-5 (PMC5356255; doi:10.1186/s12974-017-0834-5)
Supplement: Additional file 1: Tables S1-S6. — List the genes significantly up-regulated (tables 1, 3 and 5) or down-regulated (tables 2, 4 and 6) by the absence of C/EBPβ in control (tables 1, 2), LPS-treated (tables 3, 4) and LPS+IFNγ-treated (tables 5, 6) primary microglial cultures. These data were obtained by RNAseq as described in Methods. (ZIP 253 kb) [file 12974_2017_834_MOESM1_ESM.zip › 12974_2017_834_MOESM1_ESM/Table S1.docx]

| **Table S1**  **FC:** fold change  **AveExpr**: Average expression (log2 CPM)  **adj.P:** adjusted p value  Genes are ordered by pvalue. |
| --- |
| **Genes with significantly increased expression in LysMCre-CEBPbetafl/fl microglia in control condition** |

| **GeneID** | **Length** | **FC** | **AveExpr** | **t** | **P.Value** | **adj.P** | **gene_symbol** |
| --- | --- | --- | --- | --- | --- | --- | --- |
| 65256 | 2690 | 4,0387 | 4,7113 | 6,0979 | 5,55E-06 | 0,0066 | **Asb2** |
| 80859 | 3878 | 2,4748 | 9,3132 | 6,0527 | 6,13E-06 | 0,0066 | **Nfkbiz** |
| 19024 | 4733 | 2,0752 | 7,9429 | 5,8551 | 9,51E-06 | 0,0076 | **Ppfibp2** |
| 78416 | 1072 | 3,5697 | 0,8229 | 5,3819 | 2,76E-05 | 0,0156 | **Rnase6** |
| 12517 | 1503 | 2,3519 | 7,2564 | 5,3202 | 3,18E-05 | 0,0173 | **Cd72** |
| 14347 | 1970 | 6,0076 | -0,5387 | 4,9896 | 6,81E-05 | 0,0254 | **Fut7** |
| 98365 | 1145 | 4,3567 | 6,6756 | 4,8565 | 9,27E-05 | 0,0328 | **Slamf9** |
| 109361 | 3607 | 2,6478 | 4,5482 | 4,8279 | 9,91E-05 | 0,0343 | **D730005E14Rik** |
| 214459 | 5330 | 2,6851 | 7,6323 | 4,7515 | 0,0001 | 0,0372 | **Fnbp1l** |
| 14190 | 3769 | 4,4707 | 10,2366 | 4,6528 | 0,0001 | 0,0392 | **Fgl2** |
| 232413 | 2222 | 2,8589 | 6,9968 | 4,6243 | 0,0002 | 0,0398 | **Clec12a** |
| 319997 | 1936 | 2,0916 | 7,0264 | 4,5737 | 0,0002 | 0,0433 | **A630001G21Rik** |
| 67547 | 3707 | 2,2497 | 4,3362 | 4,4596 | 0,0002 | 0,0475 | **Slc39a8** |
