# Supplementary material for: Myeloid C/EBPβ deficiency reshapes microglial gene expression and is protective in experimental autoimmune encephalomyelitis
Source: J Neuroinflammation. 2017 Mar 16;14:54. doi: 10.1186/s12974-017-0834-5 (PMC5356255; doi:10.1186/s12974-017-0834-5)
Supplement: Additional file 1: Tables S1-S6. — List the genes significantly up-regulated (tables 1, 3 and 5) or down-regulated (tables 2, 4 and 6) by the absence of C/EBPβ in control (tables 1, 2), LPS-treated (tables 3, 4) and LPS+IFNγ-treated (tables 5, 6) primary microglial cultures. These data were obtained by RNAseq as described in Methods. (ZIP 253 kb) [file 12974_2017_834_MOESM1_ESM.zip › 12974_2017_834_MOESM1_ESM/Table S2.docx]

| **Table S2**  **FC:** fold change  **AveExpr**: Average expression (log2 CPM)  **adj.P:** adjusted p value  Genes are ordered by pvalue. |
| --- |
| **Genes with significantly decreased expression in LysMCre-CEBPbetafl/fl microglia in control condition** |

| **GeneID** | **Length** | **FC** | **AveExpr** | **t** | **P.Value** | **adj.P** | **gene_symbol** |
| --- | --- | --- | --- | --- | --- | --- | --- |
| 17105 | 1057 | 0,0039 | 7,2768 | -15,5653 | 9,92E-13 | 1,51E-08 | **Lyz2** |
| 22436 | 4587 | 0,2279 | 5,7895 | -9,5844 | 5,75E-09 | 4,38E-05 | **Xdh** |
| 56615 | 943 | 0,1557 | 4,1810 | -7,8200 | 1,52E-07 | 0,0006 | **Mgst1** |
| 215772 | 5218 | 0,3676 | 4,8739 | -7,7493 | 1,75E-07 | 0,0006 | **9130014G24Rik** |
| 12608 | 1504 | 0,0331 | 3,4337 | -7,7203 | 1,86E-07 | 0,0006 | **Cebpb** |
| 241447 | 3930 | 0,4814 | 8,4141 | -7,1231 | 6,23E-07 | 0,0015 | **Lass6** |
| 64929 | 3053 | 0,0879 | -1,9587 | -7,0711 | 6,94E-07 | 0,0015 | **Scel** |
| 17394 | 2453 | 0,0383 | -0,1502 | -7,0008 | 8,03E-07 | 0,0015 | **Mmp8** |
| 20656 | 3824 | 0,2838 | 8,8128 | -6,5757 | 1,97E-06 | 0,0033 | **Sod2** |
| 19221 | 5786 | 0,1789 | 2,5296 | -6,4781 | 2,43E-06 | 0,0037 | **Ptgfrn** |
| 15251 | 4761 | 0,3606 | 8,7240 | -6,4191 | 2,76E-06 | 0,0038 | **Hif1a** |
| 140792 | 3322 | 0,4907 | 7,0504 | -6,0131 | 6,69E-06 | 0,0066 | **Colec12** |
| 545551 | 2228 | 0,2389 | 0,6231 | -6,0121 | 6,71E-06 | 0,0066 | **BC021767** |
| 218639 | 3361 | 0,3821 | 1,5100 | -5,9991 | 6,91E-06 | 0,0066 | **Arl15** |
| 244237 | 2977 | 0,3098 | 1,6267 | -5,9557 | 7,60E-06 | 0,0068 | **Tnfrsf26** |
| 17474 | 1336 | 0,2993 | 8,3203 | -5,8988 | 8,63E-06 | 0,0073 | **Clec4d** |
| 235380 | 10614 | 0,4772 | 5,9078 | -5,5918 | 1,72E-05 | 0,0119 | **Dmxl2** |
| 11847 | 1417 | 0,2199 | 2,0678 | -5,4925 | 2,15E-05 | 0,0136 | **Arg2** |
| 20288 | 3998 | 0,4469 | 8,2773 | -5,2335 | 3,88E-05 | 0,0203 | **Msr1** |
| 241576 | 3827 | 0,0993 | -0,2503 | -5,2201 | 4,00E-05 | 0,0203 | **Ldlrad3** |
| 100504452 | 3947 | 0,3564 | 4,7986 | -5,2002 | 4,19E-05 | 0,0206 | **Gm20235** |
| 231532 | 3346 | 0,3441 | 3,2099 | -5,1784 | 4,40E-05 | 0,0210 | **Arhgap24** |
| 226409 | 4154 | 0,4693 | 1,5567 | -5,1474 | 4,73E-05 | 0,0216 | **Zranb3** |
| 269823 | 1784 | 0,4957 | 4,7309 | -5,1376 | 4,84E-05 | 0,0216 | **Pon3** |
| 15442 | 3082 | 0,3370 | 4,0677 | -5,1141 | 5,11E-05 | 0,0216 | **Hpse** |
| 13723 | 2622 | 0,2184 | 2,5350 | -5,0910 | 5,39E-05 | 0,0222 | **Emb** |
| 100861753 | 1669 | 0,3614 | 3,2949 | -4,9872 | 6,85E-05 | 0,0254 |  |
| 442827 | 4105 | 0,1347 | -0,5691 | -4,8086 | 0,0001 | 0,0346 | **Rab44** |
| 19152 | 972 | 0,2482 | -2,3015 | -4,7879 | 0,0001 | 0,0352 | **Prtn3** |
| 14544 | 5417 | 0,0968 | 3,3991 | -4,7325 | 0,0001 | 0,0372 | **Gda** |
| 21944 | 1694 | 0,4780 | 1,5578 | -4,7323 | 0,0001 | 0,0372 | **Tnfsf12** |
| 432466 | 1550 | 0,3491 | 4,4396 | -4,7087 | 0,0001 | 0,0383 | **Gm5424** |
| 17319 | 554 | 0,4309 | 4,3736 | -4,6997 | 0,0001 | 0,0384 | **Mif** |
| 12494 | 2995 | 0,2355 | 5,5751 | -4,6741 | 0,0001 | 0,0392 | **Cd38** |
| 233781 | 3070 | 0,4029 | 2,2005 | -4,6518 | 0,0001 | 0,0392 | **Xylt1** |
| 20342 | 1695 | 0,1484 | -0,6571 | -4,6400 | 0,0002 | 0,0396 | **Selenbp2** |
| 216881 | 2739 | 0,2057 | -0,1736 | -4,6325 | 0,0002 | 0,0397 | **Wscd1** |
| 20677 | 2967 | 0,2773 | 2,8663 | -4,5546 | 0,0002 | 0,0442 | **Sox4** |
| 66569 | 2484 | 0,4468 | 3,2234 | -4,5484 | 0,0002 | 0,0442 | **Gdpd1** |
| 16365 | 2588 | 0,3427 | 9,7944 | -4,5459 | 0,0002 | 0,0442 | **Irg1** |
| 384783 | 4015 | 0,3637 | 2,3524 | -4,5171 | 0,0002 | 0,0450 | **Irs2** |
| 107272 | 2491 | 0,4726 | 6,0567 | -4,5087 | 0,0002 | 0,0450 | **Psat1** |
| 27226 | 1915 | 0,2373 | 5,7935 | -4,5051 | 0,0002 | 0,0450 | **Pla2g7** |
| 320099 | 2643 | 0,3353 | -0,7858 | -4,5009 | 0,0002 | 0,0450 | **BC106179** |
