# Supplementary material for: Myeloid C/EBPβ deficiency reshapes microglial gene expression and is protective in experimental autoimmune encephalomyelitis
Source: J Neuroinflammation. 2017 Mar 16;14:54. doi: 10.1186/s12974-017-0834-5 (PMC5356255; doi:10.1186/s12974-017-0834-5)
Supplement: Additional file 1: Tables S1-S6. — List the genes significantly up-regulated (tables 1, 3 and 5) or down-regulated (tables 2, 4 and 6) by the absence of C/EBPβ in control (tables 1, 2), LPS-treated (tables 3, 4) and LPS+IFNγ-treated (tables 5, 6) primary microglial cultures. These data were obtained by RNAseq as described in Methods. (ZIP 253 kb) [file 12974_2017_834_MOESM1_ESM.zip › 12974_2017_834_MOESM1_ESM/Table S3.docx]

| **Table S3** |
| --- |
| **Genes with significantly increased expression in LysMCre-CEBPbetafl/fl microglia in LPS condition** |

| **GeneID** | **Length** | **FC** | **AveExpr** | **t** | **P.Value** | **adj.P** | **gene_symbol** |
| --- | --- | --- | --- | --- | --- | --- | --- |
| 20947 | 4035 | 4,2532 | 9,3066 | 11,6741 | 1,91E-10 | 2,29E-07 | **Swap70** |
| 12183 | 2068 | 6,5921 | 3,5006 | 11,6591 | 1,95E-10 | 2,29E-07 | **Bpgm** |
| 99138 | 3118 | 4,3481 | 6,7911 | 10,1709 | 2,10E-09 | 1,23E-06 | **Stard7** |
| 74048 | 4846 | 3,0314 | 9,0228 | 10,0615 | 2,53E-09 | 1,43E-06 | **4632428N05Rik** |
| 207278 | 4411 | 3,1002 | 6,8304 | 9,8642 | 3,54E-09 | 1,82E-06 | **Fchsd2** |
| 239667 | 9063 | 3,4854 | 7,8966 | 9,4289 | 7,55E-09 | 3,59E-06 | **Dip2b** |
| 67292 | 3301 | 3,3277 | 4,5377 | 9,3198 | 9,16E-09 | 4,10E-06 | **Pigc** |
| 246177 | 3307 | 4,2622 | 7,6591 | 9,2474 | 1,04E-08 | 4,29E-06 | **Myo1g** |
| 21813 | 4788 | 2,9406 | 7,8900 | 9,2326 | 1,07E-08 | 4,29E-06 | **Tgfbr2** |
| 74006 | 4058 | 2,2010 | 7,0801 | 9,1113 | 1,33E-08 | 4,94E-06 | **Dnm1l** |
| 238328 | 6189 | 3,0608 | 5,6338 | 8,6819 | 2,92E-08 | 9,13E-06 | **Vash1** |
| 70397 | 1732 | 2,5579 | 4,6301 | 8,6704 | 2,99E-08 | 9,13E-06 | **Tmem70** |
| 58231 | 5189 | 2,3705 | 8,0728 | 8,6284 | 3,23E-08 | 9,65E-06 | **Stk4** |
| 16491 | 1968 | 3,0643 | 4,5127 | 8,4174 | 4,80E-08 | 1,35E-05 | **Kcna3** |
| 66854 | 3684 | 3,1770 | 7,5934 | 8,3343 | 5,62E-08 | 1,54E-05 | **Trim35** |
| 18479 | 3061 | 3,9031 | 5,0213 | 8,2725 | 6,33E-08 | 1,59E-05 | **Pak1** |
| 23912 | 2143 | 7,2366 | 2,7176 | 8,2643 | 6,43E-08 | 1,59E-05 | **Rhof** |
| 232406 | 2742 | 6,9067 | 2,9877 | 8,2527 | 6,57E-08 | 1,59E-05 | **BC035044** |
| 67164 | 1229 | 12,8292 | 2,4114 | 8,1495 | 8,02E-08 | 1,85E-05 | **Lipt2** |
| 224143 | 2641 | 4,6058 | 5,5251 | 8,0270 | 1,02E-07 | 2,15E-05 | **Poglut1** |
| 75957 | 2339 | 6,2330 | 3,2479 | 7,9787 | 1,12E-07 | 2,30E-05 | **Mir17hg** |
| 26382 | 2625 | 5,1233 | 6,8087 | 7,8086 | 1,56E-07 | 3,00E-05 | **Fgd2** |
| 211948 | 3529 | 2,8214 | 4,9152 | 7,7665 | 1,69E-07 | 3,16E-05 | **Pde12** |
| 11975 | 4029 | 3,4530 | 7,6104 | 7,7426 | 1,78E-07 | 3,26E-05 | **Atp6v0a1** |
| 229055 | 7449 | 3,6508 | 3,7863 | 7,7180 | 1,87E-07 | 3,38E-05 | **Zbtb10** |
| 217232 | 5783 | 2,3238 | 7,1335 | 7,6634 | 2,08E-07 | 3,68E-05 | **Cdc27** |
| 19889 | 3786 | 2,3804 | 7,5047 | 7,6391 | 2,18E-07 | 3,73E-05 | **Rp2h** |
| 15357 | 4430 | 2,4974 | 7,1001 | 7,5441 | 2,64E-07 | 4,32E-05 | **Hmgcr** |
| 66058 | 1119 | 2,3034 | 7,5960 | 7,5070 | 2,85E-07 | 4,51E-05 | **Tmem176a** |
| 69780 | 2905 | 2,8171 | 7,4942 | 7,4951 | 2,91E-07 | 4,57E-05 | **Smap2** |
| 11797 | 3140 | 2,2462 | 6,5651 | 7,4536 | 3,17E-07 | 4,92E-05 | **Birc2** |
| 26888 | 2616 | 4,1645 | 6,7746 | 7,4338 | 3,30E-07 | 5,02E-05 | **Clec4a2** |
| 71902 | 8209 | 2,2626 | 7,1517 | 7,3863 | 3,63E-07 | 5,32E-05 | **Cand1** |
| 16561 | 14849 | 2,8281 | 8,4352 | 7,3327 | 4,05E-07 | 5,76E-05 | **Kif1b** |
| 67710 | 868 | 2,1845 | 6,0476 | 7,3028 | 4,31E-07 | 6,01E-05 | **Polr2g** |
| 19655 | 3743 | 4,3606 | 4,6235 | 7,3000 | 4,33E-07 | 6,01E-05 | **Rbmx** |
| 226525 | 10046 | 3,8844 | 5,3767 | 7,2989 | 4,34E-07 | 6,01E-05 | **Rasal2** |
| 101148 | 4141 | 4,0261 | 6,5854 | 7,1475 | 5,92E-07 | 7,91E-05 | **B630005N14Rik** |
| 70314 | 2539 | 2,4687 | 6,0261 | 7,1405 | 6,01E-07 | 7,96E-05 | **Rabep2** |
| 100041286 | 592 | 3,8086 | 3,2055 | 7,1124 | 6,37E-07 | 8,29E-05 | **Gm11974** |
| 28185 | 3778 | 2,0689 | 7,4947 | 7,0970 | 6,57E-07 | 8,48E-05 | **Tomm70a** |
| 67846 | 2934 | 3,7536 | 5,9478 | 7,0876 | 6,70E-07 | 8,58E-05 | **Tmem39a** |
| 56758 | 5809 | 2,7154 | 9,0985 | 7,0394 | 7,41E-07 | 9,02E-05 | **Mbnl1** |
| 66540 | 3148 | 3,0346 | 7,5408 | 7,0357 | 7,47E-07 | 9,02E-05 | **Fam107b** |
| 320333 | 1413 | 13,0483 | -1,2442 | 6,9592 | 8,75E-07 | 0,0001 | **D830030K20Rik** |
| 17918 | 11684 | 2,4541 | 8,5373 | 6,9148 | 9,61E-07 | 0,0001 | **Myo5a** |
| 230674 | 4956 | 2,3462 | 7,5160 | 6,8745 | 1,05E-06 | 0,0001 | **Kdm4a** |
| 224109 | 3559 | 2,8404 | 7,5400 | 6,8456 | 1,11E-06 | 0,0001 | **Lrrc33** |
| 271424 | 2305 | 12,3711 | -1,3042 | 6,8091 | 1,20E-06 | 0,0001 | **Ip6k3** |
| 12048 | 2371 | 2,1563 | 7,3602 | 6,7930 | 1,24E-06 | 0,0001 | **Bcl2l1** |
| 67501 | 6981 | 2,0870 | 8,9092 | 6,7809 | 1,27E-06 | 0,0001 | **Ccdc50** |
| 74182 | 5052 | 3,4462 | 5,8985 | 6,7634 | 1,32E-06 | 0,0001 | **Gpcpd1** |
| 21372 | 2302 | 2,2001 | 6,6717 | 6,7157 | 1,46E-06 | 0,0002 | **Tbl1x** |
| 106585 | 8991 | 3,2816 | 6,6818 | 6,7045 | 1,50E-06 | 0,0002 | **Ankrd12** |
| 18711 | 11255 | 2,0045 | 7,7696 | 6,6724 | 1,60E-06 | 0,0002 | **Pikfyve** |
| 15900 | 2829 | 2,4807 | 8,9418 | 6,6032 | 1,86E-06 | 0,0002 | **Irf8** |
| 71653 | 4081 | 2,7722 | 5,7887 | 6,5901 | 1,91E-06 | 0,0002 | **4930506M07Rik** |
| 56434 | 1729 | 2,3470 | 8,4663 | 6,5866 | 1,92E-06 | 0,0002 | **Tspan3** |
| 13665 | 3152 | 2,1857 | 6,6240 | 6,5861 | 1,93E-06 | 0,0002 | **Eif2s1** |
| 331474 | 4766 | 4,8503 | 4,8543 | 6,3988 | 2,88E-06 | 0,0003 | **Rgag4** |
| 73341 | 4569 | 6,0806 | 5,7759 | 6,3776 | 3,02E-06 | 0,0003 | **Arhgef6** |
| 26879 | 1901 | 2,1828 | 6,1695 | 6,3491 | 3,21E-06 | 0,0003 | **B3galnt1** |
| 15481 | 2104 | 2,4742 | 8,1534 | 6,3240 | 3,39E-06 | 0,0003 | **Hspa8** |
| 226551 | 6308 | 3,9287 | 8,0283 | 6,2454 | 4,02E-06 | 0,0004 | **AI848100** |
| 54366 | 3687 | 7,6998 | 1,6122 | 6,1844 | 4,59E-06 | 0,0004 | **Ctnnal1** |
| 15488 | 2685 | 2,1009 | 6,1614 | 6,0841 | 5,73E-06 | 0,0005 | **Hsd17b4** |
| 94045 | 2288 | 8,9054 | 0,6435 | 6,0723 | 5,88E-06 | 0,0005 | **P2rx5** |
| 269587 | 6923 | 2,1204 | 6,2009 | 6,0683 | 5,93E-06 | 0,0005 | **Epb4.1** |
| 14154 | 6816 | 2,8514 | 5,2409 | 6,0540 | 6,12E-06 | 0,0005 | **Fem1a** |
| 108911 | 3767 | 2,6691 | 5,9589 | 6,0264 | 6,50E-06 | 0,0005 | **Rcc2** |
| 72057 | 1665 | 4,0402 | 5,6336 | 6,0022 | 6,86E-06 | 0,0005 | **Phf10** |
| 108735 | 5526 | 2,0697 | 6,5541 | 5,9851 | 7,12E-06 | 0,0005 | **Sft2d2** |
| 101118 | 4548 | 2,0641 | 7,0618 | 5,9664 | 7,42E-06 | 0,0006 | **Tmem168** |
| 229709 | 3851 | 2,2251 | 6,4619 | 5,9608 | 7,52E-06 | 0,0006 | **Ahcyl1** |
| 94190 | 3932 | 2,4888 | 6,5729 | 5,9130 | 8,36E-06 | 0,0006 | **Ophn1** |
| 53319 | 2284 | 2,2574 | 8,2175 | 5,9072 | 8,47E-06 | 0,0006 | **Nxf1** |
| 414084 | 3769 | 2,3658 | 7,3176 | 5,8942 | 8,72E-06 | 0,0006 | **Tnip3** |
| 57753 | 3977 | 2,5414 | 4,2385 | 5,8883 | 8,83E-06 | 0,0006 | **Noc3l** |
| 229694 | 5544 | 2,7720 | 5,2407 | 5,8795 | 9,00E-06 | 0,0006 | **AI504432** |
| 71389 | 10549 | 2,6028 | 6,6093 | 5,8782 | 9,03E-06 | 0,0006 | **Chd6** |
| 66645 | 2375 | 2,1121 | 4,7593 | 5,8651 | 9,30E-06 | 0,0006 | **Pspc1** |
| 12607 | 4077 | 2,4487 | 4,6374 | 5,8595 | 9,42E-06 | 0,0007 | **Cebpz** |
| 70974 | 8626 | 4,2170 | 5,3103 | 5,8519 | 9,57E-06 | 0,0007 | **Pgm2l1** |
| 71302 | 7899 | 4,4015 | 3,3822 | 5,8486 | 9,65E-06 | 0,0007 | **Arhgap26** |
| 102442 | 8655 | 2,6068 | 6,6561 | 5,8208 | 1,03E-05 | 0,0007 | **Dennd4a** |
| 232670 | 1959 | 3,3280 | 5,1125 | 5,8132 | 1,04E-05 | 0,0007 | **Tspan33** |
| 18436 | 2441 | 16,0372 | 1,8063 | 5,8101 | 1,05E-05 | 0,0007 | **P2rx1** |
| 14190 | 3769 | 5,2143 | 10,2366 | 5,7959 | 1,09E-05 | 0,0007 | **Fgl2** |
| 66209 | 3221 | 2,0648 | 4,5875 | 5,7867 | 1,11E-05 | 0,0007 | **1110054O05Rik** |
| 18442 | 3000 | 2,9200 | 3,9031 | 5,7411 | 1,23E-05 | 0,0008 | **P2ry2** |
| 215690 | 12767 | 3,5748 | 5,2083 | 5,7348 | 1,24E-05 | 0,0008 | **Nav1** |
| 66286 | 770 | 3,5468 | 4,5038 | 5,7083 | 1,32E-05 | 0,0008 | **Sec11c** |
| 116940 | 4328 | 2,0136 | 7,1819 | 5,7071 | 1,32E-05 | 0,0008 | **Tgs1** |
| 26922 | 1337 | 3,1424 | 4,1650 | 5,6942 | 1,36E-05 | 0,0008 | **Mecr** |
| 100041874 | 2149 | 10,7202 | -0,3791 | 5,6691 | 1,44E-05 | 0,0009 | **Gm3558** |
| 18139 | 6470 | 2,0453 | 7,0374 | 5,6658 | 1,45E-05 | 0,0009 | **Zfml** |
| 74392 | 6438 | 2,0041 | 7,7709 | 5,6449 | 1,52E-05 | 0,0009 | **Specc1l** |
| 20439 | 2488 | 2,2345 | 5,5680 | 5,6262 | 1,59E-05 | 0,0009 | **Siah2** |
| 69956 | 2595 | 2,0994 | 4,7809 | 5,6156 | 1,63E-05 | 0,0009 | **Ptcd3** |
| 104001 | 3710 | 2,9276 | 4,6930 | 5,6108 | 1,64E-05 | 0,0010 | **Rtn1** |
| 17101 | 11806 | 2,2684 | 8,7557 | 5,5996 | 1,69E-05 | 0,0010 | **Lyst** |
| 232906 | 8393 | 2,1030 | 6,2293 | 5,5995 | 1,69E-05 | 0,0010 | **Grlf1** |
| 100041279 | 618 | 8,1506 | 0,0743 | 5,5633 | 1,83E-05 | 0,0010 | **Gm3248** |
| 57815 | 2910 | 2,0792 | 5,2942 | 5,5316 | 1,97E-05 | 0,0011 | **Spata5** |
| 338359 | 2390 | 2,4338 | 3,5838 | 5,5161 | 2,04E-05 | 0,0011 | **Supv3l1** |
| 18020 | 3388 | 2,2728 | 3,1974 | 5,4899 | 2,16E-05 | 0,0012 | **Nfatc2ip** |
| 474145 | 1092 | 9,0469 | 1,3193 | 5,4796 | 2,21E-05 | 0,0012 | **Clec4a4** |
| 26465 | 2031 | 3,1141 | 4,4428 | 5,4655 | 2,28E-05 | 0,0012 | **Zfp146** |
| 210992 | 1605 | 2,0536 | 6,5939 | 5,4517 | 2,36E-05 | 0,0012 | **Lpcat1** |
| 12478 | 2436 | 10,5544 | -1,3464 | 5,4455 | 2,39E-05 | 0,0012 | **Cd19** |
| 66871 | 3715 | 2,2137 | 4,9224 | 5,4335 | 2,46E-05 | 0,0013 | **Cpne8** |
| 20437 | 2114 | 2,1510 | 4,1312 | 5,4226 | 2,52E-05 | 0,0013 | **Siah1a** |
| 11852 | 2228 | 2,3415 | 6,5845 | 5,4224 | 2,52E-05 | 0,0013 | **Rhob** |
| 19141 | 1874 | 2,0967 | 10,3848 | 5,4009 | 2,65E-05 | 0,0013 | **Lgmn** |
| 30877 | 1884 | 2,7682 | 5,1783 | 5,3951 | 2,68E-05 | 0,0013 | **Gnl3** |
| 77938 | 5199 | 2,4931 | 4,9093 | 5,3869 | 2,73E-05 | 0,0014 | **Fam53b** |
| 228790 | 6674 | 2,3324 | 5,8844 | 5,3652 | 2,87E-05 | 0,0014 | **Asxl1** |
| 108888 | 2419 | 3,1386 | 4,2862 | 5,3471 | 2,99E-05 | 0,0015 | **Atad3a** |
| 28114 | 4279 | 2,0802 | 6,1817 | 5,3257 | 3,14E-05 | 0,0015 | **Nsun2** |
| 232288 | 5029 | 2,0711 | 7,4085 | 5,3010 | 3,32E-05 | 0,0016 | **Frmd4b** |
| 20496 | 6520 | 2,9306 | 5,8864 | 5,2921 | 3,39E-05 | 0,0016 | **Slc12a2** |
| 16010 | 2069 | 2,5988 | 6,4560 | 5,2838 | 3,46E-05 | 0,0016 | **Igfbp4** |
| 330096 | 3754 | 2,7378 | 2,8161 | 5,2682 | 3,58E-05 | 0,0017 | **Shisa3** |
| 72750 | 5532 | 3,6424 | 5,2640 | 5,2449 | 3,78E-05 | 0,0018 | **Fam117b** |
| 64209 | 1871 | 2,9381 | 6,6408 | 5,2336 | 3,88E-05 | 0,0018 | **Herpud1** |
| 55984 | 3459 | 10,5820 | 1,6889 | 5,2316 | 3,90E-05 | 0,0018 | **Camkk1** |
| 69617 | 3543 | 2,1381 | 6,2586 | 5,2245 | 3,96E-05 | 0,0018 | **Pitrm1** |
| 243983 | 2407 | 2,7446 | 4,8359 | 5,2191 | 4,01E-05 | 0,0018 | **Zdhhc13** |
| 17691 | 4509 | 4,6404 | 5,5620 | 5,2047 | 4,15E-05 | 0,0019 | **Sik1** |
| 66978 | 6636 | 2,1542 | 5,6499 | 5,1976 | 4,21E-05 | 0,0019 | **Luc7l** |
| 12971 | 1256 | 5,7165 | 0,3375 | 5,1795 | 4,39E-05 | 0,0019 | **Crym** |
| 68169 | 4684 | 5,7362 | 0,7674 | 5,1538 | 4,66E-05 | 0,0020 | **A930038C07Rik** |
| 241230 | 3161 | 10,6225 | 1,5368 | 5,1537 | 4,66E-05 | 0,0020 | **St8sia6** |
| 56857 | 4436 | 2,1484 | 6,4184 | 5,1362 | 4,85E-05 | 0,0021 | **Slc37a2** |
| 11307 | 5821 | 2,6459 | 7,9340 | 5,1312 | 4,91E-05 | 0,0021 | **Abcg1** |
| 67092 | 2357 | 3,9818 | 6,3207 | 5,1263 | 4,97E-05 | 0,0021 | **Gatm** |
| 28071 | 2324 | 2,1583 | 3,7857 | 5,1125 | 5,13E-05 | 0,0022 | **Twistnb** |
| 67134 | 1895 | 2,5259 | 5,3094 | 5,1041 | 5,23E-05 | 0,0022 | **Nop56** |
| 78334 | 5807 | 2,3535 | 4,9378 | 5,1015 | 5,26E-05 | 0,0022 | **Cdk19** |
| 327900 | 3032 | 2,2833 | 4,0953 | 5,1004 | 5,27E-05 | 0,0022 | **Ubtd2** |
| 11566 | 2553 | 2,3116 | 5,4348 | 5,0971 | 5,31E-05 | 0,0022 | **Adss** |
| 63872 | 1530 | 4,0155 | 0,6886 | 5,0937 | 5,35E-05 | 0,0022 | **Zfp296** |
| 12517 | 1503 | 2,3306 | 7,2564 | 5,0872 | 5,43E-05 | 0,0023 | **Cd72** |
| 170938 | 2948 | 2,2642 | 3,9700 | 5,0747 | 5,59E-05 | 0,0023 | **Zfp617** |
| 216825 | 4442 | 2,2696 | 6,3399 | 5,0607 | 5,78E-05 | 0,0024 | **Usp22** |
| 73149 | 1330 | 2,7687 | 6,1703 | 5,0539 | 5,87E-05 | 0,0024 | **Clec4a3** |
| 68162 | 1126 | 4,2572 | 1,9369 | 5,0406 | 6,05E-05 | 0,0024 | **A930003A15Rik** |
| 52633 | 1286 | 2,1086 | 4,8104 | 5,0280 | 6,23E-05 | 0,0025 | **Nit2** |
| 217721 | 3435 | 5,8990 | 3,1364 | 4,9962 | 6,71E-05 | 0,0026 | **Mfsd7c** |
| 19058 | 2829 | 3,1935 | 6,5465 | 4,9908 | 6,79E-05 | 0,0026 | **Ppp3r1** |
| 232414 | 3294 | 7,4389 | 0,1064 | 4,9866 | 6,86E-05 | 0,0026 | **Clec9a** |
| 226594 | 2670 | 2,0148 | 7,0621 | 4,9739 | 7,06E-05 | 0,0027 | **Rcsd1** |
| 11801 | 2037 | 3,9128 | 3,9169 | 4,9703 | 7,12E-05 | 0,0027 | **Cd5l** |
| 69900 | 3128 | 2,5268 | 5,4107 | 4,9673 | 7,17E-05 | 0,0027 | **Mfsd11** |
| 19893 | 6751 | 4,3333 | 2,4606 | 4,9652 | 7,20E-05 | 0,0027 | **Rpgr** |
| 69237 | 2579 | 2,1016 | 5,9316 | 4,9619 | 7,26E-05 | 0,0027 | **Gtpbp4** |
| 80907 | 2031 | 2,8945 | 3,7718 | 4,9587 | 7,31E-05 | 0,0027 | **Lactb** |
| 100040852 | 5217 | 4,1901 | 0,1807 | 4,9504 | 7,45E-05 | 0,0028 | **Gm3002** |
| 67041 | 3482 | 2,2813 | 6,4339 | 4,9393 | 7,65E-05 | 0,0028 | **Oxct1** |
| 192652 | 6909 | 2,2079 | 7,1577 | 4,9247 | 7,91E-05 | 0,0029 | **Wdr81** |
| 66836 | 748 | 2,6076 | 3,5594 | 4,9218 | 7,97E-05 | 0,0029 | **Tmem223** |
| 52575 | 1846 | 2,6461 | 3,6426 | 4,9161 | 8,07E-05 | 0,0029 | **Rg9mtd1** |
| 80291 | 1260 | 2,0487 | 7,1276 | 4,8890 | 8,60E-05 | 0,0031 | **Rilpl2** |
| 17330 | 2619 | 2,0887 | 6,3068 | 4,8875 | 8,63E-05 | 0,0031 | **Minpp1** |
| 17973 | 1685 | 2,3412 | 5,3352 | 4,8732 | 8,92E-05 | 0,0032 | **Nck1** |
| 100504591 | 1082 | 4,0978 | 4,4864 | 4,8674 | 9,04E-05 | 0,0032 | **Gm15987** |
| 11977 | 8146 | 2,5212 | 7,5797 | 4,8647 | 9,09E-05 | 0,0032 | **Atp7a** |
| 20312 | 3144 | 2,8589 | 4,8154 | 4,8632 | 9,13E-05 | 0,0032 | **Cx3cl1** |
| 100504333 | 1960 | 2,3414 | 4,2975 | 4,8273 | 9,92E-05 | 0,0034 | **Gm16712** |
| 72699 | 1959 | 2,0096 | 4,6916 | 4,8163 | 0,0001 | 0,0034 | **Lime1** |
| 215653 | 4785 | 3,0539 | 6,2232 | 4,8048 | 0,0001 | 0,0035 | **Rassf2** |
| 224065 | 835 | 11,3615 | -0,8431 | 4,7944 | 0,0001 | 0,0036 | **Uts2d** |
| 140580 | 5874 | 2,6326 | 6,9483 | 4,7906 | 0,0001 | 0,0036 | **Elmo1** |
| 234733 | 6750 | 2,1860 | 5,3449 | 4,7897 | 0,0001 | 0,0036 | **Ddx19b** |
| 66926 | 2820 | 2,0689 | 4,0986 | 4,7888 | 0,0001 | 0,0036 | **Trmt6** |
| 210710 | 2129 | 8,1367 | 2,9236 | 4,7827 | 0,0001 | 0,0036 | **Gab3** |
| 74148 | 5399 | 3,0107 | 5,5231 | 4,7743 | 0,0001 | 0,0037 | **1300001I01Rik** |
| 216001 | 2275 | 2,0511 | 5,4867 | 4,7712 | 0,0001 | 0,0037 | **Cbara1** |
| 110109 | 2623 | 2,7050 | 5,3300 | 4,7707 | 0,0001 | 0,0037 | **Nop2** |
| 20621 | 2904 | 2,5595 | 5,6582 | 4,7513 | 0,0001 | 0,0038 | **Snn** |
| 83924 | 2981 | 2,1364 | 5,6250 | 4,7504 | 0,0001 | 0,0038 | **Gpr137b** |
| 195727 | 5830 | 5,4322 | 3,3937 | 4,7473 | 0,0001 | 0,0039 | **Nhs** |
| 20299 | 1811 | 7,7662 | 6,5171 | 4,7471 | 0,0001 | 0,0039 | **Ccl22** |
| 12043 | 7749 | 2,0981 | 4,9840 | 4,7319 | 0,0001 | 0,0040 | **Bcl2** |
| 104759 | 1978 | 3,0684 | 6,7981 | 4,7314 | 0,0001 | 0,0040 | **Pld4** |
| 100503654 | 3662 | 3,3628 | 4,9148 | 4,7207 | 0,0001 | 0,0040 | **Gm19816** |
| 229473 | 4909 | 3,3504 | 5,6857 | 4,7113 | 0,0001 | 0,0041 | **D930015E06Rik** |
| 100637 | 1906 | 3,7629 | 4,5383 | 4,6990 | 0,0001 | 0,0042 | **N4bp2l1** |
| 654804 | 1887 | 6,4090 | 0,7286 | 4,6881 | 0,0001 | 0,0043 | **4732471J01Rik** |
| 110816 | 3871 | 2,1672 | 4,5405 | 4,6795 | 0,0001 | 0,0044 | **Pwp2** |
| 230661 | 3032 | 5,8860 | 2,9797 | 4,6748 | 0,0001 | 0,0044 | **Tesk2** |
| 20850 | 3888 | 2,3858 | 6,1213 | 4,6742 | 0,0001 | 0,0044 | **Stat5a** |
| 66580 | 3392 | 2,0519 | 4,2939 | 4,6731 | 0,0001 | 0,0044 | **Esf1** |
| 67016 | 5936 | 2,1002 | 7,4201 | 4,6687 | 0,0001 | 0,0044 | **Tbc1d2b** |
| 74237 | 3085 | 2,2065 | 4,3519 | 4,6677 | 0,0001 | 0,0044 | **Tubgcp2** |
| 72181 | 3511 | 2,0768 | 4,3430 | 4,6549 | 0,0001 | 0,0045 | **Nsun4** |
| 215351 | 4938 | 2,4000 | 6,2833 | 4,6545 | 0,0001 | 0,0045 | **Senp6** |
| 76688 | 2596 | 2,0378 | 4,8468 | 4,6164 | 0,0002 | 0,0048 | **Arfrp1** |
| 11855 | 5197 | 2,6783 | 5,5429 | 4,6162 | 0,0002 | 0,0048 | **Arhgap5** |
| 216892 | 3241 | 5,2365 | 0,5253 | 4,5984 | 0,0002 | 0,0050 | **Spns2** |
| 67928 | 5384 | 7,8342 | -0,6288 | 4,5980 | 0,0002 | 0,0050 | **Abca14** |
| 19294 | 3444 | 2,5353 | 6,4499 | 4,5925 | 0,0002 | 0,0050 | **Pvrl2** |
| 22289 | 5265 | 2,3734 | 5,8981 | 4,5814 | 0,0002 | 0,0051 | **Kdm6a** |
| 57377 | 2770 | 2,9071 | 5,1176 | 4,5620 | 0,0002 | 0,0053 | **Mogs** |
| 69091 | 6873 | 2,1193 | 5,1452 | 4,5584 | 0,0002 | 0,0053 | **Vps26b** |
| 244152 | 2800 | 3,5185 | 4,7715 | 4,5554 | 0,0002 | 0,0053 | **Tsku** |
| 20338 | 6300 | 2,1705 | 8,7109 | 4,5520 | 0,0002 | 0,0053 | **Sel1l** |
| 666747 | 2262 | 6,1500 | -0,1580 | 4,5429 | 0,0002 | 0,0054 | **Trim43b** |
| 57874 | 2733 | 2,0163 | 5,7485 | 4,5360 | 0,0002 | 0,0055 | **Ptplad1** |
| 245000 | 8074 | 2,1003 | 5,4299 | 4,5326 | 0,0002 | 0,0055 | **Atr** |
| 20361 | 3275 | 6,3861 | 5,1555 | 4,5267 | 0,0002 | 0,0055 | **Sema7a** |
| 68927 | 1675 | 2,4745 | 3,3411 | 4,5242 | 0,0002 | 0,0056 | **Ptcd2** |
| 69499 | 3805 | 2,5372 | 2,8011 | 4,5136 | 0,0002 | 0,0056 | **Tsr2** |
| 19130 | 4139 | 2,2507 | 3,5488 | 4,5064 | 0,0002 | 0,0057 | **Prox1** |
| 17196 | 6718 | 2,5192 | 4,3074 | 4,4983 | 0,0002 | 0,0058 | **Mbp** |
| 235036 | 1671 | 2,4197 | 3,9913 | 4,4887 | 0,0002 | 0,0059 | **Ppan** |
| 20351 | 3449 | 5,6184 | 5,6077 | 4,4850 | 0,0002 | 0,0059 | **Sema4a** |
| 269604 | 4762 | 3,8098 | 3,8098 | 4,4617 | 0,0002 | 0,0062 | **Gpr157** |
| 667952 | 531 | 2,1285 | 3,4407 | 4,4607 | 0,0002 | 0,0062 | **Gm8894** |
| 75623 | 1265 | 6,0498 | 2,6372 | 4,4569 | 0,0002 | 0,0062 | **1700029F09Rik** |
| 75642 | 783 | 7,0537 | -0,7823 | 4,4442 | 0,0002 | 0,0064 | **1700020C07Rik** |
| 67973 | 2202 | 2,5975 | 4,0336 | 4,4402 | 0,0002 | 0,0064 | **Mphosph10** |
| 107568 | 5070 | 2,6514 | 6,2941 | 4,4281 | 0,0003 | 0,0065 | **Wwp1** |
| 108155 | 5384 | 2,1279 | 7,9523 | 4,4281 | 0,0003 | 0,0065 | **Ogt** |
| 55989 | 1976 | 2,3120 | 4,9355 | 4,4140 | 0,0003 | 0,0066 | **Nop58** |
| 226089 | 6282 | 2,1829 | 7,0591 | 4,4069 | 0,0003 | 0,0067 | **C030046E11Rik** |
| 67949 | 1894 | 2,2437 | 4,1899 | 4,3993 | 0,0003 | 0,0068 | **Mki67ip** |
| 52064 | 2001 | 2,0800 | 4,0429 | 4,3977 | 0,0003 | 0,0068 | **Coq5** |
| 75785 | 6683 | 2,4850 | 5,7762 | 4,3910 | 0,0003 | 0,0069 | **Klhl24** |
| 208154 | 3235 | 5,3731 | -0,7194 | 4,3756 | 0,0003 | 0,0070 | **Btla** |
| 74167 | 1334 | 2,6591 | 5,5340 | 4,3746 | 0,0003 | 0,0070 | **Nudt9** |
| 108989 | 7550 | 2,6507 | 9,7702 | 4,3717 | 0,0003 | 0,0070 | **Tpr** |
| 70769 | 3636 | 2,6275 | 4,9224 | 4,3694 | 0,0003 | 0,0071 | **Nolc1** |
| 71520 | 1599 | 2,7363 | 4,7115 | 4,3686 | 0,0003 | 0,0071 | **Grap** |
| 626391 | 2903 | 3,5121 | 2,5243 | 4,3666 | 0,0003 | 0,0071 | **Zfp951** |
| 54712 | 7045 | 2,3159 | 7,3783 | 4,3563 | 0,0003 | 0,0072 | **Plxnc1** |
| 26939 | 4234 | 2,1285 | 4,2595 | 4,3539 | 0,0003 | 0,0072 | **Polr3e** |
| 16184 | 4428 | 3,0126 | 1,7917 | 4,3506 | 0,0003 | 0,0073 | **Il2ra** |
| 232227 | 6812 | 2,5027 | 5,0538 | 4,3225 | 0,0003 | 0,0077 | **Iqsec1** |
| 100217423 | 54 | 6,7745 | -0,8148 | 4,3184 | 0,0003 | 0,0077 | **Snord19** |
| 19044 | 1821 | 2,3559 | 4,3177 | 4,3108 | 0,0003 | 0,0078 | **Ppox** |
| 382038 | 5773 | 2,0293 | 4,6265 | 4,3096 | 0,0003 | 0,0078 | **Urb2** |
| 68310 | 3832 | 2,0989 | 3,5283 | 4,3093 | 0,0003 | 0,0078 | **Zmym1** |
| 72042 | 1639 | 2,0141 | 7,7661 | 4,3074 | 0,0003 | 0,0078 | **Cotl1** |
| 242125 | 3558 | 3,4587 | 3,4424 | 4,3028 | 0,0003 | 0,0079 | **Mab21l3** |
| 20728 | 1266 | 2,0188 | 4,0026 | 4,3013 | 0,0003 | 0,0079 | **Spic** |
| 66690 | 2878 | 3,7042 | 2,3387 | 4,2999 | 0,0003 | 0,0079 | **Tmem186** |
| 17869 | 2399 | 2,8381 | 4,2696 | 4,2875 | 0,0004 | 0,0080 | **Myc** |
| 329910 | 5668 | 7,3278 | 1,1786 | 4,2819 | 0,0004 | 0,0081 | **Acot11** |
| 67187 | 1231 | 3,7874 | 1,7766 | 4,2531 | 0,0004 | 0,0086 | **Zmynd19** |
| 69668 | 1705 | 2,7454 | 3,2619 | 4,2512 | 0,0004 | 0,0086 | **Ccdc115** |
| 66409 | 1839 | 2,1185 | 5,4785 | 4,2425 | 0,0004 | 0,0087 | **Rsl1d1** |
| 214459 | 5330 | 2,0256 | 7,6323 | 4,2106 | 0,0004 | 0,0093 | **Fnbp1l** |
| 107375 | 1833 | 2,8523 | 5,1453 | 4,2076 | 0,0004 | 0,0093 | **Slc25a45** |
| 215193 | 5548 | 3,2064 | 3,0651 | 4,1959 | 0,0004 | 0,0095 | **Diexf** |
| 74522 | 5238 | 2,2915 | 5,5562 | 4,1868 | 0,0004 | 0,0096 | **Morc2a** |
| 18813 | 2432 | 2,2820 | 5,4564 | 4,1831 | 0,0004 | 0,0096 | **Pa2g4** |
| 56306 | 2640 | 2,5803 | 2,4384 | 4,1823 | 0,0004 | 0,0097 | **Fam60a** |
| 20336 | 3723 | 2,0192 | 6,6507 | 4,1768 | 0,0005 | 0,0097 | **Exoc4** |
| 66293 | 1997 | 2,6341 | 2,4752 | 4,1703 | 0,0005 | 0,0099 | **1810032O08Rik** |
| 66942 | 2249 | 2,7383 | 2,9076 | 4,1422 | 0,0005 | 0,0104 | **Ddx18** |
| 12419 | 8986 | 2,1105 | 6,8577 | 4,1205 | 0,0005 | 0,0107 | **Cbx5** |
| 27362 | 1865 | 2,1727 | 4,1454 | 4,1182 | 0,0005 | 0,0108 | **Dnajb9** |
| 71592 | 7372 | 2,0788 | 5,4815 | 4,1126 | 0,0005 | 0,0108 | **Pogk** |
| 12952 | 3016 | 2,0237 | 4,2277 | 4,1094 | 0,0005 | 0,0109 | **Cry1** |
| 216805 | 2852 | 2,3556 | 5,6130 | 4,1003 | 0,0005 | 0,0111 | **Flcn** |
| 66270 | 3346 | 2,4564 | 5,2330 | 4,0833 | 0,0006 | 0,0113 | **Fam134b** |
| 72170 | 1245 | 2,3468 | 3,4589 | 4,0826 | 0,0006 | 0,0113 | **Chchd4** |
| 236727 | 2469 | 2,0431 | 4,2947 | 4,0793 | 0,0006 | 0,0114 | **Slc9a7** |
| 56309 | 1485 | 2,0114 | 4,2479 | 4,0661 | 0,0006 | 0,0116 | **Mycbp** |
| 74868 | 3644 | 2,0815 | 5,0520 | 4,0625 | 0,0006 | 0,0117 | **Tmem65** |
| 665783 | 892 | 2,1642 | 3,1678 | 4,0530 | 0,0006 | 0,0118 | **Gm7785** |
| 117592 | 3213 | 2,4612 | 2,7679 | 4,0315 | 0,0006 | 0,0123 | **B3galt6** |
| 100416706 | 4835 | 2,1271 | 4,0697 | 4,0267 | 0,0006 | 0,0123 | **AA987161** |
| 234728 | 3577 | 2,8105 | 2,7140 | 4,0233 | 0,0007 | 0,0124 | **Ftsjd1** |
| 104662 | 3385 | 2,1020 | 4,6014 | 4,0194 | 0,0007 | 0,0125 | **Tsr1** |
| 56734 | 1924 | 4,7122 | -0,9068 | 4,0182 | 0,0007 | 0,0125 | **Tulp2** |
| 94093 | 8868 | 2,2183 | 5,7436 | 4,0123 | 0,0007 | 0,0126 | **Trim33** |
| 13860 | 4402 | 5,0233 | 2,0570 | 4,0066 | 0,0007 | 0,0127 | **Eps8** |
| 100088 | 2327 | 3,0322 | 3,4935 | 4,0065 | 0,0007 | 0,0127 | **Rcc1** |
| 99003 | 8973 | 3,0061 | 4,6245 | 4,0001 | 0,0007 | 0,0129 | **Qser1** |
| 77577 | 1819 | 3,9981 | 0,6553 | 3,9958 | 0,0007 | 0,0130 | **Spns3** |
| 11937 | 3440 | 2,9947 | 2,7296 | 3,9859 | 0,0007 | 0,0132 | **Atp2a1** |
| 22151 | 1607 | 2,2452 | 5,7071 | 3,9651 | 0,0007 | 0,0136 | **Tubb2a** |
| 243574 | 4703 | 2,8086 | 1,7925 | 3,9561 | 0,0008 | 0,0139 | **Kbtbd8** |
| 54630 | 2376 | 5,4843 | 0,7270 | 3,9464 | 0,0008 | 0,0142 | **Prickle3** |
| 68493 | 3632 | 2,8836 | 2,5399 | 3,9291 | 0,0008 | 0,0146 | **Ndufaf4** |
| 230145 | 2216 | 2,9707 | 1,6382 | 3,9239 | 0,0008 | 0,0148 | **Galnt12** |
| 114716 | 2872 | 2,2495 | 3,6877 | 3,9205 | 0,0008 | 0,0148 | **Spred2** |
| 227522 | 1027 | 2,6332 | 1,4466 | 3,9137 | 0,0008 | 0,0150 | **Rpp38** |
| 100861953 | 436 | 4,5008 | 1,0162 | 3,9131 | 0,0008 | 0,0150 |  |
| 14760 | 2461 | 3,1066 | 1,7858 | 3,9009 | 0,0009 | 0,0154 | **Gpr19** |
| 80891 | 1991 | 3,4248 | 10,2008 | 3,8933 | 0,0009 | 0,0155 | **Fcrls** |
| 233870 | 1777 | 2,3505 | 2,3619 | 3,8919 | 0,0009 | 0,0155 | **Tufm** |
| 65019 | 1062 | 2,1337 | 3,7128 | 3,8917 | 0,0009 | 0,0155 | **Rpl23** |
| 271005 | 1949 | 4,6903 | 0,1840 | 3,8831 | 0,0009 | 0,0157 | **Klhdc1** |
| 229499 | 2042 | 5,4219 | 2,5944 | 3,8822 | 0,0009 | 0,0157 | **Fcrl1** |
| 226265 | 2567 | 4,6170 | 1,6813 | 3,8815 | 0,0009 | 0,0157 | **Eno4** |
| 219249 | 6139 | 2,2787 | 3,8731 | 3,8694 | 0,0009 | 0,0161 | **Tdrd3** |
| 68058 | 2989 | 2,8663 | 2,6997 | 3,8604 | 0,0010 | 0,0163 | **Chd1l** |
| 100040591 | 1349 | 2,7922 | 1,9286 | 3,8486 | 0,0010 | 0,0166 | **Kcnj13** |
| 237926 | 4151 | 3,0527 | 2,7462 | 3,8467 | 0,0010 | 0,0167 | **Rsad1** |
| 22325 | 3482 | 2,3843 | 4,9213 | 3,8373 | 0,0010 | 0,0169 | **Vav2** |
| 19360 | 5153 | 2,5274 | 4,3933 | 3,8351 | 0,0010 | 0,0170 | **Rad50** |
| 100039988 | 632 | 2,1613 | 2,8804 | 3,8348 | 0,0010 | 0,0170 | **Gm11826** |
| 110094 | 5252 | 2,3063 | 5,4030 | 3,8259 | 0,0010 | 0,0172 | **Phka2** |
| 64450 | 2954 | 2,0738 | 4,4250 | 3,8254 | 0,0010 | 0,0172 | **Gpr85** |
| 19933 | 1856 | 2,3518 | 3,6501 | 3,8130 | 0,0011 | 0,0175 | **Rpl21** |
| 18393 | 3371 | 2,0480 | 4,0007 | 3,8124 | 0,0011 | 0,0175 | **Orc2** |
| 20116 | 740 | 2,3387 | 4,2759 | 3,8109 | 0,0011 | 0,0175 | **Rps8** |
| 100019 | 17959 | 2,2875 | 6,8327 | 3,7981 | 0,0011 | 0,0179 | **Mdn1** |
| 53881 | 10913 | 2,2157 | 4,6857 | 3,7918 | 0,0011 | 0,0181 | **Slc5a3** |
| 415115 | 1156 | 7,5967 | 0,4882 | 3,7831 | 0,0011 | 0,0184 | **Neurl2** |
| 16490 | 11582 | 12,5512 | 2,4207 | 3,7680 | 0,0012 | 0,0189 | **Kcna2** |
| 108097 | 4277 | 3,0547 | 3,5235 | 3,7673 | 0,0012 | 0,0189 | **Prkab2** |
| 101612 | 1902 | 2,4127 | 3,4472 | 3,7665 | 0,0012 | 0,0189 | **Grwd1** |
| 67452 | 4406 | 2,1742 | 5,9999 | 3,7604 | 0,0012 | 0,0191 | **Pnpla8** |
| 78267 | 2953 | 2,7946 | 2,1182 | 3,7557 | 0,0012 | 0,0193 | **Klhdc8b** |
| 27998 | 1031 | 3,7099 | 2,6521 | 3,7348 | 0,0013 | 0,0200 | **Exosc5** |
| 100302600 | 48 | 3,9703 | 1,0827 | 3,7328 | 0,0013 | 0,0200 | **Snord43** |
| 11881 | 3989 | 2,0471 | 5,6873 | 3,7248 | 0,0013 | 0,0203 | **Arsb** |
| 50883 | 2247 | 5,7780 | 2,0175 | 3,7247 | 0,0013 | 0,0203 | **Chek2** |
| 383435 | 3624 | 2,2461 | 6,8145 | 3,7176 | 0,0013 | 0,0205 | **Ms4a14** |
| 80880 | 2634 | 2,8475 | 2,3700 | 3,7093 | 0,0014 | 0,0208 | **Kank3** |
| 228536 | 4428 | 2,0159 | 6,3873 | 3,6927 | 0,0014 | 0,0214 | **Bahd1** |
| 246221 | 1522 | 6,2459 | 1,6342 | 3,6889 | 0,0014 | 0,0216 | **Mpst** |
| 11535 | 1381 | 2,4208 | 5,8426 | 3,6856 | 0,0014 | 0,0216 | **Adm** |
| 317717 | 3992 | 2,1543 | 3,1799 | 3,6796 | 0,0015 | 0,0218 | **Sec22a** |
| 118449 | 7116 | 5,7607 | 1,0355 | 3,6729 | 0,0015 | 0,0220 | **Synpo2** |
| 83383 | 2113 | 3,6495 | -0,5228 | 3,6711 | 0,0015 | 0,0221 | **Tcfap4** |
| 217944 | 3987 | 2,7127 | 7,8187 | 3,6516 | 0,0016 | 0,0227 | **Rapgef5** |
| 15983 | 1980 | 2,0300 | 2,8177 | 3,6509 | 0,0016 | 0,0227 | **Ifrd2** |
| 67767 | 1260 | 2,0079 | 3,3486 | 3,6441 | 0,0016 | 0,0229 | **Jagn1** |
| 381062 | 2668 | 2,7001 | 4,1895 | 3,6420 | 0,0016 | 0,0230 | **2210404J11Rik** |
| 70809 | 2613 | 4,3686 | 3,0823 | 3,6357 | 0,0016 | 0,0232 | **Clec2g** |
| 22746 | 2218 | 5,0845 | 0,2140 | 3,6318 | 0,0016 | 0,0233 | **Zfp85-rs1** |
| 70757 | 3603 | 2,3523 | 4,1537 | 3,6284 | 0,0016 | 0,0235 | **Ptplb** |
| 64453 | 4874 | 2,3182 | 2,3486 | 3,6205 | 0,0017 | 0,0238 | **Zfp280b** |
| 67936 | 1498 | 2,4680 | 3,3580 | 3,6198 | 0,0017 | 0,0238 | **Wdr55** |
| 19060 | 2070 | 2,1257 | 4,5286 | 3,6175 | 0,0017 | 0,0239 | **Ppp5c** |
| 110958 | 2051 | 4,6249 | 1,8170 | 3,6018 | 0,0018 | 0,0245 | **D6Mm5e** |
| 52055 | 6124 | 2,2359 | 6,5654 | 3,5969 | 0,0018 | 0,0247 | **Rab11fip5** |
| 239827 | 2184 | 2,4472 | 3,6496 | 3,5960 | 0,0018 | 0,0247 | **Pigz** |
| 244885 | 2759 | 4,4836 | -0,8574 | 3,5956 | 0,0018 | 0,0247 | **Sh2d7** |
| 319545 | 2892 | 4,7084 | 0,0627 | 3,5928 | 0,0018 | 0,0248 | **D430020J02Rik** |
| 70227 | 5964 | 2,1107 | 3,6888 | 3,5921 | 0,0018 | 0,0248 | **Zfp619** |
| 68730 | 1909 | 2,0213 | 4,0973 | 3,5839 | 0,0018 | 0,0251 | **Dus1l** |
| 232187 | 2493 | 2,5280 | 4,6150 | 3,5774 | 0,0019 | 0,0253 | **Smyd5** |
| 67186 | 441 | 2,1553 | 2,9142 | 3,5760 | 0,0019 | 0,0254 | **Rplp2** |
| 70930 | 4242 | 2,5212 | 3,5328 | 3,5746 | 0,0019 | 0,0254 | **Nol8** |
| 215095 | 2377 | 3,9282 | -0,1515 | 3,5720 | 0,0019 | 0,0255 | **Astl** |
| 70873 | 2106 | 4,0802 | 0,3895 | 3,5676 | 0,0019 | 0,0257 | **4921517L17Rik** |
| 67394 | 978 | 2,5962 | 1,4846 | 3,5594 | 0,0019 | 0,0260 | **4930404I05Rik** |
| 100041958 | 1155 | 3,5875 | -0,2963 | 3,5513 | 0,0020 | 0,0265 | **Gm3591** |
| 170826 | 3656 | 3,1545 | 3,0312 | 3,5427 | 0,0020 | 0,0269 | **Ppargc1b** |
| 68121 | 2580 | 2,8778 | 2,6229 | 3,5334 | 0,0021 | 0,0273 | **Cep70** |
| 78697 | 3309 | 3,2446 | 3,0254 | 3,5296 | 0,0021 | 0,0275 | **Pus7** |
| 66824 | 2135 | 2,1622 | 6,5708 | 3,5262 | 0,0021 | 0,0276 | **Pycard** |
| 66756 | 4523 | 2,0945 | 3,3479 | 3,5180 | 0,0021 | 0,0280 | **4933411K20Rik** |
| 74711 | 3121 | 5,2882 | -0,5514 | 3,5152 | 0,0021 | 0,0281 | **Ttll9** |
| 227327 | 2595 | 3,1360 | 3,2537 | 3,5145 | 0,0022 | 0,0281 | **B3gnt7** |
| 75099 | 3383 | 2,0668 | 3,0205 | 3,5104 | 0,0022 | 0,0283 | **Lysmd4** |
| 71603 | 2805 | 2,5513 | 4,2107 | 3,5066 | 0,0022 | 0,0284 | **9130004J05Rik** |
| 72392 | 3109 | 2,0119 | 3,8689 | 3,4987 | 0,0022 | 0,0288 | **Tmem175** |
| 229731 | 3383 | 2,3808 | 4,5064 | 3,4839 | 0,0023 | 0,0293 | **Slc25a24** |
| 232413 | 2222 | 2,0989 | 6,9968 | 3,4791 | 0,0023 | 0,0296 | **Clec12a** |
| 59002 | 1600 | 2,2499 | 3,3478 | 3,4762 | 0,0024 | 0,0297 | **Wdr8** |
| 381066 | 2600 | 3,4132 | 2,4693 | 3,4749 | 0,0024 | 0,0297 | **Zfp948** |
| 100504526 | 714 | 4,2644 | 2,7139 | 3,4747 | 0,0024 | 0,0297 | **Gm20269** |
| 106740 | 1088 | 3,3154 | 3,0675 | 3,4732 | 0,0024 | 0,0297 | **LOC106740** |
| 20017 | 3998 | 3,3324 | 3,2210 | 3,4693 | 0,0024 | 0,0299 | **Polr1b** |
| 69893 | 2876 | 2,4585 | 2,1825 | 3,4657 | 0,0024 | 0,0301 | **2010305A19Rik** |
| 74600 | 872 | 2,1070 | 2,1778 | 3,4591 | 0,0024 | 0,0304 | **Mrpl47** |
| 76265 | 1972 | 3,0855 | 2,3017 | 3,4475 | 0,0025 | 0,0311 | **Tsen54** |
| 11702 | 3206 | 2,0108 | 4,2886 | 3,4416 | 0,0025 | 0,0314 | **Amd1** |
| 333433 | 4393 | 2,3545 | 3,7409 | 3,4376 | 0,0026 | 0,0315 | **Gpd1l** |
| 234515 | 4122 | 2,2460 | 5,2976 | 3,4350 | 0,0026 | 0,0316 | **Inpp4b** |
| 56412 | 2303 | 2,4107 | 2,4612 | 3,4320 | 0,0026 | 0,0317 | **2610024G14Rik** |
| 78895 | 2848 | 2,5813 | 1,9607 | 3,4290 | 0,0026 | 0,0318 | **Pus7l** |
| 104367 | 118 | 2,3014 | 2,2219 | 3,4183 | 0,0027 | 0,0323 | **Snora65** |
| 223267 | 1079 | 3,4048 | 2,0888 | 3,4078 | 0,0028 | 0,0329 | **A2ld1** |
| 100043823 | 3823 | 2,3824 | 2,9421 | 3,4027 | 0,0028 | 0,0332 | **Gm4673** |
| 338350 | 2375 | 3,5876 | 0,5727 | 3,3995 | 0,0028 | 0,0334 | **Acad12** |
| 59001 | 602 | 2,5017 | 2,2182 | 3,3969 | 0,0028 | 0,0336 | **Pole3** |
| 14071 | 2733 | 3,3758 | 1,5782 | 3,3954 | 0,0028 | 0,0337 | **F9** |
| 22282 | 2419 | 2,4620 | 3,9386 | 3,3893 | 0,0029 | 0,0338 | **Usf2** |
| 210503 | 2912 | 3,0284 | 1,4968 | 3,3843 | 0,0029 | 0,0341 | **Zfp677** |
| 107566 | 2030 | 2,3441 | 3,3854 | 3,3823 | 0,0029 | 0,0342 | **Arl2bp** |
| 212670 | 2247 | 3,7925 | 0,4680 | 3,3813 | 0,0029 | 0,0343 | **Catsper2** |
| 78255 | 7133 | 3,2481 | 4,0696 | 3,3709 | 0,0030 | 0,0350 | **Ralgps2** |
| 71041 | 2098 | 3,9353 | 1,3951 | 3,3588 | 0,0031 | 0,0355 | **Pcgf6** |
| 100503433 | 891 | 3,6744 | 0,2428 | 3,3458 | 0,0032 | 0,0363 | **Gm15433** |
| 19219 | 2966 | 3,2940 | 5,9195 | 3,3457 | 0,0032 | 0,0363 | **Ptger4** |
| 68080 | 1404 | 3,6551 | 1,1501 | 3,3418 | 0,0032 | 0,0365 | **Gpn3** |
| 72326 | 1898 | 2,9804 | 0,6691 | 3,3413 | 0,0032 | 0,0365 | **2500004C02Rik** |
| 67880 | 918 | 2,1579 | 4,1253 | 3,3319 | 0,0033 | 0,0371 | **Dcxr** |
| 67155 | 6086 | 2,0672 | 4,2759 | 3,3316 | 0,0033 | 0,0371 | **Smarca2** |
| 66973 | 1053 | 2,2966 | 2,3092 | 3,3201 | 0,0034 | 0,0379 | **Mrps18b** |
| 72147 | 3336 | 2,9364 | 2,4981 | 3,3200 | 0,0034 | 0,0379 | **Zbtb46** |
| 71131 | 3621 | 4,4166 | 0,2529 | 3,3163 | 0,0034 | 0,0381 | **Zfp689** |
| 15216 | 1722 | 2,0080 | 4,6020 | 3,3147 | 0,0034 | 0,0381 | **Hfe** |
| 100042533 | 791 | 3,2471 | -2,0924 | 3,3036 | 0,0035 | 0,0389 | **Gm11578** |
| 12509 | 1811 | 3,4713 | 1,1347 | 3,3033 | 0,0035 | 0,0389 | **Cd59a** |
| 278097 | 2034 | 2,4464 | 3,5174 | 3,3013 | 0,0035 | 0,0390 | **Armcx6** |
| 100504235 | 3401 | 2,6939 | 0,7974 | 3,2969 | 0,0036 | 0,0393 | **Gm20125** |
| 66938 | 2328 | 2,5772 | 3,3879 | 3,2915 | 0,0036 | 0,0395 | **1700029G01Rik** |
| 68034 | 1696 | 2,3878 | 2,4849 | 3,2871 | 0,0036 | 0,0397 | **Fam122a** |
| 73736 | 716 | 2,0561 | 2,9926 | 3,2857 | 0,0037 | 0,0398 | **Fcf1** |
| 103784 | 3431 | 2,2272 | 2,2855 | 3,2788 | 0,0037 | 0,0402 | **Wdr92** |
| 66193 | 1538 | 2,0423 | 3,7786 | 3,2787 | 0,0037 | 0,0402 | **1110049F12Rik** |
| 67492 | 2536 | 5,3232 | 0,0030 | 3,2753 | 0,0037 | 0,0404 | **Anubl1** |
| 93709 | 4537 | 4,2553 | 0,5179 | 3,2743 | 0,0038 | 0,0405 | **Pcdhga1** |
| 208146 | 7344 | 2,0987 | 4,4340 | 3,2686 | 0,0038 | 0,0409 | **Yeats2** |
| 110606 | 2560 | 3,3353 | 1,7790 | 3,2673 | 0,0038 | 0,0410 | **Fntb** |
| 381406 | 1868 | 2,0232 | 2,9717 | 3,2645 | 0,0038 | 0,0411 | **2810408M09Rik** |
| 78416 | 1072 | 3,6495 | 0,8229 | 3,2545 | 0,0039 | 0,0417 | **Rnase6** |
| 104681 | 4509 | 3,0856 | 4,4864 | 3,2492 | 0,0040 | 0,0422 | **Slc16a6** |
| 106582 | 1464 | 2,6662 | 2,3921 | 3,2465 | 0,0040 | 0,0423 | **Nrm** |
| 103232 | 462 | 3,0563 | 1,1681 | 3,2451 | 0,0040 | 0,0424 | **AI462171** |
| 72133 | 6395 | 3,8141 | 1,8948 | 3,2414 | 0,0040 | 0,0427 | **Trub1** |
| 66406 | 1374 | 2,5699 | 2,2071 | 3,2385 | 0,0041 | 0,0429 | **Sac3d1** |
| 70683 | 8831 | 2,1478 | 5,6669 | 3,2329 | 0,0041 | 0,0432 | **Utp20** |
| 268481 | 2735 | 2,7912 | 0,6951 | 3,2277 | 0,0042 | 0,0436 | **Krt222** |
| 22592 | 4010 | 2,6898 | 3,4182 | 3,2260 | 0,0042 | 0,0437 | **Ercc5** |
| 66171 | 883 | 4,0147 | 0,9032 | 3,2257 | 0,0042 | 0,0437 | **Pgls** |
| 17345 | 10075 | 2,7233 | 5,1436 | 3,2193 | 0,0043 | 0,0441 | **Mki67** |
| 214968 | 6541 | 6,4738 | 3,9929 | 3,2149 | 0,0043 | 0,0443 | **Sema6d** |
| 320466 | 2125 | 4,1902 | 0,2956 | 3,2148 | 0,0043 | 0,0443 | **A230103J11Rik** |
| 239743 | 2405 | 9,1349 | 3,6102 | 3,2134 | 0,0043 | 0,0444 | **Klhl6** |
| 207474 | 4795 | 3,6242 | 2,1941 | 3,2081 | 0,0044 | 0,0448 | **Kctd12b** |
| 72475 | 3195 | 2,3474 | 2,6727 | 3,2080 | 0,0044 | 0,0448 | **Ssbp3** |
| 66094 | 468 | 3,3412 | -1,3229 | 3,2065 | 0,0044 | 0,0449 | **Lsm7** |
| 320054 | 2097 | 3,7408 | -0,1131 | 3,1988 | 0,0045 | 0,0455 | **9230116N13Rik** |
| 81003 | 4258 | 2,1733 | 3,7573 | 3,1930 | 0,0045 | 0,0458 | **Trim23** |
| 27015 | 4226 | 3,2433 | 3,8785 | 3,1927 | 0,0045 | 0,0458 | **Polk** |
| 213760 | 3837 | 2,3394 | 2,9834 | 3,1926 | 0,0045 | 0,0458 | **Prepl** |
| 16160 | 1951 | 2,9119 | 6,8255 | 3,1903 | 0,0045 | 0,0460 | **Il12b** |
| 70456 | 854 | 2,0861 | 3,1463 | 3,1900 | 0,0046 | 0,0460 | **Brp44** |
| 240261 | 2801 | 4,7518 | 0,5799 | 3,1869 | 0,0046 | 0,0462 | **Ccdc112** |
| 71971 | 2701 | 2,7591 | 2,0281 | 3,1860 | 0,0046 | 0,0462 | **Zswim1** |
| 76429 | 1607 | 2,5201 | 1,5597 | 3,1809 | 0,0046 | 0,0467 | **Lhpp** |
| 67223 | 1213 | 2,0252 | 3,0683 | 3,1763 | 0,0047 | 0,0470 | **Rrp15** |
| 104923 | 1676 | 2,8017 | 2,2475 | 3,1739 | 0,0047 | 0,0472 | **Adi1** |
| 66775 | 2089 | 2,0166 | 5,0176 | 3,1654 | 0,0048 | 0,0477 | **Ptplad2** |
| 16506 | 1956 | 4,6928 | 0,1669 | 3,1624 | 0,0048 | 0,0480 | **Kcnd1** |
| 29873 | 3871 | 3,4204 | 1,8051 | 3,1592 | 0,0049 | 0,0482 | **Cspg5** |
| 68108 | 3172 | 2,0730 | 3,7141 | 3,1578 | 0,0049 | 0,0483 | **9430008C03Rik** |
| 101867 | 3177 | 2,0728 | 3,7333 | 3,1562 | 0,0049 | 0,0484 | **Rrp8** |
| 13436 | 4322 | 3,2836 | 0,3139 | 3,1559 | 0,0049 | 0,0484 | **Dnmt3b** |
| 433813 | 1229 | 2,5343 | 1,6483 | 3,1554 | 0,0049 | 0,0485 | **Pusl1** |
| 106248 | 2829 | 2,2426 | 2,7280 | 3,1537 | 0,0049 | 0,0486 | **Qtrtd1** |
| 81896 | 4067 | 2,4566 | 3,0802 | 3,1503 | 0,0050 | 0,0488 | **Ift122** |
| 227326 | 2550 | 3,3716 | -0,7216 | 3,1478 | 0,0050 | 0,0490 | **Gpr55** |
| 70573 | 2702 | 2,2216 | 2,7873 | 3,1458 | 0,0050 | 0,0491 | **Tbccd1** |
| 381319 | 712 | 2,8693 | 2,3155 | 3,1449 | 0,0050 | 0,0491 | **Batf3** |
| 242864 | 3673 | 3,4950 | 2,3277 | 3,1426 | 0,0051 | 0,0493 | **Napepld** |
| 105193 | 2294 | 4,1187 | -0,7141 | 3,1382 | 0,0051 | 0,0496 | **Nhlrc1** |
| 231633 | 2084 | 4,3226 | 1,9734 | 3,1373 | 0,0051 | 0,0497 | **Tmem119** |
| 21885 | 4369 | 2,4754 | 3,5794 | 3,1364 | 0,0051 | 0,0498 | **Tle1** |
