# Supplementary material for: Myeloid C/EBPβ deficiency reshapes microglial gene expression and is protective in experimental autoimmune encephalomyelitis
Source: J Neuroinflammation. 2017 Mar 16;14:54. doi: 10.1186/s12974-017-0834-5 (PMC5356255; doi:10.1186/s12974-017-0834-5)
Supplement: Additional file 1: Tables S1-S6. — List the genes significantly up-regulated (tables 1, 3 and 5) or down-regulated (tables 2, 4 and 6) by the absence of C/EBPβ in control (tables 1, 2), LPS-treated (tables 3, 4) and LPS+IFNγ-treated (tables 5, 6) primary microglial cultures. These data were obtained by RNAseq as described in Methods. (ZIP 253 kb) [file 12974_2017_834_MOESM1_ESM.zip › 12974_2017_834_MOESM1_ESM/Table S4.docx]

| **Table S4** |
| --- |
| **Genes with significantly decreased expression in LysMCre-CEBPbetafl/fl microglia in LPS condition** |

| **GeneID** | **Length** | **FC** | **AveExpr** | **t** | **P.Value** | **adj.P** | **gene_symbol** |
| --- | --- | --- | --- | --- | --- | --- | --- |
| 56619 | 2519 | 0,0298 | 4,9510 | -15,6810 | 8,63E-13 | 1,31E-08 | **Clec4e** |
| 17105 | 1057 | 0,0019 | 7,2768 | -13,4471 | 1,49E-11 | 7,57E-08 | **Lyz2** |
| 20210 | 531 | 0,0391 | 6,7641 | -13,6407 | 1,15E-11 | 7,57E-08 | **Saa3** |
| 270084 | 2800 | 0,2269 | 7,3229 | -13,0072 | 2,73E-11 | 1,04E-07 | **Lpcat2** |
| 80719 | 2133 | 0,1292 | 6,3009 | -12,3820 | 6,66E-11 | 1,53E-07 | **Igsf6** |
| 56738 | 2644 | 0,1767 | 4,4779 | -12,3249 | 7,24E-11 | 1,53E-07 | **Mocs1** |
| 70097 | 7183 | 0,2836 | 8,2794 | -12,2910 | 7,61E-11 | 1,53E-07 | **Sash1** |
| 17158 | 6078 | 0,3143 | 8,0449 | -12,2556 | 8,01E-11 | 1,53E-07 | **Man2a1** |
| 215772 | 5218 | 0,1460 | 4,8739 | -12,1454 | 9,42E-11 | 1,53E-07 | **9130014G24Rik** |
| 14081 | 3891 | 0,2228 | 6,2145 | -12,1026 | 1,00E-10 | 1,53E-07 | **Acsl1** |
| 231805 | 2588 | 0,0980 | 4,2906 | -11,9532 | 1,25E-10 | 1,73E-07 | **Pilra** |
| 12985 | 1363 | 0,0249 | 0,9178 | -11,3386 | 3,19E-10 | 3,34E-07 | **Csf3** |
| 16656 | 9021 | 0,1755 | 4,3214 | -11,3202 | 3,29E-10 | 3,34E-07 | **Hivep3** |
| 100043424 | 4492 | 0,1655 | 3,0493 | -11,2488 | 3,67E-10 | 3,36E-07 | **Gm14005** |
| 98496 | 2667 | 0,2656 | 6,8526 | -11,2356 | 3,75E-10 | 3,36E-07 | **Pid1** |
| 67338 | 4242 | 0,1441 | 4,3970 | -10,8061 | 7,41E-10 | 6,27E-07 | **Rffl** |
| 16449 | 5493 | 0,1266 | 4,4196 | -10,7319 | 8,35E-10 | 6,36E-07 | **Jag1** |
| 19267 | 5411 | 0,2299 | 5,7530 | -10,7485 | 8,13E-10 | 6,36E-07 | **Ptpre** |
| 73914 | 2969 | 0,1145 | 5,2863 | -10,6154 | 1,01E-09 | 7,32E-07 | **Irak3** |
| 76220 | 4292 | 0,2088 | 2,8378 | -10,3697 | 1,51E-09 | 1,00E-06 | **6530402F18Rik** |
| 243197 | 2508 | 0,2136 | 3,4676 | -10,3925 | 1,45E-09 | 1,00E-06 | **Mfsd7a** |
| 11610 | 4029 | 0,3548 | 4,9574 | -10,3062 | 1,68E-09 | 1,06E-06 | **Agtrap** |
| 109934 | 5801 | 0,4718 | 8,0902 | -10,2495 | 1,84E-09 | 1,12E-06 | **Abr** |
| 19201 | 2262 | 0,2002 | 3,6770 | -10,0384 | 2,63E-09 | 1,43E-06 | **Pstpip2** |
| 22436 | 4587 | 0,2056 | 5,7895 | -9,8584 | 3,58E-09 | 1,82E-06 | **Xdh** |
| 74243 | 3111 | 0,1802 | 3,1433 | -9,5705 | 5,89E-09 | 2,89E-06 | **2210009G21Rik** |
| 245126 | 957 | 0,0186 | 1,5214 | -9,3946 | 8,02E-09 | 3,70E-06 | **Tarm1** |
| 64292 | 3647 | 0,0433 | 5,7219 | -9,2924 | 9,62E-09 | 4,19E-06 | **Ptges** |
| 228608 | 2262 | 0,3304 | 5,8778 | -9,2454 | 1,05E-08 | 4,29E-06 | **Smox** |
| 171171 | 1770 | 0,1403 | 1,6140 | -9,2016 | 1,13E-08 | 4,31E-06 | **Ntng2** |
| 241075 | 8562 | 0,2998 | 6,3655 | -9,2056 | 1,12E-08 | 4,31E-06 | **Plekhm3** |
| 26464 | 1811 | 0,2777 | 5,2585 | -9,0809 | 1,41E-08 | 5,10E-06 | **Vnn3** |
| 72472 | 6556 | 0,3084 | 5,9591 | -9,0137 | 1,59E-08 | 5,63E-06 | **Slc16a10** |
| 12489 | 2571 | 0,1279 | 4,7300 | -8,9673 | 1,73E-08 | 5,98E-06 | **Cd33** |
| 12494 | 2995 | 0,0432 | 5,5751 | -8,8602 | 2,10E-08 | 6,96E-06 | **Cd38** |
| 211770 | 4026 | 0,3084 | 4,9829 | -8,8615 | 2,10E-08 | 6,96E-06 | **Trib1** |
| 29863 | 4127 | 0,1811 | 4,1108 | -8,7484 | 2,58E-08 | 8,37E-06 | **Pde7b** |
| 100604 | 2829 | 0,1168 | 3,6460 | -8,6687 | 3,00E-08 | 9,13E-06 | **Lrrc8c** |
| 26570 | 9227 | 0,1682 | 6,8229 | -8,6089 | 3,35E-08 | 9,81E-06 | **Slc7a11** |
| 67712 | 4162 | 0,1929 | 5,0455 | -8,4861 | 4,22E-08 | 1,21E-05 | **Slc25a37** |
| 16911 | 2049 | 0,2366 | 5,2507 | -8,3291 | 5,68E-08 | 1,54E-05 | **Lmo4** |
| 83430 | 1359 | 0,0619 | 0,1905 | -8,2662 | 6,41E-08 | 1,59E-05 | **Il23a** |
| 170741 | 1020 | 0,1845 | 2,9790 | -8,2520 | 6,58E-08 | 1,59E-05 | **Pilrb1** |
| 18126 | 3990 | 0,2710 | 6,1173 | -8,2938 | 6,08E-08 | 1,59E-05 | **Nos2** |
| 269823 | 1784 | 0,2971 | 4,7309 | -8,2701 | 6,36E-08 | 1,59E-05 | **Pon3** |
| 71712 | 2807 | 0,3456 | 5,1868 | -8,1761 | 7,61E-08 | 1,81E-05 | **Dram1** |
| 19730 | 4020 | 0,1195 | 6,1425 | -8,1337 | 8,26E-08 | 1,85E-05 | **Ralgds** |
| 545812 | 1807 | 0,1402 | 2,7641 | -8,1363 | 8,22E-08 | 1,85E-05 | **Pilrb2** |
| 67844 | 2054 | 0,4254 | 6,2952 | -8,1376 | 8,20E-08 | 1,85E-05 | **Rab32** |
| 12608 | 1504 | 0,0287 | 3,4337 | -8,0352 | 1,00E-07 | 2,15E-05 | **Cebpb** |
| 20656 | 3824 | 0,2616 | 8,8128 | -8,0407 | 9,89E-08 | 2,15E-05 | **Sod2** |
| 18550 | 4399 | 0,3973 | 7,5756 | -8,0264 | 1,02E-07 | 2,15E-05 | **Furin** |
| 50934 | 4034 | 0,3186 | 6,9593 | -8,0113 | 1,05E-07 | 2,19E-05 | **Slc7a8** |
| 212307 | 4333 | 0,4457 | 5,6045 | -7,9641 | 1,15E-07 | 2,33E-05 | **Mapre2** |
| 210104 | 3381 | 0,1915 | 2,7541 | -7,9502 | 1,18E-07 | 2,33E-05 | **Zfp658** |
| 381560 | 4238 | 0,3727 | 4,9124 | -7,9523 | 1,18E-07 | 2,33E-05 | **Xkr8** |
| 212167 | 3745 | 0,4861 | 7,2526 | -7,8364 | 1,48E-07 | 2,88E-05 | **Pion** |
| 19206 | 4305 | 0,2418 | 3,9018 | -7,7890 | 1,62E-07 | 3,08E-05 | **Ptch1** |
| 83925 | 10204 | 0,3460 | 7,2491 | -7,7639 | 1,70E-07 | 3,16E-05 | **Trps1** |
| 15975 | 7014 | 0,4610 | 7,5657 | -7,6695 | 2,05E-07 | 3,68E-05 | **Ifnar1** |
| 58217 | 3006 | 0,0350 | -0,0357 | -7,6473 | 2,15E-07 | 3,73E-05 | **Trem1** |
| 80879 | 2577 | 0,2931 | 6,2512 | -7,6404 | 2,18E-07 | 3,73E-05 | **Slc16a3** |
| 69543 | 1015 | 0,0782 | 1,9843 | -7,6073 | 2,33E-07 | 3,94E-05 | **Capns2** |
| 329739 | 5702 | 0,3705 | 7,0030 | -7,5615 | 2,55E-07 | 4,27E-05 | **Fam102b** |
| 100037283 | 962 | 0,2439 | 4,5993 | -7,5461 | 2,63E-07 | 4,32E-05 | **Rnaset2a** |
| 20558 | 3958 | 0,1290 | 3,6742 | -7,5233 | 2,75E-07 | 4,46E-05 | **Slfn4** |
| 100504452 | 3947 | 0,2287 | 4,7986 | -7,5090 | 2,83E-07 | 4,51E-05 | **Gm20235** |
| 21664 | 1953 | 0,2393 | 3,7940 | -7,4124 | 3,44E-07 | 5,19E-05 | **Phlda1** |
| 110796 | 5814 | 0,3504 | 3,9480 | -7,3921 | 3,59E-07 | 5,31E-05 | **Tshz1** |
| 224794 | 4558 | 0,4413 | 5,5795 | -7,3928 | 3,58E-07 | 5,31E-05 | **Enpp4** |
| 72747 | 2385 | 0,4352 | 4,3885 | -7,3562 | 3,86E-07 | 5,60E-05 | **Ttc39c** |
| 231991 | 2873 | 0,4186 | 5,1259 | -7,3334 | 4,04E-07 | 5,76E-05 | **Creb5** |
| 12986 | 4047 | 0,3462 | 6,8791 | -7,2732 | 4,57E-07 | 6,28E-05 | **Csf3r** |
| 171212 | 4702 | 0,3370 | 4,3441 | -7,2329 | 4,97E-07 | 6,75E-05 | **Galnt10** |
| 75019 | 1665 | 0,1002 | -1,5389 | -7,1854 | 5,48E-07 | 7,38E-05 | **Rnase10** |
| 170625 | 4447 | 0,3319 | 6,2915 | -7,1149 | 6,33E-07 | 8,29E-05 | **Snx18** |
| 80889 | 3847 | 0,3980 | 5,9206 | -7,0688 | 6,97E-07 | 8,84E-05 | **Mesdc1** |
| 17394 | 2453 | 0,0321 | -0,1502 | -7,0486 | 7,27E-07 | 9,00E-05 | **Mmp8** |
| 56613 | 3106 | 0,4538 | 6,0882 | -7,0513 | 7,23E-07 | 9,00E-05 | **Rps6ka4** |
| 14362 | 4378 | 0,0942 | 4,3520 | -7,0405 | 7,39E-07 | 9,02E-05 | **Fzd1** |
| 75007 | 2834 | 0,4011 | 5,3195 | -6,7983 | 1,23E-06 | 0,0001 | **Fam63a** |
| 269181 | 7054 | 0,4895 | 7,3592 | -6,7976 | 1,23E-06 | 0,0001 | **Mgat4a** |
| 73504 | 2504 | 0,0696 | 0,8583 | -6,7508 | 1,36E-06 | 0,0001 | **1700071M16Rik** |
| 11988 | 7857 | 0,1690 | 7,3121 | -6,7229 | 1,44E-06 | 0,0002 | **Slc7a2** |
| 14747 | 2845 | 0,0899 | 4,1051 | -6,6941 | 1,53E-06 | 0,0002 | **Cmklr1** |
| 23921 | 2848 | 0,2386 | 2,4702 | -6,6713 | 1,61E-06 | 0,0002 | **Sh2b2** |
| 17474 | 1336 | 0,2642 | 8,3203 | -6,6595 | 1,65E-06 | 0,0002 | **Clec4d** |
| 51791 | 2356 | 0,2111 | 2,8171 | -6,6151 | 1,81E-06 | 0,0002 | **Rgs14** |
| 54683 | 688 | 0,3694 | 8,7332 | -6,5577 | 2,05E-06 | 0,0002 | **Prdx5** |
| 68195 | 1013 | 0,2003 | 3,9348 | -6,5264 | 2,19E-06 | 0,0002 | **Rnaset2b** |
| 100861753 | 1669 | 0,2416 | 3,2949 | -6,5270 | 2,19E-06 | 0,0002 |  |
| 26569 | 4008 | 0,3919 | 4,6503 | -6,5252 | 2,19E-06 | 0,0002 | **Slc27a4** |
| 70296 | 3470 | 0,4930 | 7,4662 | -6,5322 | 2,16E-06 | 0,0002 | **Tbc1d13** |
| 269717 | 3851 | 0,3495 | 5,0424 | -6,5168 | 2,23E-06 | 0,0002 | **Orai2** |
| 20288 | 3998 | 0,3814 | 8,2773 | -6,5098 | 2,27E-06 | 0,0002 | **Msr1** |
| 78317 | 4959 | 0,4747 | 6,7168 | -6,5003 | 2,31E-06 | 0,0002 | **Ccdc88b** |
| 619308 | 2893 | 0,2164 | 1,9003 | -6,4183 | 2,76E-06 | 0,0003 | **F830208F22Rik** |
| 12475 | 1497 | 0,4710 | 9,6500 | -6,2748 | 3,77E-06 | 0,0003 | **Cd14** |
| 225028 | 4231 | 0,4011 | 5,8142 | -6,2401 | 4,07E-06 | 0,0004 | **Map4k3** |
| 432466 | 1550 | 0,3493 | 4,4396 | -6,2269 | 4,19E-06 | 0,0004 | **Gm5424** |
| 12642 | 1353 | 0,1782 | 6,4143 | -6,2074 | 4,37E-06 | 0,0004 | **Ch25h** |
| 18641 | 3719 | 0,4464 | 7,2345 | -6,1744 | 4,70E-06 | 0,0004 | **Pfkl** |
| 170744 | 3158 | 0,3461 | 6,3420 | -6,1351 | 5,12E-06 | 0,0004 | **Tlr8** |
| 17381 | 3607 | 0,3683 | 6,3779 | -6,1251 | 5,23E-06 | 0,0004 | **Mmp12** |
| 19663 | 2780 | 0,3627 | 3,0620 | -6,1043 | 5,48E-06 | 0,0005 | **Rbpms** |
| 15361 | 2046 | 0,3901 | 5,6884 | -6,1063 | 5,45E-06 | 0,0005 | **Hmga1** |
| 57425 | 522 | 0,0447 | -0,4715 | -6,0841 | 5,72E-06 | 0,0005 | **U90926** |
| 69573 | 1106 | 0,2250 | 4,0754 | -6,0509 | 6,16E-06 | 0,0005 | **2310016C08Rik** |
| 20339 | 2912 | 0,0644 | -1,1375 | -6,0325 | 6,41E-06 | 0,0005 | **Sele** |
| 257632 | 4621 | 0,3657 | 5,4181 | -5,9866 | 7,10E-06 | 0,0005 | **Nod2** |
| 23986 | 1610 | 0,3615 | 4,9399 | -5,9667 | 7,42E-06 | 0,0006 | **Eci2** |
| 71371 | 4494 | 0,3771 | 6,8230 | -5,9689 | 7,38E-06 | 0,0006 | **Arid5b** |
| 77633 | 2755 | 0,4278 | 3,6254 | -5,9667 | 7,42E-06 | 0,0006 | **4930594C11Rik** |
| 15251 | 4761 | 0,3953 | 8,7240 | -5,9548 | 7,62E-06 | 0,0006 | **Hif1a** |
| 16518 | 5448 | 0,2376 | 2,8187 | -5,9047 | 8,51E-06 | 0,0006 | **Kcnj2** |
| 67765 | 1494 | 0,1472 | 2,0626 | -5,8805 | 8,99E-06 | 0,0006 | **5830432E09Rik** |
| 12983 | 4765 | 0,2582 | 8,0383 | -5,8428 | 9,77E-06 | 0,0007 | **Csf2rb** |
| 225372 | 2062 | 0,2686 | 2,9299 | -5,8378 | 9,88E-06 | 0,0007 | **Apbb3** |
| 106952 | 5499 | 0,4199 | 5,1201 | -5,8349 | 9,95E-06 | 0,0007 | **Arap3** |
| 11770 | 642 | 0,0785 | 2,2850 | -5,8105 | 1,05E-05 | 0,0007 | **Fabp4** |
| 353310 | 3305 | 0,3013 | 3,8356 | -5,7382 | 1,23E-05 | 0,0008 | **Zfp703** |
| 14962 | 2747 | 0,3631 | 5,2919 | -5,7368 | 1,24E-05 | 0,0008 | **Cfb** |
| 382034 | 6953 | 0,4435 | 5,4204 | -5,7306 | 1,26E-05 | 0,0008 | **Gse1** |
| 11303 | 10260 | 0,3650 | 9,2102 | -5,7234 | 1,28E-05 | 0,0008 | **Abca1** |
| 21462 | 2065 | 0,1404 | -0,1102 | -5,7056 | 1,33E-05 | 0,0008 | **Tcp10c** |
| 26408 | 5393 | 0,3199 | 3,8071 | -5,6994 | 1,35E-05 | 0,0008 | **Map3k5** |
| 243813 | 1690 | 0,3315 | 2,9388 | -5,6814 | 1,40E-05 | 0,0009 | **Leng9** |
| 54199 | 2110 | 0,4699 | 7,0296 | -5,6392 | 1,54E-05 | 0,0009 | **Ccrl2** |
| 16658 | 3389 | 0,2611 | 5,5809 | -5,6285 | 1,58E-05 | 0,0009 | **Mafb** |
| 17937 | 2575 | 0,4119 | 5,4489 | -5,6231 | 1,60E-05 | 0,0009 | **Nab2** |
| 66597 | 1699 | 0,3882 | 3,1592 | -5,5867 | 1,74E-05 | 0,0010 | **Trim13** |
| 11847 | 1417 | 0,1780 | 2,0678 | -5,5771 | 1,77E-05 | 0,0010 | **Arg2** |
| 15024 | 2897 | 0,4728 | 3,6464 | -5,5517 | 1,88E-05 | 0,0010 | **H2-T10** |
| 57248 | 878 | 0,1125 | 0,3387 | -5,5385 | 1,94E-05 | 0,0011 | **Ly6i** |
| 20557 | 1816 | 0,3983 | 3,6204 | -5,5286 | 1,98E-05 | 0,0011 | **Slfn3** |
| 19419 | 5105 | 0,1818 | 1,4811 | -5,5191 | 2,02E-05 | 0,0011 | **Rasgrp1** |
| 353346 | 3433 | 0,0554 | -0,9769 | -5,4988 | 2,12E-05 | 0,0011 | **Gpr141** |
| 67603 | 2797 | 0,3046 | 5,0940 | -5,4809 | 2,21E-05 | 0,0012 | **Dusp6** |
| 667034 | 1223 | 0,0808 | -0,4707 | -5,4719 | 2,25E-05 | 0,0012 | **Pnp2** |
| 12894 | 4301 | 0,3882 | 6,3916 | -5,4713 | 2,26E-05 | 0,0012 | **Cpt1a** |
| 320404 | 5101 | 0,4195 | 7,5814 | -5,4572 | 2,33E-05 | 0,0012 | **Itpkb** |
| 66205 | 1257 | 0,4573 | 4,6004 | -5,4575 | 2,33E-05 | 0,0012 | **Cd302** |
| 14130 | 1547 | 0,3040 | 7,5706 | -5,4458 | 2,39E-05 | 0,0012 | **Fcgr2b** |
| 20343 | 2321 | 0,1404 | -1,2092 | -5,4281 | 2,49E-05 | 0,0013 | **Sell** |
| 11541 | 1844 | 0,2530 | 2,1233 | -5,4120 | 2,58E-05 | 0,0013 | **Adora2b** |
| 75767 | 7965 | 0,3117 | 5,6042 | -5,4052 | 2,62E-05 | 0,0013 | **Rab11fip1** |
| 104215 | 4122 | 0,4008 | 5,5028 | -5,3962 | 2,68E-05 | 0,0013 | **Rhoq** |
| 23796 | 3564 | 0,1107 | -1,5888 | -5,3458 | 3,00E-05 | 0,0015 | **Aplnr** |
| 330122 | 1013 | 0,0755 | 2,5779 | -5,3359 | 3,07E-05 | 0,0015 | **Cxcl3** |
| 319565 | 21718 | 0,4537 | 5,9059 | -5,2517 | 3,72E-05 | 0,0017 | **Syne2** |
| 219140 | 7517 | 0,2906 | 5,7724 | -5,2490 | 3,75E-05 | 0,0018 | **Spata13** |
| 17386 | 2675 | 0,2263 | 8,9364 | -5,2303 | 3,91E-05 | 0,0018 | **Mmp13** |
| 15950 | 3793 | 0,4817 | 7,1177 | -5,2264 | 3,94E-05 | 0,0018 | **Ifi203** |
| 66648 | 6807 | 0,3958 | 3,3495 | -5,1814 | 4,37E-05 | 0,0019 | **5730494M16Rik** |
| 70948 | 2228 | 0,0852 | -0,4832 | -5,1575 | 4,62E-05 | 0,0020 | **Wdr20b** |
| 399558 | 7099 | 0,2282 | 5,1141 | -5,0773 | 5,56E-05 | 0,0023 | **Flrt2** |
| 20354 | 4377 | 0,3624 | 7,8991 | -5,0672 | 5,69E-05 | 0,0023 | **Sema4d** |
| 228071 | 8900 | 0,4419 | 5,4918 | -5,0574 | 5,82E-05 | 0,0024 | **Sestd1** |
| 100340 | 1925 | 0,3481 | 3,1245 | -5,0557 | 5,84E-05 | 0,0024 | **Smpdl3b** |
| 12176 | 1756 | 0,3757 | 4,6152 | -5,0452 | 5,99E-05 | 0,0024 | **Bnip3** |
| 207728 | 4641 | 0,3944 | 2,3698 | -5,0372 | 6,10E-05 | 0,0024 | **Pde2a** |
| 12226 | 5010 | 0,3815 | 7,5735 | -5,0343 | 6,14E-05 | 0,0024 | **Btg1** |
| 13132 | 4631 | 0,4399 | 8,0053 | -5,0289 | 6,22E-05 | 0,0025 | **Dab2** |
| 76969 | 2682 | 0,1665 | -0,1373 | -5,0090 | 6,51E-05 | 0,0026 | **Chst1** |
| 100861977 | 1664 | 0,2087 | 1,6212 | -4,9885 | 6,83E-05 | 0,0026 |  |
| 71720 | 6935 | 0,2855 | 3,8774 | -4,9796 | 6,97E-05 | 0,0027 | **Osbpl3** |
| 14538 | 7039 | 0,4371 | 4,4936 | -4,9750 | 7,04E-05 | 0,0027 | **Gcnt2** |
| 207521 | 5770 | 0,4685 | 5,8512 | -4,9316 | 7,79E-05 | 0,0029 | **Dtx4** |
| 71082 | 1520 | 0,2573 | 0,1368 | -4,9234 | 7,94E-05 | 0,0029 | **4933416M07Rik** |
| 23971 | 2600 | 0,4168 | 4,5466 | -4,9184 | 8,03E-05 | 0,0029 | **Papss1** |
| 15937 | 1090 | 0,3895 | 5,7995 | -4,9037 | 8,31E-05 | 0,0030 | **Ier3** |
| 15275 | 4509 | 0,3930 | 5,3828 | -4,8685 | 9,02E-05 | 0,0032 | **Hk1** |
| 93691 | 1344 | 0,4773 | 3,8468 | -4,8644 | 9,10E-05 | 0,0032 | **Klf7** |
| 238330 | 4112 | 0,2387 | 2,9810 | -4,8574 | 9,25E-05 | 0,0032 | **6430527G18Rik** |
| 100503468 | 1781 | 0,2563 | 2,4045 | -4,8508 | 9,39E-05 | 0,0033 | **Gm14023** |
| 110751 | 3165 | 0,2579 | 2,6802 | -4,8378 | 9,68E-05 | 0,0033 | **Adam33** |
| 54598 | 4737 | 0,4119 | 4,4558 | -4,8370 | 9,70E-05 | 0,0033 | **Calcrl** |
| 109648 | 547 | 0,0547 | 0,4208 | -4,8161 | 0,0001 | 0,0034 | **Npy** |
| 20393 | 4158 | 0,2619 | 6,0922 | -4,8123 | 0,0001 | 0,0035 | **Sgk1** |
| 100504211 | 3928 | 0,3337 | 3,7053 | -4,7689 | 0,0001 | 0,0037 | **Gm20114** |
| 23882 | 1081 | 0,3056 | 3,4299 | -4,7425 | 0,0001 | 0,0039 | **Gadd45g** |
| 12053 | 3285 | 0,4708 | 6,2710 | -4,7403 | 0,0001 | 0,0039 | **Bcl6** |
| 74481 | 1372 | 0,4787 | 4,9792 | -4,7280 | 0,0001 | 0,0040 | **Batf2** |
| 14064 | 2464 | 0,2535 | 0,0167 | -4,7023 | 0,0001 | 0,0042 | **F2rl2** |
| 20612 | 6387 | 0,4264 | 8,2304 | -4,6753 | 0,0001 | 0,0044 | **Siglec1** |
| 80752 | 3001 | 0,3157 | 7,2085 | -4,6362 | 0,0002 | 0,0047 | **Fam20c** |
| 66569 | 2484 | 0,3278 | 3,2234 | -4,5961 | 0,0002 | 0,0050 | **Gdpd1** |
| 216799 | 4021 | 0,4943 | 8,8545 | -4,5934 | 0,0002 | 0,0050 | **Nlrp3** |
| 245945 | 5368 | 0,4374 | 6,5350 | -4,5905 | 0,0002 | 0,0050 | **Rbm47** |
| 319182 | 381 | 0,1223 | -0,4743 | -4,5859 | 0,0002 | 0,0051 | **Hist1h2bh** |
| 12984 | 4619 | 0,3005 | 7,4659 | -4,5586 | 0,0002 | 0,0053 | **Csf2rb2** |
| 244237 | 2977 | 0,2759 | 1,6267 | -4,5574 | 0,0002 | 0,0053 | **Tnfrsf26** |
| 54125 | 2925 | 0,4618 | 3,1984 | -4,5440 | 0,0002 | 0,0054 | **Polm** |
| 23845 | 3820 | 0,4078 | 5,4696 | -4,5340 | 0,0002 | 0,0055 | **Clec5a** |
| 83921 | 6667 | 0,4599 | 7,5745 | -4,5279 | 0,0002 | 0,0055 | **Tmem2** |
| 319169 | 393 | 0,1010 | 0,5486 | -4,5172 | 0,0002 | 0,0056 | **Hist1h2ak** |
| 215723 | 2060 | 0,2815 | 1,3387 | -4,5139 | 0,0002 | 0,0056 | **Mfsd6l** |
| 29877 | 5868 | 0,2585 | 3,1447 | -4,4916 | 0,0002 | 0,0058 | **Hdgfrp3** |
| 16181 | 2691 | 0,2713 | 7,2584 | -4,4742 | 0,0002 | 0,0060 | **Il1rn** |
| 100502583 | 447 | 0,1473 | -1,3257 | -4,4480 | 0,0002 | 0,0063 | **Gm19263** |
| 22793 | 2438 | 0,4093 | 7,3472 | -4,4412 | 0,0002 | 0,0064 | **Zyx** |
| 72691 | 2049 | 0,1884 | 2,8758 | -4,4356 | 0,0002 | 0,0064 | **Calhm2** |
| 12402 | 5083 | 0,4288 | 6,4886 | -4,4245 | 0,0003 | 0,0065 | **Cbl** |
| 21815 | 2674 | 0,4632 | 6,5654 | -4,4198 | 0,0003 | 0,0066 | **Tgif1** |
| 107769 | 2202 | 0,4600 | 5,1134 | -4,4073 | 0,0003 | 0,0067 | **Tm6sf1** |
| 227737 | 3693 | 0,4652 | 7,2336 | -4,3944 | 0,0003 | 0,0069 | **Fam129b** |
| 66412 | 3955 | 0,3364 | 6,9058 | -4,3908 | 0,0003 | 0,0069 | **Arrdc4** |
| 18950 | 2941 | 0,4898 | 8,8390 | -4,3877 | 0,0003 | 0,0069 | **Pnp** |
| 227671 | 1335 | 0,2902 | 2,0461 | -4,3832 | 0,0003 | 0,0069 | **Gbgt1** |
| 76441 | 6045 | 0,2672 | 2,0221 | -4,3621 | 0,0003 | 0,0072 | **Daam2** |
| 381310 | 6557 | 0,1815 | 0,3466 | -4,3533 | 0,0003 | 0,0072 | **6330403A02Rik** |
| 15466 | 2793 | 0,2704 | 1,3549 | -4,3502 | 0,0003 | 0,0073 | **Hrh2** |
| 64898 | 5868 | 0,4858 | 7,5429 | -4,3399 | 0,0003 | 0,0074 | **Lpin2** |
| 58198 | 5258 | 0,3999 | 4,0145 | -4,3178 | 0,0003 | 0,0077 | **Sall1** |
| 100504287 | 1468 | 0,4887 | 3,8149 | -4,3134 | 0,0003 | 0,0078 | **Gm16340** |
| 20310 | 1083 | 0,1424 | 6,8974 | -4,3101 | 0,0003 | 0,0078 | **Cxcl2** |
| 11639 | 5162 | 0,3914 | 2,9864 | -4,2908 | 0,0003 | 0,0080 | **Ak4** |
| 93841 | 1162 | 0,4947 | 3,4650 | -4,2882 | 0,0004 | 0,0080 | **Uchl4** |
| 630294 | 899 | 0,4977 | 1,9521 | -4,2882 | 0,0004 | 0,0080 | **Gm7030** |
| 71843 | 1725 | 0,2951 | 1,1909 | -4,2546 | 0,0004 | 0,0086 | **R3hcc1** |
| 15001 | 1049 | 0,1528 | -1,1125 | -4,2244 | 0,0004 | 0,0090 | **H2-Oa** |
| 81913 | 1215 | 0,4037 | 1,5029 | -4,2033 | 0,0004 | 0,0094 | **Bambi-ps1** |
| 100504231 | 1766 | 0,3521 | 2,2232 | -4,1557 | 0,0005 | 0,0101 | **Gm15708** |
| 319487 | 2793 | 0,4846 | 2,2022 | -4,1532 | 0,0005 | 0,0102 | **A230028O05Rik** |
| 100502959 | 627 | 0,4233 | 3,3057 | -4,1516 | 0,0005 | 0,0102 | **AV051173** |
| 27261 | 1546 | 0,3249 | 3,8321 | -4,1459 | 0,0005 | 0,0103 | **Dok3** |
| 100503570 | 1472 | 0,3233 | 2,3066 | -4,1429 | 0,0005 | 0,0104 | **Gm19773** |
| 625421 | 1461 | 0,4383 | 4,2791 | -4,1418 | 0,0005 | 0,0104 | **C230062I16Rik** |
| 14289 | 1296 | 0,4998 | 6,4526 | -4,1428 | 0,0005 | 0,0104 | **Fpr2** |
| 14077 | 640 | 0,4009 | 1,5123 | -4,1395 | 0,0005 | 0,0104 | **Fabp3** |
| 270685 | 4332 | 0,4300 | 4,0186 | -4,1324 | 0,0005 | 0,0105 | **Mthfd1l** |
| 58218 | 994 | 0,1447 | -1,5099 | -4,1285 | 0,0005 | 0,0106 | **Trem3** |
| 263803 | 2931 | 0,1592 | 0,1346 | -4,1276 | 0,0005 | 0,0106 | **Pkn3** |
| 16175 | 1974 | 0,3524 | 8,3743 | -4,1102 | 0,0005 | 0,0109 | **Il1a** |
| 17387 | 2597 | 0,3272 | 7,4977 | -4,1063 | 0,0005 | 0,0110 | **Mmp14** |
| 244864 | 1786 | 0,3409 | 2,2271 | -4,0972 | 0,0005 | 0,0111 | **Layn** |
| 66898 | 3248 | 0,4373 | 3,6303 | -4,0928 | 0,0006 | 0,0112 | **Baiap2l1** |
| 56702 | 672 | 0,1638 | 0,9131 | -4,0819 | 0,0006 | 0,0113 | **Hist1h1b** |
| 20238 | 10608 | 0,4921 | 5,3946 | -4,0799 | 0,0006 | 0,0114 | **Atxn1** |
| 633188 | 546 | 0,2497 | -1,9996 | -4,0576 | 0,0006 | 0,0117 | **LOC633188** |
| 435653 | 1284 | 0,1161 | -0,3895 | -4,0541 | 0,0006 | 0,0118 | **Fcrlb** |
| 209645 | 3837 | 0,2332 | 0,6464 | -4,0504 | 0,0006 | 0,0119 | **Bend7** |
| 111241 | 1599 | 0,4681 | 4,7970 | -4,0501 | 0,0006 | 0,0119 | **Hmga1-rs1** |
| 277089 | 675 | 0,1647 | 0,4566 | -4,0315 | 0,0006 | 0,0123 | **Gm5068** |
| 16918 | 3501 | 0,2090 | 1,9216 | -4,0328 | 0,0006 | 0,0123 | **Mycl1** |
| 56699 | 3375 | 0,4496 | 4,9024 | -4,0267 | 0,0006 | 0,0123 | **Cdc42ep4** |
| 414801 | 3766 | 0,3436 | 4,3708 | -4,0228 | 0,0007 | 0,0124 | **Itprip** |
| 71409 | 5851 | 0,3897 | 7,7710 | -4,0011 | 0,0007 | 0,0129 | **Fmnl2** |
| 12703 | 1220 | 0,4457 | 5,0554 | -3,9999 | 0,0007 | 0,0129 | **Socs1** |
| 18175 | 5499 | 0,2427 | 2,5412 | -3,9905 | 0,0007 | 0,0131 | **Nrap** |
| 27226 | 1915 | 0,2475 | 5,7935 | -3,9876 | 0,0007 | 0,0131 | **Pla2g7** |
| 74748 | 2040 | 0,3035 | 5,3351 | -3,9866 | 0,0007 | 0,0132 | **Slamf8** |
| 14936 | 3681 | 0,3559 | 3,5738 | -3,9789 | 0,0007 | 0,0133 | **Gys1** |
| 216850 | 6654 | 0,4148 | 5,4110 | -3,9763 | 0,0007 | 0,0134 | **Kdm6b** |
| 233424 | 4316 | 0,2046 | 0,3515 | -3,9668 | 0,0007 | 0,0136 | **Tmc3** |
| 14254 | 6280 | 0,4795 | 4,3502 | -3,9433 | 0,0008 | 0,0142 | **Flt1** |
| 21941 | 1575 | 0,3118 | 1,5187 | -3,9242 | 0,0008 | 0,0148 | **Tnfrsf8** |
| 242093 | 1245 | 0,2515 | -0,2870 | -3,8981 | 0,0009 | 0,0154 | **Rxfp4** |
| 13197 | 1224 | 0,3201 | 2,2289 | -3,8924 | 0,0009 | 0,0155 | **Gadd45a** |
| 212980 | 3426 | 0,2518 | 1,1985 | -3,8860 | 0,0009 | 0,0156 | **Slc45a3** |
| 74123 | 4025 | 0,4376 | 4,6315 | -3,8611 | 0,0010 | 0,0163 | **Foxp4** |
| 17295 | 6652 | 0,2235 | 1,3518 | -3,8584 | 0,0010 | 0,0164 | **Met** |
| 170829 | 1409 | 0,2646 | 1,6244 | -3,8511 | 0,0010 | 0,0166 | **Tram2** |
| 67102 | 6160 | 0,4687 | 4,1575 | -3,8458 | 0,0010 | 0,0167 | **D16Ertd472e** |
| 68190 | 1815 | 0,4812 | 3,8712 | -3,8255 | 0,0010 | 0,0172 | **5330426P16Rik** |
| 78749 | 4044 | 0,4287 | 6,1649 | -3,8192 | 0,0011 | 0,0173 | **Filip1l** |
| 232801 | 2109 | 0,2697 | 1,8611 | -3,8056 | 0,0011 | 0,0177 | **Lilra5** |
| 100038618 | 1669 | 0,4352 | 3,6497 | -3,8032 | 0,0011 | 0,0177 | **Gm10847** |
| 100861682 | 632 | 0,2941 | -1,5925 | -3,7708 | 0,0012 | 0,0188 |  |
| 20555 | 1857 | 0,4566 | 3,8368 | -3,7613 | 0,0012 | 0,0191 | **Slfn1** |
| 11898 | 1631 | 0,2560 | 0,4201 | -3,7404 | 0,0013 | 0,0199 | **Ass1** |
| 57742 | 1356 | 0,1878 | -1,5813 | -3,7384 | 0,0013 | 0,0199 | **Abhd1** |
| 52398 | 5008 | 0,3320 | 6,4928 | -3,7303 | 0,0013 | 0,0201 | **sep-11** |
| 69716 | 2267 | 0,3129 | 1,1103 | -3,7260 | 0,0013 | 0,0203 | **Trip13** |
| 215493 | 3390 | 0,2437 | -0,0625 | -3,7222 | 0,0013 | 0,0203 | **A3galt2** |
| 108075 | 5595 | 0,1420 | -0,2220 | -3,7057 | 0,0014 | 0,0209 | **Ltbp4** |
| 319924 | 6632 | 0,4221 | 4,0714 | -3,6946 | 0,0014 | 0,0213 | **Apba1** |
| 100861531 | 5868 | 0,3164 | 14,4397 | -3,6830 | 0,0015 | 0,0217 |  |
| 319155 | 312 | 0,3276 | 3,9176 | -3,6826 | 0,0015 | 0,0217 | **Hist1h4c** |
| 100141474 | 1425 | 0,1996 | -0,6292 | -3,6692 | 0,0015 | 0,0221 | **4933428G20Rik** |
| 100503460 | 489 | 0,1733 | -1,7674 | -3,6681 | 0,0015 | 0,0222 | **Gm19705** |
| 21817 | 3549 | 0,2640 | 6,9447 | -3,6593 | 0,0015 | 0,0225 | **Tgm2** |
| 56615 | 943 | 0,3724 | 4,1810 | -3,6589 | 0,0015 | 0,0225 | **Mgst1** |
| 74482 | 1823 | 0,3927 | 1,2477 | -3,6531 | 0,0016 | 0,0227 | **Ifitm7** |
| 11853 | 1056 | 0,4993 | 6,5081 | -3,6506 | 0,0016 | 0,0227 | **Rhoc** |
| 100628626 | 82 | 0,0284 | 9,6043 | -3,6386 | 0,0016 | 0,0231 | **Mir5105** |
| 665563 | 2243 | 0,3481 | 1,2850 | -3,6124 | 0,0017 | 0,0241 | **Mthfd2l** |
| 74516 | 703 | 0,2941 | -2,0698 | -3,6046 | 0,0017 | 0,0245 | **A730049H05Rik** |
| 723827 | 95 | 0,3972 | 2,0559 | -3,6030 | 0,0017 | 0,0245 | **Mir221** |
| 14745 | 3522 | 0,1287 | 1,6317 | -3,5989 | 0,0018 | 0,0247 | **Lpar1** |
| 218820 | 4213 | 0,1993 | 1,1496 | -3,5876 | 0,0018 | 0,0250 | **Zfp503** |
| 216285 | 2493 | 0,1577 | -1,4483 | -3,5819 | 0,0018 | 0,0252 | **Alx1** |
| 14247 | 3087 | 0,4680 | 4,1534 | -3,5813 | 0,0018 | 0,0252 | **Fli1** |
| 12609 | 2260 | 0,3969 | 5,2971 | -3,5655 | 0,0019 | 0,0258 | **Cebpd** |
| 19791 | 938 | 0,0635 | 7,5468 | -3,5498 | 0,0020 | 0,0266 | **Rn18s** |
| 100039062 | 3025 | 0,2157 | -0,7657 | -3,5361 | 0,0020 | 0,0272 | **Gm2027** |
| 18793 | 1396 | 0,4901 | 6,5043 | -3,5342 | 0,0021 | 0,0273 | **Plaur** |
| 73737 | 657 | 0,3428 | 2,1921 | -3,5205 | 0,0021 | 0,0279 | **1110008P14Rik** |
| 17119 | 4662 | 0,4877 | 6,2456 | -3,4953 | 0,0022 | 0,0289 | **Mxd1** |
| 14087 | 4541 | 0,3139 | 1,6785 | -3,4815 | 0,0023 | 0,0294 | **Fanca** |
| 23886 | 1084 | 0,4951 | 3,0671 | -3,4759 | 0,0024 | 0,0297 | **Gdf15** |
| 100503677 | 1644 | 0,1734 | 0,3655 | -3,4703 | 0,0024 | 0,0299 | **Gm19826** |
| 212919 | 4266 | 0,3640 | 1,3069 | -3,4637 | 0,0024 | 0,0302 | **Kctd7** |
| 330188 | 2283 | 0,3651 | 1,4176 | -3,4538 | 0,0025 | 0,0307 | **Ccdc63** |
| 11479 | 3277 | 0,4835 | 4,0083 | -3,4536 | 0,0025 | 0,0307 | **Acvr1b** |
| 244091 | 2976 | 0,1896 | -0,9150 | -3,4473 | 0,0025 | 0,0311 | **Fsd2** |
| 320456 | 2152 | 0,2708 | 0,9753 | -3,4476 | 0,0025 | 0,0311 | **B330016D10Rik** |
| 108078 | 3569 | 0,2567 | 1,3212 | -3,4451 | 0,0025 | 0,0312 | **Olr1** |
| 108811 | 1721 | 0,3534 | 3,4409 | -3,4394 | 0,0026 | 0,0315 | **Ccdc122** |
| 12953 | 4368 | 0,4305 | 2,6500 | -3,4389 | 0,0026 | 0,0315 | **Cry2** |
| 27416 | 6197 | 0,4717 | 6,6481 | -3,4340 | 0,0026 | 0,0316 | **Abcc5** |
| 16159 | 1370 | 0,1681 | -1,2544 | -3,4098 | 0,0027 | 0,0328 | **Il12a** |
| 666642 | 647 | 0,2497 | -1,6799 | -3,3637 | 0,0031 | 0,0353 | **Gm8210** |
| 100861939 | 591 | 0,1767 | 0,6436 | -3,3629 | 0,0031 | 0,0353 |  |
| 387221 | 73 | 0,2263 | -0,5828 | -3,3612 | 0,0031 | 0,0354 | **Mir27b** |
| 71839 | 1961 | 0,2697 | 1,8074 | -3,3432 | 0,0032 | 0,0364 | **Osgin1** |
| 19217 | 3768 | 0,1918 | -0,0418 | -3,3385 | 0,0032 | 0,0366 | **Ptger2** |
| 24064 | 2087 | 0,3403 | 2,1961 | -3,3279 | 0,0033 | 0,0374 | **Spry2** |
| 360198 | 534 | 0,3445 | 1,7540 | -3,3272 | 0,0033 | 0,0374 | **Hist1h3a** |
| 232941 | 1631 | 0,3176 | 0,0656 | -3,2850 | 0,0037 | 0,0399 | **Ppm1n** |
| 16169 | 1548 | 0,4531 | 5,6921 | -3,2791 | 0,0037 | 0,0402 | **Il15ra** |
| 19221 | 5786 | 0,2535 | 2,5296 | -3,2732 | 0,0038 | 0,0405 | **Ptgfrn** |
| 54427 | 1885 | 0,1956 | -1,3984 | -3,2639 | 0,0038 | 0,0411 | **Dnmt3l** |
| 327762 | 4122 | 0,2496 | 0,9819 | -3,2484 | 0,0040 | 0,0422 | **Dna2** |
| 18158 | 780 | 0,2644 | -0,4437 | -3,2472 | 0,0040 | 0,0423 | **Nppb** |
| 207819 | 4594 | 0,1840 | -0,6968 | -3,2192 | 0,0043 | 0,0441 | **4930539E08Rik** |
| 320484 | 3677 | 0,4383 | 3,6099 | -3,2101 | 0,0043 | 0,0446 | **Rasal3** |
| 381290 | 8292 | 0,3803 | 2,7746 | -3,1931 | 0,0045 | 0,0458 | **Atp2b4** |
| 100380944 | 1802 | 0,3200 | 1,1344 | -3,1915 | 0,0045 | 0,0459 | **Gm11602** |
| 14283 | 1705 | 0,1981 | -0,5112 | -3,1884 | 0,0046 | 0,0461 | **Fosl1** |
| 195522 | 2396 | 0,4415 | 2,0361 | -3,1871 | 0,0046 | 0,0462 | **Zfp691** |
| 635504 | 465 | 0,4177 | 1,4839 | -3,1842 | 0,0046 | 0,0464 | **Gm7160** |
| 72104 | 657 | 0,2756 | -1,8189 | -3,1778 | 0,0047 | 0,0469 | **2010106C02Rik** |
| 328830 | 1900 | 0,3067 | 0,7913 | -3,1758 | 0,0047 | 0,0471 | **A530064D06Rik** |
| 69386 | 479 | 0,2724 | 2,8488 | -3,1744 | 0,0047 | 0,0472 | **Hist1h4h** |
| 319179 | 2510 | 0,4167 | 3,4684 | -3,1665 | 0,0048 | 0,0477 | **Hist1h2be** |
| 100043205 | 461 | 0,1942 | -1,7190 | -3,1616 | 0,0049 | 0,0480 | **Gm13815** |
| 381853 | 1792 | 0,3293 | 0,0673 | -3,1525 | 0,0050 | 0,0486 | **Gipr** |
| 319186 | 381 | 0,2349 | 0,5077 | -3,1471 | 0,0050 | 0,0490 | **Hist1h2bm** |
