# Supplementary material for: Myeloid C/EBPβ deficiency reshapes microglial gene expression and is protective in experimental autoimmune encephalomyelitis
Source: J Neuroinflammation. 2017 Mar 16;14:54. doi: 10.1186/s12974-017-0834-5 (PMC5356255; doi:10.1186/s12974-017-0834-5)
Supplement: Additional file 1: Tables S1-S6. — List the genes significantly up-regulated (tables 1, 3 and 5) or down-regulated (tables 2, 4 and 6) by the absence of C/EBPβ in control (tables 1, 2), LPS-treated (tables 3, 4) and LPS+IFNγ-treated (tables 5, 6) primary microglial cultures. These data were obtained by RNAseq as described in Methods. (ZIP 253 kb) [file 12974_2017_834_MOESM1_ESM.zip › 12974_2017_834_MOESM1_ESM/Table S5.docx]

| **Table S5** |
| --- |
| **Genes with significantly increased expression in LysMCre-CEBPbetafl/fl microglia in LPS+IFNg condition** |

| **GeneID** | **Length** | **FC** | **AveExpr** | **t** | **P.Value** | **adj.P** | **gene_symbol** |
| --- | --- | --- | --- | --- | --- | --- | --- |
| 76088 | 7810 | 3,6161 | 7,6778 | 11,6657 | 1,93E-10 | 5,88E-07 | **Dock8** |
| 20947 | 4035 | 4,4076 | 9,3066 | 11,4762 | 2,58E-10 | 6,55E-07 | **Swap70** |
| 99138 | 3118 | 4,3552 | 6,7911 | 8,8630 | 2,09E-08 | 2,45E-05 | **Stard7** |
| 74048 | 4846 | 2,4865 | 9,0228 | 8,6434 | 3,14E-08 | 2,88E-05 | **4632428N05Rik** |
| 71653 | 4081 | 3,5792 | 5,7887 | 8,4286 | 4,70E-08 | 3,41E-05 | **4930506M07Rik** |
| 74006 | 4058 | 2,0461 | 7,0801 | 7,8921 | 1,32E-07 | 6,95E-05 | **Dnm1l** |
| 67972 | 7130 | 2,0551 | 7,7482 | 7,4564 | 3,15E-07 | 0,0001 | **Atp2b1** |
| 26382 | 2625 | 4,7180 | 6,8087 | 7,4035 | 3,51E-07 | 0,0001 | **Fgd2** |
| 66058 | 1119 | 2,3060 | 7,5960 | 7,2764 | 4,54E-07 | 0,0001 | **Tmem176a** |
| 238328 | 6189 | 2,7255 | 5,6338 | 7,2339 | 4,96E-07 | 0,0001 | **Vash1** |
| 12517 | 1503 | 4,1442 | 7,2564 | 7,2280 | 5,02E-07 | 0,0001 | **Cd72** |
| 246177 | 3307 | 3,0594 | 7,6591 | 7,2077 | 5,23E-07 | 0,0001 | **Myo1g** |
| 24056 | 2482 | 2,7679 | 7,1963 | 7,2077 | 5,23E-07 | 0,0001 | **Sh3bp5** |
| 56758 | 5809 | 2,7579 | 9,0985 | 7,0780 | 6,84E-07 | 0,0002 | **Mbnl1** |
| 12183 | 2068 | 5,8391 | 3,5006 | 7,0484 | 7,27E-07 | 0,0002 | **Bpgm** |
| 232288 | 5029 | 2,9213 | 7,4085 | 7,0279 | 7,59E-07 | 0,0002 | **Frmd4b** |
| 74182 | 5052 | 3,3072 | 5,8985 | 7,0019 | 8,01E-07 | 0,0002 | **Gpcpd1** |
| 13448 | 1807 | 2,2391 | 5,7767 | 6,9949 | 8,13E-07 | 0,0002 | **Dok1** |
| 17118 | 4186 | 2,0127 | 10,0894 | 6,8968 | 9,98E-07 | 0,0002 | **Marcks** |
| 50723 | 2748 | 3,2495 | 6,9954 | 6,6418 | 1,71E-06 | 0,0003 | **Icosl** |
| 16491 | 1968 | 2,8818 | 4,5127 | 6,6262 | 1,77E-06 | 0,0003 | **Kcna3** |
| 72129 | 4122 | 2,2299 | 5,0991 | 6,5931 | 1,90E-06 | 0,0004 | **Pex13** |
| 211329 | 5338 | 2,0281 | 6,7157 | 6,5503 | 2,08E-06 | 0,0004 | **Ncoa7** |
| 69780 | 2905 | 2,4971 | 7,4942 | 6,5197 | 2,22E-06 | 0,0004 | **Smap2** |
| 14824 | 2333 | 2,2569 | 10,4006 | 6,4728 | 2,46E-06 | 0,0004 | **Grn** |
| 54712 | 7045 | 3,4774 | 7,3783 | 6,4552 | 2,55E-06 | 0,0004 | **Plxnc1** |
| 67092 | 2357 | 4,0494 | 6,3207 | 6,4410 | 2,63E-06 | 0,0004 | **Gatm** |
| 64380 | 1723 | 2,8685 | 6,6296 | 6,4184 | 2,76E-06 | 0,0004 | **Ms4a4c** |
| 100040852 | 5217 | 8,1909 | 0,1807 | 6,3988 | 2,88E-06 | 0,0005 | **Gm3002** |
| 208154 | 3235 | 6,7231 | -0,7194 | 6,3353 | 3,31E-06 | 0,0005 | **Btla** |
| 16561 | 14849 | 2,4871 | 8,4352 | 6,2855 | 3,68E-06 | 0,0005 | **Kif1b** |
| 19655 | 3743 | 3,4126 | 4,6235 | 6,2853 | 3,69E-06 | 0,0005 | **Rbmx** |
| 26879 | 1901 | 2,2865 | 6,1695 | 6,2244 | 4,21E-06 | 0,0006 | **B3galnt1** |
| 67710 | 868 | 2,1196 | 6,0476 | 6,2068 | 4,37E-06 | 0,0006 | **Polr2g** |
| 66540 | 3148 | 2,8545 | 7,5408 | 6,2005 | 4,43E-06 | 0,0006 | **Fam107b** |
| 83704 | 3315 | 2,1486 | 6,9015 | 6,1090 | 5,42E-06 | 0,0007 | **Slc12a9** |
| 70314 | 2539 | 2,1288 | 6,0261 | 6,0126 | 6,70E-06 | 0,0008 | **Rabep2** |
| 30941 | 2185 | 2,0548 | 5,4838 | 5,9984 | 6,92E-06 | 0,0009 | **Usp21** |
| 67283 | 2792 | 3,4219 | 3,8830 | 5,9468 | 7,75E-06 | 0,0009 | **Slc25a19** |
| 12578 | 1136 | 3,5312 | 3,6126 | 5,9464 | 7,76E-06 | 0,0009 | **Cdkn2a** |
| 15488 | 2685 | 2,0774 | 6,1614 | 5,8950 | 8,70E-06 | 0,0010 | **Hsd17b4** |
| 224132 | 4554 | 2,1650 | 5,2912 | 5,8925 | 8,75E-06 | 0,0010 | **Dirc2** |
| 432479 | 695 | 2,1418 | 5,0755 | 5,8159 | 1,04E-05 | 0,0011 | **4930404N11Rik** |
| 11307 | 5821 | 2,7701 | 7,9340 | 5,8118 | 1,05E-05 | 0,0011 | **Abcg1** |
| 98365 | 1145 | 5,6697 | 6,6756 | 5,7527 | 1,20E-05 | 0,0013 | **Slamf9** |
| 230674 | 4956 | 2,1643 | 7,5160 | 5,7061 | 1,33E-05 | 0,0014 | **Kdm4a** |
| 72699 | 1959 | 2,0658 | 4,6916 | 5,6702 | 1,44E-05 | 0,0015 | **Lime1** |
| 11975 | 4029 | 2,3767 | 7,6104 | 5,6355 | 1,56E-05 | 0,0015 | **Atp6v0a1** |
| 226594 | 2670 | 2,4689 | 7,0621 | 5,6290 | 1,58E-05 | 0,0015 | **Rcsd1** |
| 67041 | 3482 | 2,1873 | 6,4339 | 5,6237 | 1,60E-05 | 0,0015 | **Oxct1** |
| 269999 | 1996 | 2,3793 | 5,0593 | 5,5998 | 1,69E-05 | 0,0016 | **Orai3** |
| 225215 | 1522 | 2,0184 | 4,4828 | 5,5433 | 1,92E-05 | 0,0018 | **Rsl24d1** |
| 69263 | 1215 | 2,4832 | 3,8907 | 5,5319 | 1,97E-05 | 0,0018 | **Rfc3** |
| 67016 | 5936 | 2,4113 | 7,4201 | 5,4142 | 2,57E-05 | 0,0022 | **Tbc1d2b** |
| 67164 | 1229 | 4,0926 | 2,4114 | 5,3938 | 2,69E-05 | 0,0023 | **Lipt2** |
| 80907 | 2031 | 3,3948 | 3,7718 | 5,3784 | 2,79E-05 | 0,0023 | **Lactb** |
| 20439 | 2488 | 2,2557 | 5,5680 | 5,3248 | 3,15E-05 | 0,0025 | **Siah2** |
| 217835 | 4007 | 2,6560 | 6,3547 | 5,3171 | 3,20E-05 | 0,0025 | **Rin3** |
| 210992 | 1605 | 2,0010 | 6,5939 | 5,3098 | 3,26E-05 | 0,0025 | **Lpcat1** |
| 15931 | 4973 | 2,0430 | 5,7033 | 5,2711 | 3,56E-05 | 0,0027 | **Ids** |
| 210035 | 6515 | 2,1897 | 4,1630 | 5,1877 | 4,31E-05 | 0,0030 | **Tmem194** |
| 101148 | 4141 | 2,7178 | 6,5854 | 5,1853 | 4,34E-05 | 0,0030 | **B630005N14Rik** |
| 23912 | 2143 | 3,9554 | 2,7176 | 5,1842 | 4,35E-05 | 0,0030 | **Rhof** |
| 77938 | 5199 | 3,3415 | 4,9093 | 5,1484 | 4,72E-05 | 0,0032 | **Fam53b** |
| 214459 | 5330 | 2,5020 | 7,6323 | 5,1451 | 4,76E-05 | 0,0032 | **Fnbp1l** |
| 226551 | 6308 | 3,1921 | 8,0283 | 5,1259 | 4,97E-05 | 0,0033 | **AI848100** |
| 110958 | 2051 | 11,5417 | 1,8170 | 5,1127 | 5,12E-05 | 0,0033 | **D6Mm5e** |
| 73341 | 4569 | 5,3066 | 5,7759 | 5,1008 | 5,27E-05 | 0,0034 | **Arhgef6** |
| 56306 | 2640 | 3,1631 | 2,4384 | 5,0845 | 5,47E-05 | 0,0035 | **Fam60a** |
| 100637 | 1906 | 2,2132 | 4,5383 | 5,0642 | 5,73E-05 | 0,0037 | **N4bp2l1** |
| 229473 | 4909 | 2,8018 | 5,6857 | 5,0540 | 5,87E-05 | 0,0037 | **D930015E06Rik** |
| 229694 | 5544 | 2,7175 | 5,2407 | 5,0492 | 5,93E-05 | 0,0037 | **AI504432** |
| 18479 | 3061 | 2,1765 | 5,0213 | 5,0372 | 6,10E-05 | 0,0038 | **Pak1** |
| 244152 | 2800 | 2,9997 | 4,7715 | 5,0296 | 6,21E-05 | 0,0039 | **Tsku** |
| 320333 | 1413 | 7,0629 | -1,2442 | 5,0254 | 6,27E-05 | 0,0039 | **D830030K20Rik** |
| 227327 | 2595 | 2,0802 | 3,2537 | 5,0205 | 6,34E-05 | 0,0039 | **B3gnt7** |
| 68241 | 816 | 5,7551 | 0,2926 | 5,0141 | 6,43E-05 | 0,0040 | **Fam195a** |
| 211798 | 3152 | 2,3180 | 4,0787 | 4,9771 | 7,01E-05 | 0,0042 | **Mfsd9** |
| 110006 | 2456 | 2,6964 | 7,5965 | 4,9550 | 7,38E-05 | 0,0044 | **Gusb** |
| 234733 | 6750 | 2,0659 | 5,3449 | 4,9389 | 7,66E-05 | 0,0045 | **Ddx19b** |
| 215690 | 12767 | 3,4780 | 5,2083 | 4,8905 | 8,57E-05 | 0,0049 | **Nav1** |
| 93842 | 4249 | 5,8071 | 4,8677 | 4,8798 | 8,78E-05 | 0,0050 | **Igsf9** |
| 73149 | 1330 | 3,3490 | 6,1703 | 4,8796 | 8,79E-05 | 0,0050 | **Clec4a3** |
| 218973 | 4174 | 2,6333 | 3,3387 | 4,8748 | 8,88E-05 | 0,0050 | **Wdhd1** |
| 269604 | 4762 | 4,7975 | 3,8098 | 4,8554 | 9,29E-05 | 0,0052 | **Gpr157** |
| 11801 | 2037 | 4,5575 | 3,9169 | 4,8440 | 9,54E-05 | 0,0053 | **Cd5l** |
| 16439 | 8331 | 2,2231 | 7,8538 | 4,8181 | 0,00010 | 0,0055 | **Itpr2** |
| 50498 | 1171 | 2,8590 | 6,3309 | 4,8140 | 0,00010 | 0,0055 | **Ebi3** |
| 14190 | 3769 | 3,8240 | 10,2366 | 4,8082 | 0,00010 | 0,0056 | **Fgl2** |
| 229003 | 10109 | 2,0350 | 10,8837 | 4,7982 | 0,00011 | 0,0056 | **BC006779** |
| 329650 | 10249 | 2,6156 | 3,0578 | 4,7981 | 0,00011 | 0,0056 | **Med12l** |
| 72750 | 5532 | 2,4739 | 5,2640 | 4,7866 | 0,00011 | 0,0058 | **Fam117b** |
| 224143 | 2641 | 2,9400 | 5,5251 | 4,7672 | 0,00011 | 0,0060 | **Poglut1** |
| 231821 | 2399 | 2,1076 | 5,9561 | 4,7565 | 0,00012 | 0,0061 | **Adap1** |
| 12579 | 1393 | 2,8907 | 3,3634 | 4,7375 | 0,00012 | 0,0063 | **Cdkn2b** |
| 224109 | 3559 | 2,0980 | 7,5400 | 4,7312 | 0,00012 | 0,0064 | **Lrrc33** |
| 100504333 | 1960 | 2,7427 | 4,2975 | 4,7301 | 0,00012 | 0,0064 | **Gm16712** |
| 240913 | 3673 | 2,5076 | 4,3726 | 4,7251 | 0,00013 | 0,0064 | **Adamts4** |
| 239102 | 8725 | 7,1344 | 2,6504 | 4,7204 | 0,00013 | 0,0064 | **Zfhx2** |
| 544817 | 5627 | 2,4237 | 5,7917 | 4,7057 | 0,00013 | 0,0066 | **Arhgap27** |
| 232670 | 1959 | 2,5730 | 5,1125 | 4,6968 | 0,00013 | 0,0067 | **Tspan33** |
| 19252 | 1942 | 2,9136 | 6,3331 | 4,6840 | 0,00014 | 0,0068 | **Dusp1** |
| 20338 | 6300 | 2,2856 | 8,7109 | 4,6794 | 0,00014 | 0,0068 | **Sel1l** |
| 104759 | 1978 | 2,8652 | 6,7981 | 4,6427 | 0,00015 | 0,0073 | **Pld4** |
| 78334 | 5807 | 2,2796 | 4,9378 | 4,6362 | 0,00015 | 0,0074 | **Cdk19** |
| 72057 | 1665 | 2,5792 | 5,6336 | 4,5981 | 0,00017 | 0,0078 | **Phf10** |
| 67475 | 4239 | 2,0715 | 4,5726 | 4,5620 | 0,00018 | 0,0084 | **Ero1lb** |
| 192652 | 6909 | 2,1913 | 7,1577 | 4,5616 | 0,00018 | 0,0084 | **Wdr81** |
| 227326 | 2550 | 6,5372 | -0,7216 | 4,5329 | 0,00020 | 0,0088 | **Gpr55** |
| 19893 | 6751 | 3,0840 | 2,4606 | 4,5270 | 0,00020 | 0,0089 | **Rpgr** |
| 215095 | 2377 | 8,2882 | -0,1515 | 4,5177 | 0,00020 | 0,0090 | **Astl** |
| 11629 | 875 | 2,2170 | 7,8743 | 4,4789 | 0,00022 | 0,0098 | **Aif1** |
| 72042 | 1639 | 2,1475 | 7,7661 | 4,4395 | 0,00025 | 0,0106 | **Cotl1** |
| 77048 | 2986 | 2,2335 | 5,7662 | 4,4264 | 0,00025 | 0,0107 | **Ccdc41** |
| 26888 | 2616 | 2,4790 | 6,7746 | 4,3856 | 0,00028 | 0,0115 | **Clec4a2** |
| 11766 | 2673 | 2,4177 | 3,8469 | 4,3716 | 0,00029 | 0,0117 | **Ap1g2** |
| 109163 | 1757 | 4,9499 | 0,2708 | 4,3618 | 0,00029 | 0,0119 | **3010003L21Rik** |
| 16574 | 6840 | 2,3332 | 4,3843 | 4,3340 | 0,00031 | 0,0124 | **Kif5c** |
| 83924 | 2981 | 2,3280 | 5,6250 | 4,3298 | 0,00032 | 0,0125 | **Gpr137b** |
| 71389 | 10549 | 2,0919 | 6,6093 | 4,2188 | 0,00041 | 0,0154 | **Chd6** |
| 239217 | 6057 | 2,4461 | 10,0139 | 4,2099 | 0,00042 | 0,0157 | **Kctd12** |
| 210710 | 2129 | 4,9161 | 2,9236 | 4,2054 | 0,00043 | 0,0158 | **Gab3** |
| 194590 | 7630 | 2,2764 | 4,7501 | 4,1971 | 0,00043 | 0,0160 | **Reps2** |
| 278097 | 2034 | 2,0262 | 3,5174 | 4,1939 | 0,00044 | 0,0160 | **Armcx6** |
| 68738 | 3594 | 2,1965 | 4,2068 | 4,1919 | 0,00044 | 0,0160 | **Acss1** |
| 383435 | 3624 | 2,6209 | 6,8145 | 4,1757 | 0,00046 | 0,0164 | **Ms4a14** |
| 71640 | 3497 | 2,3236 | 2,7764 | 4,1671 | 0,00047 | 0,0166 | **Zfp949** |
| 329154 | 6140 | 2,0532 | 6,1739 | 4,1648 | 0,00047 | 0,0166 | **Ankrd44** |
| 16988 | 416 | 2,3612 | 6,6324 | 4,1413 | 0,00049 | 0,0173 | **Lst1** |
| 17691 | 4509 | 3,9118 | 5,5620 | 4,1274 | 0,00051 | 0,0176 | **Sik1** |
| 67888 | 1764 | 3,4075 | 1,7716 | 4,1244 | 0,00051 | 0,0177 | **Tmem100** |
| 66489 | 452 | 4,8335 | 0,6911 | 4,1077 | 0,00054 | 0,0181 | **Rpl35** |
| 217944 | 3987 | 3,4084 | 7,8187 | 4,1074 | 0,00054 | 0,0181 | **Rapgef5** |
| 100503654 | 3662 | 2,4498 | 4,9148 | 4,0961 | 0,00055 | 0,0184 | **Gm19816** |
| 17869 | 2399 | 3,7685 | 4,2696 | 4,0922 | 0,00056 | 0,0184 | **Myc** |
| 217721 | 3435 | 11,9557 | 3,1364 | 4,0884 | 0,00056 | 0,0185 | **Mfsd7c** |
| 14154 | 6816 | 2,0747 | 5,2409 | 4,0767 | 0,00058 | 0,0188 | **Fem1a** |
| 226525 | 10046 | 2,6478 | 5,3767 | 4,0699 | 0,00059 | 0,0189 | **Rasal2** |
| 232413 | 2222 | 2,5188 | 6,9968 | 4,0605 | 0,00060 | 0,0193 | **Clec12a** |
| 104001 | 3710 | 3,0696 | 4,6930 | 4,0582 | 0,00060 | 0,0194 | **Rtn1** |
| 14073 | 3816 | 3,1203 | 0,5362 | 4,0441 | 0,00062 | 0,0198 | **Faah** |
| 16506 | 1956 | 5,6689 | 0,1669 | 4,0298 | 0,00064 | 0,0203 | **Kcnd1** |
| 100503433 | 891 | 5,7264 | 0,2428 | 4,0270 | 0,00065 | 0,0204 | **Gm15433** |
| 70839 | 2338 | 2,0934 | 4,6034 | 4,0178 | 0,00066 | 0,0207 | **P2ry12** |
| 100038691 | 1413 | 4,1483 | -1,7767 | 4,0069 | 0,00068 | 0,0211 | **Gm10344** |
| 226265 | 2567 | 3,2515 | 1,6813 | 4,0008 | 0,00069 | 0,0213 | **Eno4** |
| 56526 | 6698 | 4,5449 | 2,8318 | 3,9999 | 0,00069 | 0,0213 | **sep-06** |
| 50778 | 1330 | 2,6938 | 7,9750 | 3,9906 | 0,00071 | 0,0215 | **Rgs1** |
| 29873 | 3871 | 9,2852 | 1,8051 | 3,9755 | 0,00073 | 0,0221 | **Cspg5** |
| 69723 | 1213 | 2,3918 | 3,2233 | 3,9677 | 0,00074 | 0,0224 | **Rpain** |
| 100041286 | 592 | 2,2834 | 3,2055 | 3,9447 | 0,00079 | 0,0234 | **Gm11974** |
| 381485 | 1787 | 5,0639 | -1,2663 | 3,9360 | 0,00080 | 0,0238 | **Trim55** |
| 232406 | 2742 | 2,6160 | 2,9877 | 3,9313 | 0,00081 | 0,0239 | **BC035044** |
| 329910 | 5668 | 5,5687 | 1,1786 | 3,9285 | 0,00082 | 0,0239 | **Acot11** |
| 319239 | 3791 | 6,4446 | -0,1562 | 3,9172 | 0,00084 | 0,0243 | **Npsr1** |
| 100861644 | 653 | 3,9645 | -1,8660 | 3,9170 | 0,00084 | 0,0243 |  |
| 544696 | 7240 | 5,0046 | 3,6982 | 3,9024 | 0,00087 | 0,0250 | **D630037F22Rik** |
| 64704 | 1725 | 2,1082 | 4,5167 | 3,8810 | 0,00091 | 0,0259 | **Htra2** |
| 229055 | 7449 | 2,5357 | 3,7863 | 3,8745 | 0,00093 | 0,0262 | **Zbtb10** |
| 71619 | 1200 | 6,0398 | -0,4080 | 3,8689 | 0,00094 | 0,0263 | **Arl14** |
| 63913 | 3939 | 2,1019 | 7,6119 | 3,8469 | 0,00099 | 0,0271 | **Fam129a** |
| 67784 | 6907 | 2,2003 | 5,8692 | 3,8452 | 0,00099 | 0,0271 | **Plxnd1** |
| 20850 | 3888 | 2,0261 | 6,1213 | 3,8357 | 0,00101 | 0,0276 | **Stat5a** |
| 100628592 | 81 | 4,0138 | -1,7501 | 3,8304 | 0,00103 | 0,0278 | **Mir3473d** |
| 12359 | 2551 | 2,2711 | 6,7917 | 3,8013 | 0,00110 | 0,0291 | **Cat** |
| 74191 | 2528 | 2,8044 | 5,9390 | 3,7932 | 0,00112 | 0,0295 | **P2ry13** |
| 98952 | 4217 | 2,8869 | 3,3211 | 3,7689 | 0,00119 | 0,0306 | **Fam102a** |
| 100503615 | 989 | 7,1130 | -0,5733 | 3,7458 | 0,00125 | 0,0318 | **LOC100503615** |
| 13537 | 1618 | 2,2575 | 6,3562 | 3,7277 | 0,00131 | 0,0327 | **Dusp2** |
| 20817 | 6436 | 2,1868 | 5,1502 | 3,7236 | 0,00132 | 0,0329 | **Srpk2** |
| 12862 | 659 | 5,4982 | -0,4001 | 3,7162 | 0,00134 | 0,0334 | **Cox6a2** |
| 53314 | 904 | 2,1358 | 5,3775 | 3,7080 | 0,00137 | 0,0338 | **Batf** |
| 434410 | 690 | 5,0357 | -1,3953 | 3,6970 | 0,00140 | 0,0343 | **Gm17749** |
| 77577 | 1819 | 4,2766 | 0,6553 | 3,6920 | 0,00142 | 0,0346 | **Spns3** |
| 664862 | 2941 | 2,1232 | 5,6642 | 3,6708 | 0,00149 | 0,0359 | **Gpr137b-ps** |
| 140580 | 5874 | 2,1034 | 6,9483 | 3,6693 | 0,00150 | 0,0359 | **Elmo1** |
| 16184 | 4428 | 2,5448 | 1,7917 | 3,6643 | 0,00152 | 0,0362 | **Il2ra** |
| 333433 | 4393 | 2,5833 | 3,7409 | 3,6633 | 0,00152 | 0,0362 | **Gpd1l** |
| 723848 | 102 | 8,3664 | -0,3880 | 3,6624 | 0,00152 | 0,0362 | **Mir34a** |
| 66783 | 1104 | 2,0742 | 3,1871 | 3,6562 | 0,00155 | 0,0365 | **4933437N03Rik** |
| 381667 | 685 | 3,0640 | -2,2612 | 3,6444 | 0,00159 | 0,0372 | **Gm1679** |
| 78917 | 516 | 4,1098 | 1,1949 | 3,6339 | 0,00163 | 0,0377 | **4930455G09Rik** |
| 100039452 | 657 | 3,8293 | -2,1275 | 3,6335 | 0,00163 | 0,0377 | **Gm2244** |
| 21952 | 1114 | 4,4707 | -1,1406 | 3,6328 | 0,00163 | 0,0377 | **Tnni1** |
| 18436 | 2441 | 11,3558 | 1,8063 | 3,6278 | 0,00165 | 0,0380 | **P2rx1** |
| 70375 | 3650 | 2,7716 | 1,4480 | 3,6223 | 0,00167 | 0,0382 | **Ica1l** |
| 13733 | 3245 | 2,4221 | 12,1873 | 3,6083 | 0,00173 | 0,0392 | **Emr1** |
| 106740 | 1088 | 2,8806 | 3,0675 | 3,5694 | 0,00189 | 0,0419 | **LOC106740** |
| 54614 | 3159 | 2,2087 | 4,4462 | 3,5663 | 0,00191 | 0,0421 | **Prpf40b** |
| 22164 | 1609 | 2,4905 | -0,0425 | 3,5489 | 0,00199 | 0,0435 | **Tnfsf4** |
| 171207 | 3321 | 4,8697 | 5,9068 | 3,5430 | 0,00201 | 0,0438 | **Arhgap4** |
| 109245 | 5230 | 4,4536 | 0,6591 | 3,5356 | 0,00205 | 0,0443 | **Lrrc39** |
| 67846 | 2934 | 2,0785 | 5,9478 | 3,5140 | 0,00215 | 0,0456 | **Tmem39a** |
| 16490 | 11582 | 4,1964 | 2,4207 | 3,5136 | 0,00216 | 0,0456 | **Kcna2** |
| 20307 | 511 | 2,2403 | 2,5292 | 3,4791 | 0,00234 | 0,0479 | **Ccl8** |
| 239790 | 1268 | 4,7862 | -0,8795 | 3,4737 | 0,00236 | 0,0483 | **Ostn** |
| 100041283 | 766 | 2,6857 | -0,3661 | 3,4613 | 0,00243 | 0,0495 | **Gm3252** |
