# Supplementary material for: Myeloid C/EBPβ deficiency reshapes microglial gene expression and is protective in experimental autoimmune encephalomyelitis
Source: J Neuroinflammation. 2017 Mar 16;14:54. doi: 10.1186/s12974-017-0834-5 (PMC5356255; doi:10.1186/s12974-017-0834-5)
Supplement: Additional file 1: Tables S1-S6. — List the genes significantly up-regulated (tables 1, 3 and 5) or down-regulated (tables 2, 4 and 6) by the absence of C/EBPβ in control (tables 1, 2), LPS-treated (tables 3, 4) and LPS+IFNγ-treated (tables 5, 6) primary microglial cultures. These data were obtained by RNAseq as described in Methods. (ZIP 253 kb) [file 12974_2017_834_MOESM1_ESM.zip › 12974_2017_834_MOESM1_ESM/Table S6.docx]

| **Table S6** |
| --- |
| **Genes with significantly decreased expression in LysMCre-CEBPbetafl/fl microglia in LPS+IFNg condition** |

| **GeneID** | **Length** | **FC** | **AveExpr** | **t** | **P.Value** | **adj P.value** | **gene_symbol** |
| --- | --- | --- | --- | --- | --- | --- | --- |
| 17105 | 1057 | 0,0045 | 7,2768 | -13,6533 | 1,13E-11 | 1,39E-07 | **Lyz2** |
| 56619 | 2519 | 0,0260 | 4,9510 | -13,2978 | 1,83E-11 | 1,39E-07 | **Clec4e** |
| 110796 | 5814 | 0,1047 | 3,9480 | -12,5538 | 5,20E-11 | 2,64E-07 | **Tshz1** |
| 56738 | 2644 | 0,1646 | 4,4779 | -11,7230 | 1,77E-10 | 5,88E-07 | **Mocs1** |
| 242960 | 3328 | 0,3020 | 6,0340 | -10,6725 | 9,19E-10 | 2,00E-06 | **Fbxl5** |
| 80719 | 2133 | 0,1374 | 6,3009 | -10,3201 | 1,64E-09 | 3,12E-06 | **Igsf6** |
| 19201 | 2262 | 0,2508 | 3,6770 | -10,1502 | 2,18E-09 | 3,69E-06 | **Pstpip2** |
| 16656 | 9021 | 0,1482 | 4,3214 | -9,9849 | 2,88E-09 | 4,39E-06 | **Hivep3** |
| 67338 | 4242 | 0,1592 | 4,3970 | -9,7828 | 4,07E-09 | 5,64E-06 | **Rffl** |
| 212307 | 4333 | 0,3892 | 5,6045 | -9,1329 | 1,28E-08 | 1,62E-05 | **Mapre2** |
| 215772 | 5218 | 0,2088 | 4,8739 | -8,7359 | 2,65E-08 | 2,88E-05 | **9130014G24Rik** |
| 245126 | 957 | 0,0835 | 1,5214 | -8,6421 | 3,15E-08 | 2,88E-05 | **Tarm1** |
| 211770 | 4026 | 0,3339 | 4,9829 | -8,6316 | 3,21E-08 | 2,88E-05 | **Trib1** |
| 16449 | 5493 | 0,1030 | 4,4196 | -8,5886 | 3,48E-08 | 2,94E-05 | **Jag1** |
| 73914 | 2969 | 0,1386 | 5,2863 | -8,5302 | 3,88E-08 | 2,96E-05 | **Irak3** |
| 231805 | 2588 | 0,2244 | 4,2906 | -8,1955 | 7,34E-08 | 5,08E-05 | **Pilra** |
| 14747 | 2845 | 0,1234 | 4,1051 | -8,1612 | 7,84E-08 | 5,08E-05 | **Cmklr1** |
| 19206 | 4305 | 0,2623 | 3,9018 | -8,1378 | 8,20E-08 | 5,08E-05 | **Ptch1** |
| 432466 | 1550 | 0,2318 | 4,4396 | -8,1292 | 8,34E-08 | 5,08E-05 | **Gm5424** |
| 14081 | 3891 | 0,3829 | 6,2145 | -8,0547 | 9,63E-08 | 5,64E-05 | **Acsl1** |
| 213053 | 5208 | 0,3120 | 6,3211 | -7,9647 | 1,15E-07 | 6,40E-05 | **Slc39a14** |
| 70097 | 7183 | 0,4236 | 8,2794 | -7,9518 | 1,18E-07 | 6,40E-05 | **Sash1** |
| 56613 | 3106 | 0,4199 | 6,0882 | -7,8557 | 1,42E-07 | 7,06E-05 | **Rps6ka4** |
| 171212 | 4702 | 0,3231 | 4,3441 | -7,8496 | 1,44E-07 | 7,06E-05 | **Galnt10** |
| 12608 | 1504 | 0,0304 | 3,4337 | -7,8032 | 1,58E-07 | 7,44E-05 | **Cebpb** |
| 329739 | 5702 | 0,3943 | 7,0030 | -7,7791 | 1,65E-07 | 7,44E-05 | **Fam102b** |
| 12182 | 2336 | 0,1058 | 1,5479 | -7,7766 | 1,66E-07 | 7,44E-05 | **Bst1** |
| 28105 | 4851 | 0,2381 | 3,5881 | -7,7598 | 1,72E-07 | 7,47E-05 | **Trim36** |
| 17158 | 6078 | 0,4773 | 8,0449 | -7,6321 | 2,21E-07 | 9,36E-05 | **Man2a1** |
| 76220 | 4292 | 0,2332 | 2,8378 | -7,5999 | 2,36E-07 | 9,72E-05 | **6530402F18Rik** |
| 14362 | 4378 | 0,1026 | 4,3520 | -7,5746 | 2,48E-07 | 9,95E-05 | **Fzd1** |
| 100043424 | 4492 | 0,2444 | 3,0493 | -7,4957 | 2,91E-07 | 0,0001 | **Gm14005** |
| 338365 | 4386 | 0,3811 | 4,0159 | -7,4674 | 3,08E-07 | 0,0001 | **Slc41a2** |
| 67712 | 4162 | 0,2186 | 5,0455 | -7,4026 | 3,51E-07 | 0,0001 | **Slc25a37** |
| 19730 | 4020 | 0,2197 | 6,1425 | -7,4024 | 3,52E-07 | 0,0001 | **Ralgds** |
| 12475 | 1497 | 0,3969 | 9,6500 | -7,3797 | 3,68E-07 | 0,0001 | **Cd14** |
| 26570 | 9227 | 0,2103 | 6,8229 | -7,3493 | 3,92E-07 | 0,0001 | **Slc7a11** |
| 75019 | 1665 | 0,0908 | -1,5389 | -7,3204 | 4,15E-07 | 0,0001 | **Rnase10** |
| 270084 | 2800 | 0,4081 | 7,3229 | -7,2921 | 4,40E-07 | 0,0001 | **Lpcat2** |
| 170744 | 3158 | 0,2324 | 6,3420 | -7,2544 | 4,75E-07 | 0,0001 | **Tlr8** |
| 23845 | 3820 | 0,2569 | 5,4696 | -7,1912 | 5,41E-07 | 0,0001 | **Clec5a** |
| 76441 | 6045 | 0,0639 | 2,0221 | -7,1115 | 6,38E-07 | 0,0002 | **Daam2** |
| 54683 | 688 | 0,3324 | 8,7332 | -7,0778 | 6,84E-07 | 0,0002 | **Prdx5** |
| 228608 | 2262 | 0,3882 | 5,8778 | -7,0387 | 7,42E-07 | 0,0002 | **Smox** |
| 170625 | 4447 | 0,2952 | 6,2915 | -7,0097 | 7,88E-07 | 0,0002 | **Snx18** |
| 14544 | 5417 | 0,1721 | 3,3991 | -6,9215 | 9,47E-07 | 0,0002 | **Gda** |
| 319565 | 21718 | 0,3509 | 5,9059 | -6,9157 | 9,59E-07 | 0,0002 | **Syne2** |
| 16911 | 2049 | 0,3430 | 5,2507 | -6,9106 | 9,69E-07 | 0,0002 | **Lmo4** |
| 23971 | 2600 | 0,3063 | 4,5466 | -6,8913 | 1,01E-06 | 0,0002 | **Papss1** |
| 12494 | 2995 | 0,1663 | 5,5751 | -6,7984 | 1,23E-06 | 0,0003 | **Cd38** |
| 11639 | 5162 | 0,3125 | 2,9864 | -6,7326 | 1,41E-06 | 0,0003 | **Ak4** |
| 12985 | 1363 | 0,0676 | 0,9178 | -6,6689 | 1,61E-06 | 0,0003 | **Csf3** |
| 19663 | 2780 | 0,2330 | 3,0620 | -6,6404 | 1,72E-06 | 0,0003 | **Rbpms** |
| 228071 | 8900 | 0,3217 | 5,4918 | -6,6206 | 1,79E-06 | 0,0003 | **Sestd1** |
| 241075 | 8562 | 0,3839 | 6,3655 | -6,5539 | 2,06E-06 | 0,0004 | **Plekhm3** |
| 269717 | 3851 | 0,2744 | 5,0424 | -6,5484 | 2,09E-06 | 0,0004 | **Orai2** |
| 22436 | 4587 | 0,3285 | 5,7895 | -6,5266 | 2,19E-06 | 0,0004 | **Xdh** |
| 23921 | 2848 | 0,1503 | 2,4702 | -6,5214 | 2,21E-06 | 0,0004 | **Sh2b2** |
| 66205 | 1257 | 0,4648 | 4,6004 | -6,5099 | 2,27E-06 | 0,0004 | **Cd302** |
| 100504452 | 3947 | 0,3951 | 4,7986 | -6,4366 | 2,66E-06 | 0,0004 | **Gm20235** |
| 171171 | 1770 | 0,1778 | 1,6140 | -6,4135 | 2,79E-06 | 0,0004 | **Ntng2** |
| 80879 | 2577 | 0,3902 | 6,2512 | -6,3340 | 3,31E-06 | 0,0005 | **Slc16a3** |
| 11988 | 7857 | 0,1933 | 7,3121 | -6,3339 | 3,32E-06 | 0,0005 | **Slc7a2** |
| 64292 | 3647 | 0,1031 | 5,7219 | -6,3316 | 3,33E-06 | 0,0005 | **Ptges** |
| 100604 | 2829 | 0,2091 | 3,6460 | -6,3306 | 3,34E-06 | 0,0005 | **Lrrc8c** |
| 12489 | 2571 | 0,2580 | 4,7300 | -6,2781 | 3,74E-06 | 0,0005 | **Cd33** |
| 20288 | 3998 | 0,3855 | 8,2773 | -6,2649 | 3,85E-06 | 0,0005 | **Msr1** |
| 70178 | 2238 | 0,4640 | 5,2092 | -6,2537 | 3,95E-06 | 0,0006 | **Fam108c** |
| 21664 | 1953 | 0,2584 | 3,7940 | -6,1764 | 4,67E-06 | 0,0006 | **Phlda1** |
| 270685 | 4332 | 0,3396 | 4,0186 | -6,1231 | 5,25E-06 | 0,0007 | **Mthfd1l** |
| 17474 | 1336 | 0,2823 | 8,3203 | -6,1100 | 5,41E-06 | 0,0007 | **Clec4d** |
| 16518 | 5448 | 0,2084 | 2,8187 | -5,9830 | 7,16E-06 | 0,0009 | **Kcnj2** |
| 18126 | 3990 | 0,4202 | 6,1173 | -5,9729 | 7,32E-06 | 0,0009 | **Nos2** |
| 80885 | 1947 | 0,4076 | 5,0045 | -5,9706 | 7,36E-06 | 0,0009 | **Niacr1** |
| 225372 | 2062 | 0,1627 | 2,9299 | -5,9579 | 7,57E-06 | 0,0009 | **Apbb3** |
| 104215 | 4122 | 0,3422 | 5,5028 | -5,9320 | 8,01E-06 | 0,0009 | **Rhoq** |
| 20656 | 3824 | 0,3775 | 8,8128 | -5,9034 | 8,54E-06 | 0,0010 | **Sod2** |
| 12986 | 4047 | 0,4094 | 6,8791 | -5,8948 | 8,70E-06 | 0,0010 | **Csf3r** |
| 73504 | 2504 | 0,1580 | 0,8583 | -5,8890 | 8,82E-06 | 0,0010 | **1700071M16Rik** |
| 75007 | 2834 | 0,3697 | 5,3195 | -5,8807 | 8,98E-06 | 0,0010 | **Fam63a** |
| 224794 | 4558 | 0,4704 | 5,5795 | -5,7287 | 1,26E-05 | 0,0013 | **Enpp4** |
| 69573 | 1106 | 0,2429 | 4,0754 | -5,7175 | 1,29E-05 | 0,0013 | **2310016C08Rik** |
| 20210 | 531 | 0,2375 | 6,7641 | -5,6524 | 1,50E-05 | 0,0015 | **Saa3** |
| 75767 | 7965 | 0,2618 | 5,6042 | -5,6468 | 1,52E-05 | 0,0015 | **Rab11fip1** |
| 19248 | 3166 | 0,3192 | 5,1261 | -5,5951 | 1,70E-05 | 0,0016 | **Ptpn12** |
| 75627 | 2125 | 0,3994 | 3,6987 | -5,5580 | 1,85E-05 | 0,0017 | **Snapc1** |
| 240354 | 5008 | 0,4290 | 7,7191 | -5,5465 | 1,90E-05 | 0,0018 | **Malt1** |
| 14289 | 1296 | 0,3831 | 6,4526 | -5,5225 | 2,01E-05 | 0,0018 | **Fpr2** |
| 619308 | 2893 | 0,2508 | 1,9003 | -5,4628 | 2,30E-05 | 0,0020 | **F830208F22Rik** |
| 11541 | 1844 | 0,2578 | 2,1233 | -5,4622 | 2,30E-05 | 0,0020 | **Adora2b** |
| 243197 | 2508 | 0,3172 | 3,4676 | -5,4551 | 2,34E-05 | 0,0020 | **Mfsd7a** |
| 353346 | 3433 | 0,0607 | -0,9769 | -5,4010 | 2,65E-05 | 0,0023 | **Gpr141** |
| 57248 | 878 | 0,1295 | 0,3387 | -5,3943 | 2,69E-05 | 0,0023 | **Ly6i** |
| 17319 | 554 | 0,3985 | 4,3736 | -5,3759 | 2,80E-05 | 0,0023 | **Mif** |
| 15937 | 1090 | 0,3526 | 5,7995 | -5,3571 | 2,92E-05 | 0,0024 | **Ier3** |
| 11847 | 1417 | 0,2258 | 2,0678 | -5,3500 | 2,97E-05 | 0,0024 | **Arg2** |
| 21462 | 2065 | 0,1679 | -0,1102 | -5,3430 | 3,02E-05 | 0,0024 | **Tcp10c** |
| 93730 | 3421 | 0,3853 | 4,3626 | -5,3417 | 3,03E-05 | 0,0024 | **Lztfl1** |
| 19739 | 8531 | 0,3330 | 1,8034 | -5,3381 | 3,05E-05 | 0,0024 | **Rgs9** |
| 15251 | 4761 | 0,4532 | 8,7240 | -5,3256 | 3,14E-05 | 0,0025 | **Hif1a** |
| 19267 | 5411 | 0,4815 | 5,7530 | -5,3007 | 3,33E-05 | 0,0026 | **Ptpre** |
| 14283 | 1705 | 0,0722 | -0,5112 | -5,2829 | 3,47E-05 | 0,0027 | **Fosl1** |
| 69716 | 2267 | 0,1420 | 1,1103 | -5,2750 | 3,53E-05 | 0,0027 | **Trip13** |
| 100861977 | 1664 | 0,1412 | 1,6212 | -5,2510 | 3,73E-05 | 0,0028 |  |
| 15275 | 4509 | 0,4033 | 5,3828 | -5,2284 | 3,93E-05 | 0,0029 | **Hk1** |
| 56615 | 943 | 0,2512 | 4,1810 | -5,2118 | 4,08E-05 | 0,0029 | **Mgst1** |
| 21682 | 2656 | 0,4104 | 4,9420 | -5,1388 | 4,82E-05 | 0,0032 | **Tec** |
| 12176 | 1756 | 0,4388 | 4,6152 | -5,1099 | 5,16E-05 | 0,0034 | **Bnip3** |
| 17387 | 2597 | 0,2344 | 7,4977 | -5,0804 | 5,52E-05 | 0,0035 | **Mmp14** |
| 66341 | 1305 | 0,3192 | 3,5754 | -5,0613 | 5,77E-05 | 0,0037 | **Eid3** |
| 11479 | 3277 | 0,3400 | 4,0083 | -5,0105 | 6,49E-05 | 0,0040 | **Acvr1b** |
| 68190 | 1815 | 0,4969 | 3,8712 | -4,9779 | 7,00E-05 | 0,0042 | **5330426P16Rik** |
| 15235 | 2286 | 0,1765 | 1,1307 | -4,9617 | 7,26E-05 | 0,0043 | **Mst1** |
| 93691 | 1344 | 0,4532 | 3,8468 | -4,9194 | 8,01E-05 | 0,0047 | **Klf7** |
| 58223 | 3453 | 0,3525 | 3,1983 | -4,8994 | 8,39E-05 | 0,0048 | **Mmp19** |
| 14936 | 3681 | 0,3491 | 3,5738 | -4,8678 | 9,03E-05 | 0,0051 | **Gys1** |
| 18024 | 2469 | 0,4614 | 7,1412 | -4,8291 | 9,88E-05 | 0,0054 | **Nfe2l2** |
| 16658 | 3389 | 0,2924 | 5,5809 | -4,8165 | 0,0001 | 0,0055 | **Mafb** |
| 545812 | 1807 | 0,2783 | 2,7641 | -4,7760 | 0,0001 | 0,0059 | **Pilrb2** |
| 17381 | 3607 | 0,4624 | 6,3779 | -4,7695 | 0,0001 | 0,0060 | **Mmp12** |
| 210104 | 3381 | 0,2146 | 2,7541 | -4,7281 | 0,0001 | 0,0064 | **Zfp658** |
| 29863 | 4127 | 0,3537 | 4,1108 | -4,6881 | 0,0001 | 0,0068 | **Pde7b** |
| 100340 | 1925 | 0,3060 | 3,1245 | -4,6800 | 0,0001 | 0,0068 | **Smpdl3b** |
| 225791 | 3321 | 0,3073 | 2,5518 | -4,6779 | 0,0001 | 0,0068 | **Zadh2** |
| 100504211 | 3928 | 0,4122 | 3,7053 | -4,6297 | 0,0002 | 0,0075 | **Gm20114** |
| 18793 | 1396 | 0,3955 | 6,5043 | -4,6226 | 0,0002 | 0,0076 | **Plaur** |
| 12894 | 4301 | 0,4171 | 6,3916 | -4,6120 | 0,0002 | 0,0077 | **Cpt1a** |
| 11303 | 10260 | 0,4341 | 9,2102 | -4,6060 | 0,0002 | 0,0078 | **Abca1** |
| 72144 | 4065 | 0,4908 | 4,8949 | -4,6003 | 0,0002 | 0,0078 | **Slc37a3** |
| 67603 | 2797 | 0,2711 | 5,0940 | -4,5859 | 0,0002 | 0,0080 | **Dusp6** |
| 69543 | 1015 | 0,1556 | 1,9843 | -4,5753 | 0,0002 | 0,0082 | **Capns2** |
| 64213 | 2239 | 0,4528 | 4,4346 | -4,5625 | 0,0002 | 0,0084 | **St7** |
| 21817 | 3549 | 0,2124 | 6,9447 | -4,5361 | 0,0002 | 0,0088 | **Tgm2** |
| 23796 | 3564 | 0,1540 | -1,5888 | -4,5336 | 0,0002 | 0,0088 | **Aplnr** |
| 78749 | 4044 | 0,3403 | 6,1649 | -4,5300 | 0,0002 | 0,0088 | **Filip1l** |
| 67742 | 1845 | 0,4608 | 5,1030 | -4,4749 | 0,0002 | 0,0098 | **Samsn1** |
| 231549 | 4199 | 0,4890 | 5,9337 | -4,4362 | 0,0002 | 0,0106 | **Lrrc8d** |
| 17394 | 2453 | 0,1147 | -0,1502 | -4,4150 | 0,0003 | 0,0109 | **Mmp8** |
| 52398 | 5008 | 0,2582 | 6,4928 | -4,3912 | 0,0003 | 0,0114 | **sep-11** |
| 106522 | 2454 | 0,3346 | 2,9323 | -4,3888 | 0,0003 | 0,0115 | **Pkdcc** |
| 27226 | 1915 | 0,2224 | 5,7935 | -4,3845 | 0,0003 | 0,0115 | **Pla2g7** |
| 170741 | 1020 | 0,3828 | 2,9790 | -4,3837 | 0,0003 | 0,0115 | **Pilrb1** |
| 74734 | 4960 | 0,4096 | 4,8033 | -4,3751 | 0,0003 | 0,0117 | **Rhoh** |
| 64095 | 4396 | 0,1972 | 3,2915 | -4,3625 | 0,0003 | 0,0119 | **Gpr35** |
| 100039078 | 659 | 0,1914 | -1,7905 | -4,3356 | 0,0003 | 0,0124 | **Gm2036** |
| 17386 | 2675 | 0,3013 | 8,9364 | -4,3097 | 0,0003 | 0,0129 | **Mmp13** |
| 219140 | 7517 | 0,3593 | 5,7724 | -4,2809 | 0,0004 | 0,0138 | **Spata13** |
| 224674 | 3634 | 0,4398 | 1,7283 | -4,2806 | 0,0004 | 0,0138 | **Slc37a1** |
| 320489 | 3837 | 0,3093 | 1,2881 | -4,2669 | 0,0004 | 0,0141 | **C530050E15Rik** |
| 13723 | 2622 | 0,2936 | 2,5350 | -4,2617 | 0,0004 | 0,0142 | **Emb** |
| 104709 | 3237 | 0,4820 | 6,1610 | -4,2540 | 0,0004 | 0,0145 | **Pik3r6** |
| 20238 | 10608 | 0,4617 | 5,3946 | -4,2510 | 0,0004 | 0,0145 | **Atxn1** |
| 14733 | 3552 | 0,1763 | 2,8971 | -4,2013 | 0,0004 | 0,0158 | **Gpc1** |
| 20311 | 1655 | 0,1527 | 1,2517 | -4,1887 | 0,0004 | 0,0160 | **Cxcl5** |
| 11812 | 540 | 0,1602 | 1,5174 | -4,1848 | 0,0004 | 0,0162 | **Apoc1** |
| 227737 | 3693 | 0,4833 | 7,2336 | -4,1760 | 0,0005 | 0,0164 | **Fam129b** |
| 16175 | 1974 | 0,3371 | 8,3743 | -4,1706 | 0,0005 | 0,0166 | **Il1a** |
| 74403 | 3611 | 0,2256 | -0,4359 | -4,1688 | 0,0005 | 0,0166 | **4933400F21Rik** |
| 100038411 | 1844 | 0,1216 | -0,9567 | -4,1655 | 0,0005 | 0,0166 | **Gm15998** |
| 231510 | 2877 | 0,1406 | -1,0561 | -4,1380 | 0,0005 | 0,0173 | **Agpat9** |
| 104079 | 2093 | 0,1795 | -1,1603 | -4,1334 | 0,0005 | 0,0174 | **Nxph3** |
| 100861753 | 1669 | 0,4364 | 3,2949 | -4,1320 | 0,0005 | 0,0174 |  |
| 100503468 | 1781 | 0,1150 | 2,4045 | -4,1181 | 0,0005 | 0,0179 | **Gm14023** |
| 20310 | 1083 | 0,1130 | 6,8974 | -4,1142 | 0,0005 | 0,0180 | **Cxcl2** |
| 58250 | 5528 | 0,3989 | 5,3132 | -4,1117 | 0,0005 | 0,0180 | **Chst11** |
| 22381 | 1018 | 0,4072 | 4,3429 | -4,0962 | 0,0006 | 0,0184 | **Wbp5** |
| 224796 | 5870 | 0,3232 | 2,6050 | -4,0922 | 0,0006 | 0,0184 | **Clic5** |
| 67647 | 5391 | 0,2995 | 1,0967 | -4,0561 | 0,0006 | 0,0194 | **4930523C07Rik** |
| 58217 | 3006 | 0,1215 | -0,0357 | -3,9992 | 0,0007 | 0,0213 | **Trem1** |
| 70358 | 1216 | 0,1021 | 0,2822 | -3,9881 | 0,0007 | 0,0215 | **Steap1** |
| 100502959 | 627 | 0,4922 | 3,3057 | -3,9752 | 0,0007 | 0,0221 | **AV051173** |
| 78057 | 624 | 0,2285 | -2,1102 | -3,9499 | 0,0008 | 0,0232 | **4930583I09Rik** |
| 625421 | 1461 | 0,4714 | 4,2791 | -3,9218 | 0,0008 | 0,0242 | **C230062I16Rik** |
| 20181 | 5267 | 0,4485 | 5,9747 | -3,9197 | 0,0008 | 0,0243 | **Rxra** |
| 16181 | 2691 | 0,3532 | 7,2584 | -3,8955 | 0,0009 | 0,0252 | **Il1rn** |
| 66412 | 3955 | 0,3836 | 6,9058 | -3,8649 | 0,0009 | 0,0265 | **Arrdc4** |
| 11622 | 5494 | 0,4003 | 3,7457 | -3,8636 | 0,0010 | 0,0265 | **Ahr** |
| 59010 | 2102 | 0,4650 | 5,0960 | -3,8497 | 0,0010 | 0,0269 | **Sqrdl** |
| 76954 | 4526 | 0,3558 | 3,8463 | -3,8345 | 0,0010 | 0,0276 | **St5** |
| 381310 | 6557 | 0,2465 | 0,3466 | -3,8178 | 0,0011 | 0,0284 | **6330403A02Rik** |
| 210789 | 6641 | 0,3693 | 5,1366 | -3,7968 | 0,0011 | 0,0293 | **Tbc1d4** |
| 54483 | 3281 | 0,2078 | 1,0504 | -3,7824 | 0,0011 | 0,0300 | **Mefv** |
| 29877 | 5868 | 0,3204 | 3,1447 | -3,7773 | 0,0012 | 0,0303 | **Hdgfrp3** |
| 414801 | 3766 | 0,4054 | 4,3708 | -3,7763 | 0,0012 | 0,0303 | **Itprip** |
| 100503085 | 2142 | 0,1918 | -1,2940 | -3,7680 | 0,0012 | 0,0306 | **LOC100503085** |
| 76770 | 528 | 0,2165 | -1,7030 | -3,7653 | 0,0012 | 0,0307 | **2010005H15Rik** |
| 11853 | 1056 | 0,4795 | 6,5081 | -3,7487 | 0,0012 | 0,0317 | **Rhoc** |
| 238037 | 3180 | 0,2832 | 1,3680 | -3,7388 | 0,0013 | 0,0322 | **BC068281** |
| 72230 | 3839 | 0,2740 | 0,8656 | -3,7371 | 0,0013 | 0,0322 | **Zfp558** |
| 14745 | 3522 | 0,1218 | 1,6317 | -3,7350 | 0,0013 | 0,0323 | **Lpar1** |
| 228662 | 5111 | 0,3124 | 1,9435 | -3,7219 | 0,0013 | 0,0330 | **Btbd3** |
| 218639 | 3361 | 0,3613 | 1,5100 | -3,6870 | 0,0014 | 0,0349 | **Arl15** |
| 20725 | 3289 | 0,4528 | 3,7549 | -3,6754 | 0,0015 | 0,0357 | **Serpinb8** |
| 269695 | 4481 | 0,1764 | -0,3070 | -3,6697 | 0,0015 | 0,0359 | **Rnft2** |
| 14538 | 7039 | 0,4749 | 4,4936 | -3,6540 | 0,0016 | 0,0366 | **Gcnt2** |
| 50527 | 4418 | 0,4589 | 5,4066 | -3,6391 | 0,0016 | 0,0376 | **Ero1l** |
| 20779 | 3887 | 0,3056 | 6,3659 | -3,6368 | 0,0016 | 0,0377 | **Src** |
| 226101 | 7094 | 0,3725 | 6,5993 | -3,6366 | 0,0016 | 0,0377 | **Myof** |
| 244237 | 2977 | 0,3262 | 1,6267 | -3,6263 | 0,0017 | 0,0380 | **Tnfrsf26** |
| 12544 | 2143 | 0,2175 | 0,0205 | -3,6234 | 0,0017 | 0,0381 | **Cdc45** |
| 13121 | 4353 | 0,4629 | 5,0793 | -3,6212 | 0,0017 | 0,0382 | **Cyp51** |
| 18799 | 2651 | 0,4241 | 2,5085 | -3,5906 | 0,0018 | 0,0408 | **Plcd1** |
| 225283 | 4297 | 0,3446 | 3,0011 | -3,5891 | 0,0018 | 0,0408 | **Rprd1a** |
| 23871 | 5060 | 0,3406 | 4,4724 | -3,5865 | 0,0018 | 0,0409 | **Ets1** |
| 20660 | 6938 | 0,4895 | 6,7154 | -3,5550 | 0,0020 | 0,0432 | **Sorl1** |
| 320189 | 3029 | 0,1691 | -0,1410 | -3,5495 | 0,0020 | 0,0435 | **9430076C15Rik** |
| 100503651 | 1772 | 0,2837 | 3,0798 | -3,5487 | 0,0020 | 0,0435 | **Gm15354** |
| 56702 | 672 | 0,2093 | 0,9131 | -3,5485 | 0,0020 | 0,0435 | **Hist1h1b** |
| 66569 | 2484 | 0,3592 | 3,2234 | -3,5475 | 0,0020 | 0,0435 | **Gdpd1** |
| 399558 | 7099 | 0,3440 | 5,1141 | -3,5420 | 0,0020 | 0,0439 | **Flrt2** |
| 380614 | 6114 | 0,4937 | 4,3959 | -3,5139 | 0,0022 | 0,0456 | **Intu** |
| 227671 | 1335 | 0,2765 | 2,0461 | -3,5033 | 0,0022 | 0,0464 | **Gbgt1** |
| 65099 | 1893 | 0,2542 | -0,2086 | -3,5019 | 0,0022 | 0,0465 | **Irak1bp1** |
| 109135 | 5133 | 0,4344 | 3,0207 | -3,4984 | 0,0022 | 0,0468 | **Plekha5** |
| 19204 | 1820 | 0,4496 | 6,0985 | -3,4965 | 0,0022 | 0,0470 | **Ptafr** |
| 216285 | 2493 | 0,1637 | -1,4483 | -3,4825 | 0,0023 | 0,0478 | **Alx1** |
| 70435 | 4601 | 0,4186 | 3,6658 | -3,4654 | 0,0024 | 0,0491 | **Inf2** |
