# Supplementary figures and images for: Myeloid C/EBPβ deficiency reshapes microglial gene expression and is protective in experimental autoimmune encephalomyelitis
Source: J Neuroinflammation. 2017 Mar 16;14:54. doi: 10.1186/s12974-017-0834-5 (PMC5356255; doi:10.1186/s12974-017-0834-5)

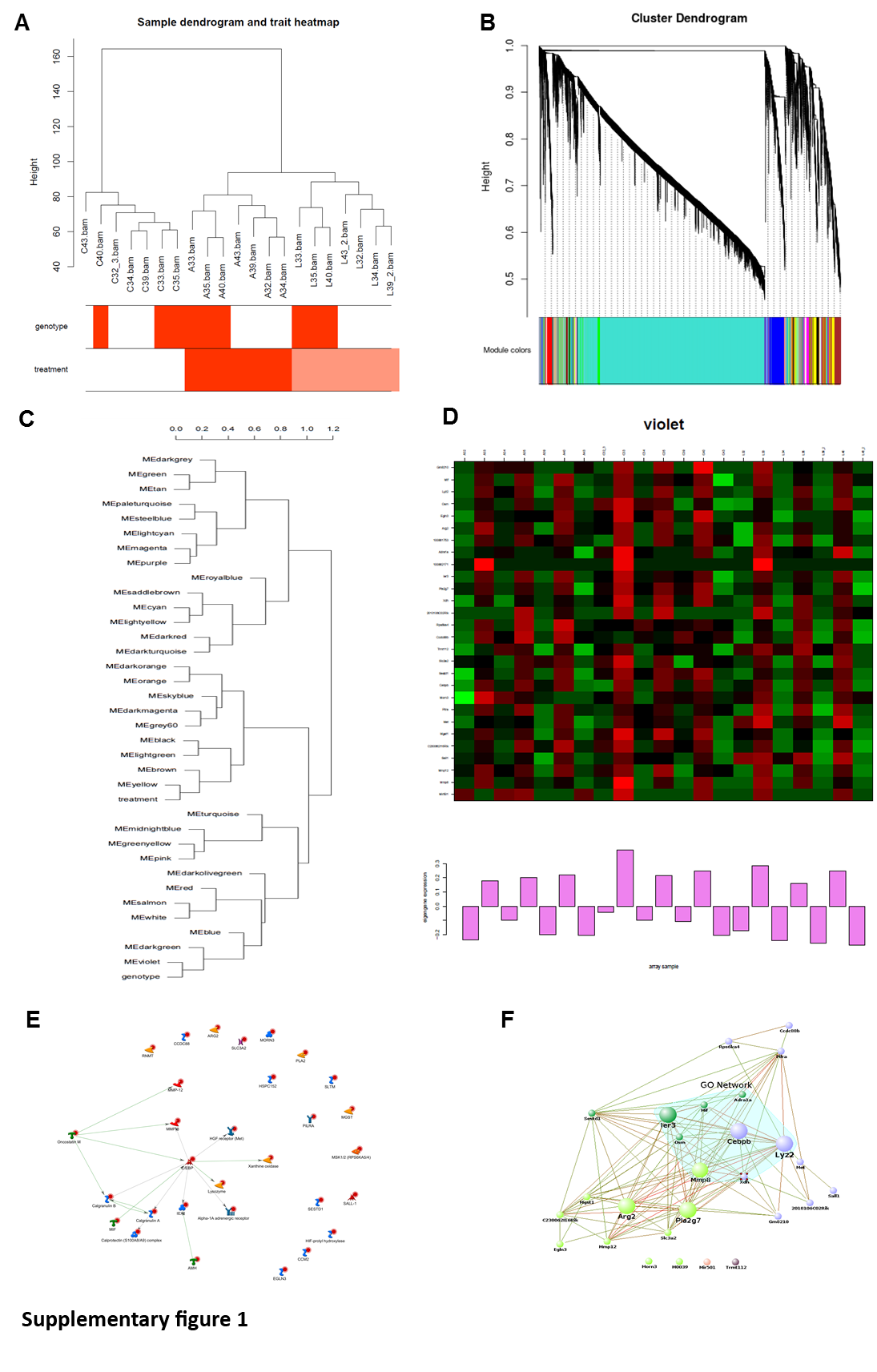

Supplement: Additional file 2: Figure S1. — Weighted Gene Correlation Network Analyses of microglial activation, treatment and LysMCre-C/EBPβfl/fl phenotype. A) Correlation dendrogram of genes. WGCNA algorithm was applied to filtered expression of all samples, a soft-threshold for the similarity matrix of β = 9 was used, and module detection was obtained with a dynamic tree cut; color bar down of the dendrogram shows module pertainance of genes with a large turquoise module corresponding to genes downregulated upon treatment with either LPS or LPS + IFNγ. B) Hierarchical clustering of detected modules by WGCNA and genotype and treatment traits; module MEyellow is the closest group of genes related to the treatment trait, whereas MEviolet is for genotype. C) Module violet heatmap (top) and eigengene expression graph (bottom): module violet is the closest group of genes related to genotype effect, contains C/EBPβ and Lyz2. D) Metacore™ network obtained by literature described interactions among the genes in module violet evidences a network of genes described to interact with C/EBPβ as a central hub. E) Correlation network obtained with a threshold for distance of 0.3, with C/EBPβ as a central hub (node size relative to degree of node). (TIF 1462 kb) [file 12974_2017_834_MOESM2_ESM.tif]
